# Trends for prevalence and incidence of resistant hypertension: population based cohort study in the UK 1995-2015

**DOI:** 10.1136/bmj.j3984

**Published:** 2017-09-22

**Authors:** Sarah-Jo Sinnott, Liam Smeeth, Elizabeth Williamson, Ian J Douglas

**Affiliations:** 1 Department of non-communicable disease epidemiology, London School of Hygiene & Tropical Medicine, London, WC1E7HT, UK; 2 Department of Medical Statistics, London School of Hygiene & Tropical Medicine, London, UK

## Abstract

**Objective** To estimate the incidence and prevalence of resistant hypertension among a UK population treated for hypertension from 1995 to 2015.

**Design** Cohort study.

**Setting** Electronic health records from the UK Clinical Practice Research Datalink in primary care.

**Participants** 1 317 290 users of antihypertensive drugs with a diagnosis of hypertension.

**Main outcome measures** Resistant hypertension was defined as concurrent use of three antihypertensive drugs inclusive of a diuretic, uncontrolled hypertension (≥140/90 mm Hg), and adherence to the prescribed drug regimen, or concurrent use of four antihypertensive drugs inclusive of a diuretic and adherence to the prescribed drug regimen. To determine incidence, the numerator was new cases of resistant hypertension and the denominator was person years of those with treated hypertension and at risk of developing resistant hypertension. To determine prevalence, the numerator was total number of cases with resistant hypertension and the denominator was those with treated hypertension. Prevalence and incidence were age standardised to the 2015 hypertensive population.

**Results** The age standardised incidence of resistant hypertension increased from 0.93 cases per 100 person years (95% confidence interval 0.87 to 1.00) in 1996 to a peak level of 2.07 cases per 100 person years (2.03 to 2.12) in 2004. Incidence then decreased to 0.42 cases per 100 person years (0.40 to 0.44) in 2015. Age standardised prevalence increased from 1.75% (95% confidence interval 1.66% to 1.83%) in 1995 to a peak of 7.76% (7.70% to 7.83%) in 2007. Prevalence then plateaued and subsequently declined to 6.46% (6.38% to 6.54%) in 2015. Compared with patients aged 65-69 years, those aged 80 or more years were more likely to have prevalent resistant hypertension throughout the study period.

**Conclusions** Prevalent resistant hypertension has plateaued and decreased in recent years, consistent with a decrease in incidence from 2004 onwards. Despite this, resistant hypertension is common in the UK hypertensive population. Given the importance of hypertension as a modifiable risk factor for cardiovascular disease, reducing uncontrolled hypertension should remain a population health focus.

## Introduction

Uncontrolled hypertension is a leading risk factor for cardiovascular disease related morbidity and deaths.^1^ Hypertension is now so widely prevalent, affecting one billion people worldwide and directly responsible for more than 10 million deaths per year, that it has been declared a global public health crisis by the World Health Organization.^1^ ^2^ Resistant hypertension is blood pressure ≥140/90 mm Hg despite treatment with optimal doses of three different antihypertensive drugs, one of which should be a diuretic.^3^ ^4^ ^5^ In instances where an individual’s blood pressure is at target levels but four or more antihypertensive drugs are required, resistant hypertension can also be defined.^4^ Those with resistant hypertension have double the risk of cardiovascular events than those without resistant hypertension, thus making them an important group to study.^6^

Current evidence from a systematic review and meta-analysis of 24 studies estimates the prevalence of resistant hypertension to be between 14% and 16% of all patients with hypertension, equalling 140-160 million people globally.^7^ These estimates may be biased upwards for two reasons. Firstly, four randomised studies were included, which likely overestimated prevalence owing to selected patients at high cardiovascular risk involved in trials.^8^ Secondly, of the 20 observational studies included, which should reflect the real world burden of resistant hypertension more so than randomised studies, few assessed adherence to antihypertensive drugs. Non-adherence has been found to be the cause of uncontrolled hypertension in as many as 50% of patients with supposed resistant hypertension.^9^ ^10^ ^11^ One previous observational study, based on US claims data, estimated the incidence of resistant hypertension at 1.9%.^6^ However, this estimate was based on data from 2002-06 and requires updating. Additionally, assessing the burden of resistant hypertension outside settings already studied offers benefits in terms of wider generalisability.^7^ ^11^ ^12^ ^13^

Thus an up-to-date epidemiological study on the burden of resistant hypertension, accounting for adherence to antihypertensive drug treatment is required. Accordingly, we measured the trends in incidence and prevalence of resistant hypertension among those with treated hypertension between 1995 and 2015 in the UK primary care setting.

## Methods

### Study design and data

We conducted a retrospective cohort study, using the Clinical Practice Research Database (CPRD-GOLD); a nationally representative repository of deidentified electronic health records from primary care in the UK. CPRD-GOLD holds data on personal information, health related behaviours, test results, diagnoses, and prescriptions for more than 11 million people in more than 670 practices across the UK since 1987.^14^ It is one of the largest databases of longitudinal medical records from primary care globally and has been extensively validated.^14^ ^15^ Data quality are monitored by CPRD internal processes.

### Population

We identified users of antihypertensive drugs between 1995 and 2015. In CPRD data, prescriptions issued by the general practitioner are automatically recorded with a product name and number. We used product numbers to identify antihypertensive drugs (see supplementary material 1) and categorised these into 14 classes: vasodilators, centrally acting agents, adrenergic blockers, α blockers, angiotensin converting enzyme inhibitors, angiotensin receptor blockers, renin inhibitors, thiazide diuretics, loop diuretics, potassium sparing diuretics (eg, amiloride), aldosterone antagonists (eg, spironolactone), β blockers, calcium channel blockers, and other. Preparations of fixed dose combinations were split into their constituent active ingredients, so that patients were attributed with exposure to each different category of drug. Eligibility for the study began on the latest of: the patient’s 18th birthday, the date a patient’s practice was deemed to contribute “up-to-standard” data plus one year, the patient’s “current registration” date plus one year, and the patients’ earliest date of hypertension diagnosis. We required a diagnosis of hypertension (see Read codes in supplementary material 2) in addition to drug use because this method gives similar estimates for treated hypertension to those from the health survey for England.^16^ Eligibility ended on the earliest of the date on which the patient died, the date on which the patient transferred out of practice, the last data collection date from practice, and the study end date (31 December 2015).

### Case definition

Clinical guidelines define resistant hypertension in two ways: uncontrolled hypertension (≥140/90 mm Hg) during treatment with and adherence to three concurrent antihypertensive drugs, inclusive of a diuretic, and treatment with and adherence to four antihypertensive drugs used concurrently.^3^ ^4^ ^5^

To identify patients who met the three drug definition we used a multistep process. Firstly, we found users of one antihypertensive drug and followed forward to assess whether they were prescribed a second drug and a third drug within different drug categories. To ensure that patients with hypertension were using three different drugs concurrently, as opposed to switching drugs, we required that patients starting a third antihypertensive drug had repeat prescriptions for all three drugs within six months of initiating the third.

Secondly, in patients who had evidence of concurrent treatment with three antihypertensive drugs, inclusive of a diuretic, we required a blood pressure reading of systolic blood pressure ≥140 mm Hg or diastolic blood pressure ≥90 mm Hg within 12 months of starting the third drug.^3^ ^4^ ^5^ Patients with blood pressure readings <140/90 mm Hg did not meet the criteria for resistant hypertension and were excluded from our case definition at this point but could be considered at a future date if new drug changes occurred. The index date—the date on which resistant hypertension could be identified—was confirmed as the later date of evidence of concurrent use of three drugs or the date of high blood pressure reading.

Thirdly, we required patients to show good adherence to their antihypertensive drug regimen. In the absence of dispensing records, which are typically used to measure adherence at the population level, we instead used prescribing records to estimate a proxy for drug adherence. Going backwards one year from the index date, we measured proxy adherence from the date of the first prescription within this one year period until the index date or until the discontinuation date of that drug, if before the index date. Discontinuation was defined as a gap of 90 or more days between the expected finishing date of a prescription and the end of the adherence observation period (ie, the index date). Using prescription dates and computed days’ supply prescribed, we calculated proxy adherence as the number of days covered by the drug divided by the number of days in the observation period. We accounted for leftover days’ supply from previous prescriptions by adding to the next supply. Once proxy adherence was measured for all antihypertensive drugs, we calculated an average across all drugs for each patient.^17^ Binary adherence was defined as an average proxy adherence of 80% or more.^18^ If patients did not meet our definition for proxy adherence, they were excluded from the case definition of resistant hypertension. We classified patients as meeting the case definition of resistant hypertension at the earliest opportunity, regardless of whether or not the patient went on to be a concurrent user of four drugs.

Patients prescribed four different antihypertensive agents, inclusive of a diuretic, were included in the case definition in a similar process. First, we required patients starting a fourth antihypertensive drug to have repeat prescriptions for all four drugs within six months of initiating the fourth drug. We did not require a blood pressure reading for those taking four drugs in concordance with accepted definitions of resistant hypertension.^4^ We assessed adherence in the same way as described previously. The index date was the earliest date within the six month period that there was evidence of concurrent use of four drugs.

### Analysis of incidence and prevalence

We calculated annual incidence by dividing the number of new cases of resistant hypertension in each study year by the number of person years contributed by patients with treated hypertension at risk of developing resistant hypertension in that year. The denominator included patients who were excluded from the case definition on the basis of having poor adherence. We calculated annual prevalence by dividing the number of all live patients with resistant hypertension at the end of each year by the number with treated hypertension who remained eligible at the end of that year.

We calculated crude annual rates of incidence and prevalence per 100 person years or 100 people, with 95% confidence intervals. To account for a changing age structure over time, we used direct standardisation by applying age specific rates in each year to the standard population, which was the 2015 hypertensive population in CPRD. We used Poisson models with interaction terms between calendar year and sex and between calendar year and age category to assess whether the effect of sex or age on incidence and prevalence varied over time (Wald P values for model). Within a data driven approach, we used Joinpoint models to analyse age standardised trends over time.^19^ These models select an appropriate number of “turns” in trend data and give corresponding slopes to each segment in the trend. The optimal number of turns is selected by comparing permutation test results between multiple Joinpoint models.

### Sensitivity analyses

We carried out three sensitivity analyses. The first was to assess the influence of defining proxy adherence at 70%, and not accounting for adherence. The second assessed how the three drug definition and the four drug definition of resistant hypertension impacted on trends. Thirdly, to accommodate that different blood pressure thresholds have been used in the management of hypertension over time, and for different subgroups, we ran an analysis between 1995 and 2000 where we defined uncontrolled hypertension as blood pressure ≥160/90 mm Hg^20^ and ran an analysis for those aged 80 or more years where we defined uncontrolled hypertension as blood pressure ≥150/90 mm Hg.^3^ All analyses were carried out using Stata MP Version 14.2 and Joinpoint Regression Programme Version 4.4.0.0.^19^ ^21^

### Patient involvement

No patients were involved in setting the research question, nor were they involved in developing plans for recruitment, design, or implementation of the study. No patients were asked to advise on interpretation or writing up of results. There are no plans to disseminate the results of the research to study participants.

## Results

### Study population

More than 1.3 million patients met our inclusion criteria for the study (fig 1). Of the total CPRD population, 16.46% (95% confidence interval 15.96% to 16.96%) were treated for hypertension. Background hypertension closely aligned with national estimates for treated hypertension over time from health survey for England data (see supplementary material 3).

**Figure f1:**
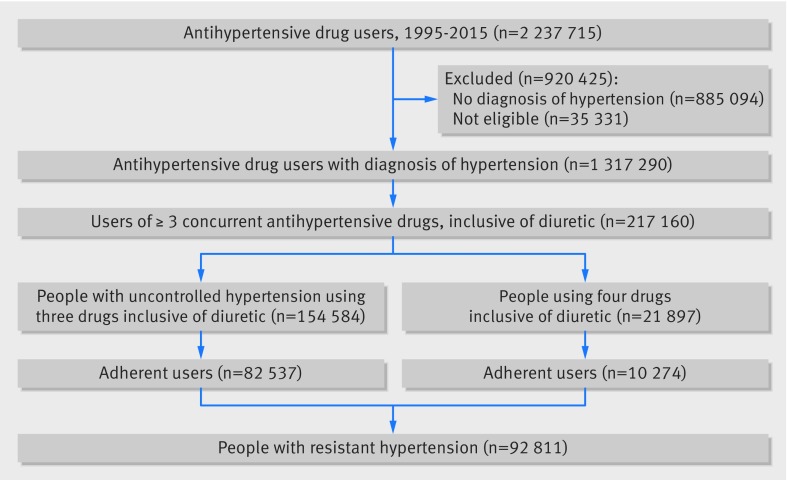
**Fig 1** Flowchart of cohort identification

### Incidence 

During the study period, 90 973 new cases of resistant hypertension occurred in the hypertensive population (average non-weighted incidence 1.20 per 100 person years, 95% confidence interval 0.77 to 1.79).

*Temporal trend*—incidence of resistant hypertension increased from 0.93 (0.87 to 1.00) per 100 person years in 1996 to 2.01 cases (1.96 to 2.07) in 2001 (annual percentage change 21.49%, 95% confidence interval 13.13% to 30.48%, table 1 and supplementary material 4). Incidence peaked at 2.07 cases (95% confidence interval 2.03 to 2.12) per 100 person years in 2004 (table 1 and supplementary material 4). Incidence then declined between 2004 and 2009 (annual percentage change −17.61%, −21.15% to −13.09%) and less rapidly between 2009 and 2015 (−10.19%, −13.62% to −6.64%) to reach 0.42 cases (95% confidence interval 0.40 to 0.44) per 100 person years (table 1 and supplementary material 4).

**Table 1 tbl1:** Incidence and prevalence of resistant hypertension, 1995-2016

Year	No of incident resistant hypertension cases	Person years	Crude incidence per 100 person years (95% CI)	Standardised* incidence per 100 person years to 2015 population	No of prevalent resistant hypertension cases	No of prevalent hypertensive patients	Crude prevalence per 100 people (95% CI)	Standardised* prevalence to 2015 population
1995					1657	90 885	1.82 (0.95 to 2.70)	1.75 (1.66 to 1.83)
1996	944	97 842.4	0.96 (0.35 to 1.58)	0.93 (0.87 to 1.00)	2476	10 2534	2.41 (1.46 to 3.37)	2.31 (2.22 to 2.40)
1997	912	114 684.9	0.79 (0.28 to 1.31)	0.78 (0.72 to 0.83)	3217	122 552	2.63 (1.72 to 3.53)	2.52 (2.43 to 2.60)
1998	1250	137 338.3	0.91 (0.41 to 1.41)	0.88 (0.83 to 0.93)	4236	147 899	2.86 (2.00 to 3.73)	2.74 (2.66 to 2.82)
1999	2152	1 649 24.6	1.30 (0.75 to 1.86)	1.26 (1.20 to 1.31)	6077	180 905	3.36 (2.51 to 4.20)	3.21 (3.13 to 3.29)
2000	3836	211 231.4	1.81 (1.24 to 2.39)	1.75 (1.70 to 1.81)	9424	238 657	3.95 (3.15 to 4.75)	3.79 (3.71 to 3.86)
2001	5755	276 205.0	2.08 (1.54 to 2.62)	2.01 (1.96 to 2.07)	14 475	303 426	4.77 (3.99 to 5.55)	4.58 (4.50 to 4.65)
2002	6808	328 707.7	2.07 (1.58 to 2.56)	2.00 (1.96 to 2.05)	20 158	362 534	5.56 (4.79 to 6.33)	5.34 (5.26 to 5.41)
2003	8266	392 679.0	2.10 (1.65 to 2.56)	2.04 (2.00 to 2.09)	26 975	433 536	6.22 (5.48 to 6.96)	5.97 (5.90 to 6.04)
2004	9706	453 736.8	2.14 (1.71 to 2.56)	2.07 (2.03 to 2.12)	34 779	502 592	6.92 (6.19 to 7.65)	6.63 (6.56 to 6.70)
2005	9046	507 018.8	1.78 (1.41 to 2.15)	1.73 (1.70 to 1.77)	41 517	555 801	7.47 (6.75 to 8.19)	7.15 (7.08 to 7.22)
2006	7684	532 992.9	1.44 (1.12 to 1.76)	1.39 (1.36 to 1.43)	45 993	579 957	7.93 (7.21 to 8.66)	7.56 (7.49 to 7.63)
2007	6638	548 982.9	1.21 (0.92 to 1.50)	1.17 (1.14 to 1.20)	48 720	594 430	8.20 (7.47 to 8.92)	7.76 (7.70 to 7.83)
2008	5424	562 479.1	0.96 (0.71 to 1.22)	0.94 (0.91 to 0.96)	50 074	613 436	8.16 (7.45 to 8.88)	7.70 (7.63 to 7.76)
2009	4630	572 852.2	0.81 (0.57 to 1.04)	0.79 (0.76 to 0.81)	50 093	618 126	8.10 (7.39 to 8.81)	7.61 (7.54 to 7.67)
2010	4253	572 209.5	0.74 (0.52 to 0.96)	0.72 (0.70 to 0.74)	49 080	612 663	8.01 (7.30 to 8.72)	7.49 (7.42 to 7.55)
2011	3656	562 208.6	0.65 (0.44 to 0.86)	0.63 (0.61 to 0.65)	47 624	601 090	7.92 (7.21 to 8.63)	7.36 (7.29 to 7.42)
2012	3245	557 515.6	0.58 (0.38 to 0.78)	0.56 (0.54 to 0.58)	45 680	589 656	7.75 (7.04 to 8.46)	7.15 (7.08 to 7.21)
2013	2752	525 711.3	0.52 (0.33 to 0.72)	0.51 (0.49 to 0.53)	41 409	543 900	7.61 (6.88 to 8.35)	6.98 (6.92 to 7.05)
2014	2243	476 279.0	0.47 (0.27 to 0.66)	0.46 (0.44 to 0.48)	34 678	477 887	7.26 (6.49 to 8.02)	6.62 (6.55 to 6.69)
2015	1773	406 033.6	0.43 (0.23 to 0.63)	0.42 (0.40 to 0.44)	26 877	379 686	7.08 (6.23 to 7.93)	6.46 (6.38 to 6.54)

*Standardised to age distribution of 2015 hypertensive population.

### Age and sex

Those aged more than 70 years were more likely than those aged 65-69 years to develop incident resistant hypertension during the study period: incidence rate ratios 1.12 (95% confidence interval 1.09 to 1.14) for those aged 70-74 years, 1.19 (1.16 to 1.23) for those aged 75-79 years, and 1.07 (1.04 to 1.10) for those aged 80 or more years (see supplementary material 5). Women were 2% less likely to develop incident resistant hypertension than men (0.98, 0.96 to 0.99, see supplementary material 5).

We found no evidence showing that the effect of sex or age on incidence varied over time (fig 2).

**Figure f2:**
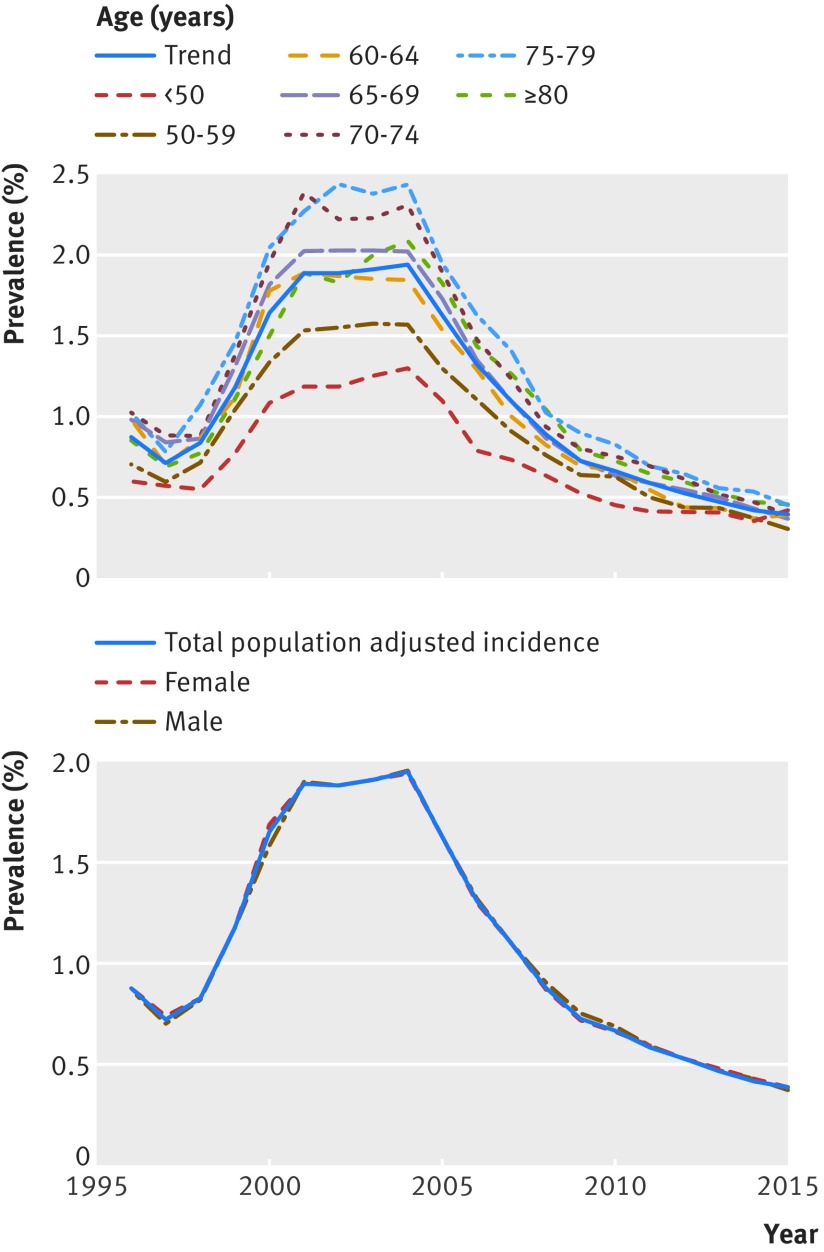
**Fig 2** Incidence stratified by age category and sex, 1996-2016

### Prevalence

*Temporal trend*—there was an increase from 1.75 prevalent cases (95% confidence interval 1.66 to 1.83) per 100 people in 1995 to 6.46 cases (6.38 to 6.54) per 100 people with hypertension in 2015 (table 1). This increase was not linear. Between 1995 and 2003 the annual percentage change was 16.30% (95% confidence interval 14.98% to 17.64%, P<0.001). This changed to 8.15% (4.06% to 12.40%, P<0.001) between 2003 and 2006, peaking at 7.76 cases (95% confidence interval 7.70 to 7.83) in 2007 before a brief plateau occurred. Thereafter, prevalence decreased to 6.46 cases in 2015, with an annual percentage change of −3.08% (95% confidence interval −3.93% to −2.22%, P<0.001) (see supplementary material 4).

*Age and sex*—older people were more likely to be prevalent cases. The prevalence in those aged 80 or more years was 1.43 times (95% confidence interval 1.39 to 1.46) that of those aged 65-69 years (see supplementary material 5). Over time, people aged 80 or more years were more likely to have prevalent resistant hypertension with each passing year from the early 2000s onwards (P<0.001) (fig 3). We found no evidence showing that the effect of sex on prevalence varied over time (fig 3).

**Figure f3:**
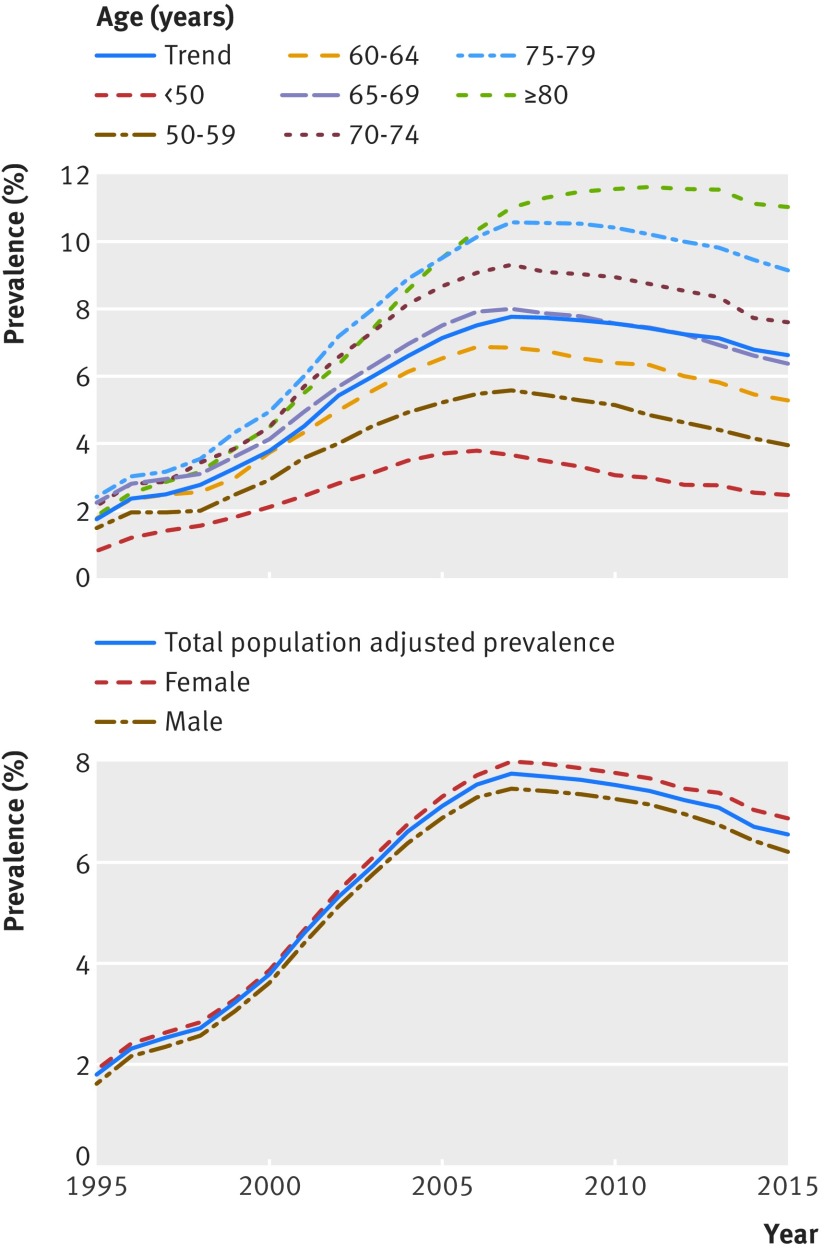
**Fig 3** Prevalence stratified by age category and sex, 1995-2016

### Sensitivity analyses

When proxy adherence was defined at a 70% threshold, or not accounted for, trends mirrored those defined at an 80% threshold, albeit with higher peak incidences in 2004 and peak prevalences in 2007, respectively (see supplementary material 6). Trends in incidence and prevalence were driven by the three drug definition, although the prevalence of four drug use has increased over time (see supplementary material 7). When hypertension was defined using a ≥160/90 mm Hg threshold in the 1990s, we found similar trends to the main analysis but at lower absolute levels (see supplementary material 8). When hypertension was defined as ≥150/90 mm Hg for those aged 80 years or more, prevalence still increased over time, although not as dramatically as when hypertension was defined as ≥140/90 mm Hg (see supplementary material 8).

## Discussion

In this longitudinal cohort study of more than 1.3 million people treated for hypertension, the incidence of resistant hypertension increased steeply between the late 1990s and the mid-2000s, and peaked at approximately two cases per 100 person years in 2004. Thereafter, the incidence decreased to 0.4 cases per 100 person years in 2015. Prevalence increased steadily from 1.8% in 1995, peaking at 7.8% in 2007. Thereafter, the trend declined gently, reaching 6.5% in 2015. Some evidence showed that those aged 80 or more years were more likely than those aged 65-69 years to have prevalent resistant hypertension over the study period.

### Strengths and weaknesses of this study

This is the largest longitudinal study to assess the prevalence and incidence of resistant hypertension to date. We included more than 1.3 million users of antihypertensive drugs with a diagnosis of hypertension. This definition ensured the use of antihypertensive drugs in those with hypertension, although not all blood pressure lowering drugs may have been intended for the treatment of hypertension. None the less, the prevalence of treated hypertension in our study closely matched national prevalence estimates for treated hypertension in the health survey for England (see supplementary material 3). In the absence of dispensing data, we used prescribing data within CPRD to estimate a proxy measure of adherence. Compared with dispensing data, prescribing data can overestimate adherence to cardiovascular drugs, from 5%^22^ to 20%.^23^ This may have impacted our results by overestimating the number of patients who were adherent, thus inflating prevalence and incidence. Assuming a worst case scenario whereby our figures represent an overestimation of 20%, peak prevalence would then be approximately 6% and peak incidence would be approximately 1.7 cases per 100 person years. Thus we acknowledge the limitations of our method but believe our estimates are strengthened by using what was the only available approach for assessing adherence in our population level data source. Furthermore, our various sensitivity analyses on adherence give a range of estimates to inform interpretation. We used clinic blood pressure measurements in this study, which are susceptible to white coat hypertension. However, we believe the trends we have reported are reflective of real world patterns, based on treatment decisions and management pathways as they occur in reality in patients with established hypertension. We used one blood pressure measurement to ascertain whether patients had controlled or uncontrolled hypertension. Previous research found that blood pressure measurements in electronic health records are representative of adjacent measurements^24^ and that using two measurements confers no advantage over one measurement in identifying patients with resistant hypertension.^25^ Another source of pseudo-resistant hypertension is secondary causes; we found these to be negligibly distributed among those with resistant hypertension (see supplementary material 9).

We used a general threshold of blood pressure ≥140/90 mm Hg to define hypertension at the population level, which is the accepted threshold in national and international guidelines.^3^ ^5^ ^26^ This approach was pragmatic given changing threshold values for the general population and for clinical subgroups during the study period.^3^ ^5^ ^20^ ^26^ ^27^ ^28^ We tested the appropriateness of this approach with two sensitivity analyses, using a ≥160/90 mm Hg threshold for defining hypertension between 1995 and 2000 and a ≥150/90 mm Hg threshold for defining hypertension for those aged 80 or more years, both of which indicated broadly similar trends to the main analysis. We did not require “optimal” doses of each antihypertensive drug because this feature of prescribing is highly individualised, especially in patients who use polypharmacy. Rather, we focused on establishing population trends using population level data.

### Relation to other studies and key differences

One previous study, using three cross sections of the American National Health and Nutritional Examination Survey examined prevalence of resistant hypertension over time.^29^ This study reported a linear increase in the prevalence of resistant hypertension among all patients with hypertension (treated and untreated), ranging from 5.5% in 1988-94 to 8.5% in 1999-2004 to 11.8% in 2005-08. This steady incline in prevalence mirrors the incline we noted during similar timeframes, although we observed a peak prevalence of almost 8% in 2007 and a plateau and decline thereafter. Our estimates are likely lower because we accounted for adherence in our case definition, whereas this was not possible in the American study. The same reason is plausibly responsible for the difference between a summary prevalence estimate of 14% based on 20 observational studies in a recent systematic review and our peak prevalence estimate of 8%.^7^ Many of the studies included in the review did not account for adherence. In our sensitivity analysis, which did not account for adherence, we found a prevalence of approximately 14% in the past 10 years (see supplementary material 6), thus agreeing with the results reported in the systematic review and recent similar additions to the literature from Africa^11^ and Germany.^12^ By contrast, our results for prevalent resistant hypertension (accommodating for adherence) agree well with prevalence estimates from the 2011 health survey for England.^30^

We found some evidence showing that those aged 80 or more years were more likely to have prevalent resistant hypertension over the study period compared with those aged 65-69 years, a finding that was robust to sensitivity analyses. This adds to evidence from Sweden in which older patients had a higher prevalence of resistant hypertension than their younger counterparts according to various definitions of resistant hypertension.^13^

Data from the Anglo Scandinavian Cardiac Outcomes Trial (ASCOT) estimated incidence of resistant hypertension at approximately 34%, which is a probable overestimate owing to non-representativeness of the clinical trial population.^31^ In contrast with ASCOT data, an observational study found an incidence of approximately 2% based on American administrative claims data between 2002 and 2006, which is more similar to the peak incidence of just over two cases per 100 person years we found in 2004.^6^

### Meaning of findings and possible mechanisms

The explanation for the trends we found is likely multifactorial, relating to detection, treatment, and patient awareness of hypertension as opposed to changing pathology. The steep increase in incidence between 1996 and 2004 might be explained by increased detection and treatment of hypertension during this period, resulting from a burgeoning interest in this clinical area and the publication of several seminal clinical trials.^32^ ^33^ ^34^ ^35^ ^36^ Increasingly aggressive treatment was supported by evidence from the Antihypertensive and Lipid-Lowering Treatment to Prevent Heart Attack Trial (ALLHAT) that most patients needed at least two antihypertensive drugs to achieve target blood pressure levels.^37^ The initiation of the Quality and Outcomes Framework in the UK may partially explain the fall-off in incidence after 2004. This pay for performance scheme incentivised general practitioners to more closely monitor blood pressure levels and to increase the percentage of patients with controlled (<150/90 mm Hg) hypertension. A report on the impact of the framework using QRESEARCH data from primary care found that rates of blood pressure control (<150/90 mm Hg) increased by 65% between 2001 and 2006.^38^ Control rates defined as blood pressure <140/90 mm Hg increased from 5.7% in 2003 to 9.9% in 2014.^39^ Throughout the study period, the proportion of patients aware of their hypertension, and possibly the importance of its treatment, also increased,^30^ perhaps also playing a contributory role in improved control rates.

### Conclusions and implications for clinicians and policy makers

Resistant hypertension is common in the UK hypertensive population. Given the importance of hypertension as a modifiable risk factor for cardiovascular disease,^40^ continued efforts are warranted to reduce the proportion of the population with uncontrolled hypertension. Points for intervention include frequent checks on drug adherence, considering its role in causation of resistant hypertension, and review of drug regimens.^3^ Where possible, once daily dosing should be used, along with fixed dose combination preparations to help improve adherence.^41^ ^42^ Self management and shared management between general practitioners and other health professionals such as nurses and pharmacists can also help improve blood pressure control.^43^ ^44^ Educational interventions targeted at patients may help to improve awareness and encourage adherence.^45^ Our results are generalisable within the UK and also bear similarities to estimates from various other countries. However, differences in the organisation of care and the implementation of policy initiatives likely lead to a unique pattern of trends in the UK.

**What is already known on this topic**

Uncontrolled hypertension is a leading risk factor for morbidity and mortality from cardiovascular disease and stroke
Those with resistant hypertension (uncontrolled hypertension while treated with three antihypertensives inclusive of a diuretic, or those using four antihypertensives) have a higher risk of cardiovascular events than those without resistant hypertension
Current estimates for the epidemiological burden of resistant hypertension are limited by methodological challenges

**What this study adds**

Incidence of resistant hypertension in more than 1.3 million UK primary care patients with hypertension increased from one case per 100 person years in 1996 to a peak of two cases per 100 person years in 2004; thereafter, the incidence decreased to 0.4 cases per 100 person years in 2015
Prevalence increased from 1.75% in 1995 to a peak of 7.76% in 2007 then plateaued and declined to 6.46% in 2015, reflecting an earlier decline in incidence
Resistant hypertension is common in the UK. Continued effort is needed to reduce the proportion of the population with uncontrolled hypertension given its intrinsic role in the development of cardiovascular disease

## Web extra

Extra material supplied by authors

## Notes

Ethical approval: The study protocol was approved by the London School of Hygiene and Tropical Medicine ethics committee (No 13988) and the independent scientific advisory committee for Medicines and Healthcare products Regulatory Agency (No 16_205). The protocol was made available to reviewers as supplementary material.

Data sharing: No additional data available.

Transparency: The manuscript’s guarantor (SJS) affirms that the manuscript is an honest, accurate, and transparent account of the study being reported; that no important aspects of the study have been omitted; and that any discrepancies from the study as planned have been explained.
