# Supplementary material for: Trends for prevalence and incidence of resistant hypertension: population based cohort study in the UK 1995-2015
Source: BMJ. 2017 Sep 22;358:j3984. doi: 10.1136/bmj.j3984 (PMC5609092; doi:10.1136/bmj.j3984)
Supplement: Supplementary file 1 — Supplementary material: supplementary material 1 and 2 [file sins038885.ww1.pdf]

# Supplementary Material 1

*Description*: Product codes for antihypertensive drugs

| Product Name | Product code |
|---|---|
| bendroflumethiazide 2.5mg tablets | 2 |
| atenolol 50mg tablets | 5 |
| furosemide 40mg tablets | 6 |
| atenolol 100mg tablets | 24 |
| atenolol 25mg tablets | 26 |
| amlodipine besilate 5mg tablets | 29 |
| furosemide 20mg tablets | 55 |
| co-amilofruse 5mg/40mg tablets | 56 |
| bendroflumethiazide 5mg tablets | 58 |
| lisinopril 10mg tablets | 65 |
| lisinopril 20mg tablets | 69 |
| amlodipine besilate 10mg tablets | 71 |
| lisinopril 5mg tablets | 78 |
| ramipril 5mg capsules | 80 |
| ramipril 10mg capsules | 82 |
| perindopril erbumine 4mg tablets | 97 |
| doxazosin 1mg tablets | 119 |
| ramipril 1.25mg capsules | 147 |
| co-amilofruse 2.5mg/20mg tablets | 193 |
| enalapril 5mg tablets | 196 |
| frumil 40mg+5mg tablet (helios healthcare ltd) | 211 |
| diltiazem 120mg modified-release tablets | 219 |
| nifedipine 5mg capsules | 269 |
| lisinopril 2.5mg tablets | 277 |
| propranolol 10mg tablets | 297 |
| clonidine 25microgram tablets | 338 |
| moduretic tablet (bristol-myers squibb pharmaceuticals ltd) | 348 |
| nifedipine 10mg modified-release tablets | 410 |
| enalapril 2.5mg tablets | 448 |
| nifedipine 10mg capsules | 452 |
| bisoprolol 5mg tablets | 472 |
| felodipine 2.5mg modified-release tablets | 491 |
| doxazosin 2mg tablets | 493 |
| felodipine 5mg modified-release tablets | 501 |
| adizem sr 120mg modified-release capsule (napp pharmaceuticals ltd) | 517 |
| losartan 25mg tablets | 520 |
| candesartan 2mg tablets | 529 |
| candesartan 4mg tablets | 531 |
| tildiem la 200mg modified-release capsule (sanofi) | 536 |
| adalat la 20 tablets (bayer plc) | 541 |
| hydrochlorothiazide 25mg tablets | 542 |
| felodipine 10mg modified-release tablets | 568 |
| hydralazine 25mg tablets | 573 |
| valsartan 40mg capsules | 575 |
| atenolol 50mg with chlortalidone 12.5mg tablets | 581 |
| doxazosin 4mg modified-release tablets | 582 |
| prazosin 1mg tablets | 591 |
| perindopril erbumine 2mg tablets | 593 |
| bisoprolol 2.5mg tablets | 594 |
| bisoprolol 1.25mg tablets | 599 |

| | |
|---|---|
| chlortalidone 50mg tablets | 605 |
| losartan 100mg tablets | 624 |
| fosinopril 10mg tablets | 633 |
| diltiazem 60mg modified-release capsules | 636 |
| ramipril 2.5/5mg/10mg capsule | 654 |
| adalat 5mg capsules (bayer plc) | 662 |
| spironolactone 25mg tablets | 692 |
| vera-til sr 120mg tablets (tillomed laboratories ltd) | 700 |
| propranolol 40mg tablets | 707 |
| spironolactone 50mg tablets | 708 |
| ramipril 2.5mg capsules | 709 |
| prazosin 2mg tablets | 726 |
| amlodipine maleate 5mg tablets | 729 |
| nifedipine 20mg modified-release capsules | 737 |
| metoprolol 50mg tablets | 739 |
| amlodipine 5mg tablets | 749 |
| nebivolol 5mg tablets | 751 |
| metoprolol 100mg tablets | 753 |
| cardura xl 4mg tablets (pfizer ltd) | 755 |
| ramipril 10mg tablets | 756 |
| ramipril 1.25mg tablets | 761 |
| co-diovan 80mg/12.5mg tablets (novartis pharmaceuticals uk ltd) | 764 |
| propranolol 80mg modified-release capsules | 769 |
| sotalol 40mg tablets | 786 |
| spironolactone 100mg capsule | 787 |
| adizem xl 240mg capsule (napp pharmaceuticals ltd) | 793 |
| bumetanide 1mg tablets | 814 |
| carvedilol 3.125mg tablets | 817 |
| irbesartan 75mg tablets | 828 |
| co-amilozide 5mg/50mg tablets | 923 |
| co-amilozide 2.5mg/25mg tablets | 924 |
| tildiem retard 90mg tablets (sanofi) | 939 |
| propranolol 80mg tablets | 940 |
| half inderal la 80mg capsules (astrazeneca uk ltd) | 1006 |
| innozide 20mg/12.5mg tablets (merck sharp & dohme ltd) | 1021 |
| inderal 80mg tablets (astrazeneca uk ltd) | 1048 |
| inderal 40mg tablets (astrazeneca uk ltd) | 1050 |
| amiloride 5mg tablets | 1060 |
| verapamil 40mg tablets | 1118 |
| verapamil 80mg tablets | 1120 |
| captopril 12.5mg tablets | 1121 |
| tenoretic 100mg/25mg tablets (astrazeneca uk ltd) | 1124 |
| navidrex -k tablet (novartis pharmaceuticals uk ltd) | 1125 |
| viazem xl 300mg capsules (thornton & ross ltd) | 1130 |
| captopril 25mg tablets | 1143 |
| capoten 25mg tablets (bristol-myers squibb pharmaceuticals ltd) | 1144 |
| cyclopenthiazide 500microgram tablets | 1170 |
| neo-naclex 5mg tablets (mercury pharma group ltd) | 1209 |
| bendroflumethiazide 2.5mg / potassium chloride 630mg (potassium 8.4mmol) modified-release tablets | 1211 |
| neo-naclex-k modified-release tablets (mercury pharma group ltd) | 1213 |
| moduret 25 tablets (merck sharp & dohme ltd) | 1251 |
| nifedipine 12 20mg modified-release tablet | 1262 |
| tenoret 50mg/12.5mg tablets (astrazeneca uk ltd) | 1288 |
| tildiem retard 120mg tablets (sanofi) | 1289 |
| bisoprolol 10mg tablets | 1290 |
| hypovase 1mg tablets (pfizer ltd) | 1292 |
| irbesartan 150mg tablets | 1293 |

| | |
|---|---|
| doxazosin 4mg tablets | 1294 |
| labetalol 400mg tablets | 1295 |
| hydralazine 50mg tablets | 1296 |
| aldactide 50 tablets (pfizer ltd) | 1297 |
| verapamil 240mg modified-release tablets | 1298 |
| enalapril 10mg tablets | 1299 |
| nifensar xl 20mg modified-release tablet (rhone-poulenc rorer ltd) | 1300 |
| frumil ls 20mg+2.5mg tablet (helios healthcare ltd) | 1301 |
| oxprenolol 40mg tablets | 1333 |
| oxprenolol 160mg modified-release tablets | 1334 |
| furosemide with amiloride 40mg+5mg tablet | 1369 |
| propranolol 160mg modified-release capsules | 1448 |
| nifedipine 24 30mg modified-release tablet | 1449 |
| prazosin 500microgram tablets | 1455 |
| capozide 25mg/50mg tablets (bristol-myers squibb pharmaceuticals ltd) | 1520 |
| posicor 50mg tablet (roche products ltd) | 1529 |
| diltiazem 60mg tablets | 1538 |
| sotalol 80mg tablets | 1572 |
| verapamil 120mg modified-release capsules | 1574 |
| labetalol 100mg tablets | 1597 |
| beta-adalat modified-release capsules (bayer plc) | 1684 |
| diltiazem 90mg modified-release capsules | 1686 |
| methyldopa 250mg tablets | 1707 |
| dyazide 50mg/25mg tablets (amco) | 1721 |
| verapamil 120mg tablets | 1747 |
| cordilox 120mg tablets (ivax pharmaceuticals uk ltd) | 1748 |
| burinex k modified-release tablets (leo pharma) | 1776 |
| losartan 50mg tablets | 1780 |
| atenolol 100mg with chlortalidone 25mg tablets | 1788 |
| captopril 50mg tablets | 1807 |
| diltiazem 60mg modified-release tablets | 1836 |
| adalat la 30mg tablet (bayer plc) | 1854 |
| enalapril 20mg tablets | 1904 |
| diltiazem 12hr 120mg modified-release capsules | 1995 |
| aldactide 25 tablets (pfizer ltd) | 2001 |
| amiloride 5mg / hydrochlorothiazide 50mg tablets | 2002 |
| navidrex 500microgram tablets (amco) | 2046 |
| rauwiloid 2mg tablet (3m health care ltd) | 2104 |
| doralese tiltab 20mg tablets (chemidex pharma ltd) | 2117 |
| spironolactone 100mg tablets | 2142 |
| triamterene 50mg capsules | 2179 |
| navispare 2.5mg/250microgram tablets (amco) | 2255 |
| adalat retard 10mg tablets (bayer plc) | 2280 |
| adalat retard 20mg tablets (bayer plc) | 2343 |
| hytrin 5mg tablet (abbott laboratories ltd) | 2346 |
| hytrin 10mg tablet (abbott laboratories ltd) | 2347 |
| hytrin bph 10mg tablet (amdipharm plc) | 2348 |
| trasicor 80mg tablet (novartis pharmaceuticals uk ltd) | 2361 |
| apresoline 25mg tablets (amco) | 2362 |
| aldactone 25mg tablets (pfizer ltd) | 2389 |
| inderal 10mg tablets (astrazeneca uk ltd) | 2414 |
| tenormin ls 50mg tablets (astrazeneca uk ltd) | 2432 |
| diltiazem 60mg modified-release capsules | 2453 |
| burinex a 5mg/1mg tablets (leo pharma) | 2493 |
| bumetanide with amiloride tablets | 2495 |
| nadolol 80mg tablets | 2499 |
| adalat 10mg capsules (bayer plc) | 2521 |
| slozem 120mg capsules (merck serono ltd) | 2528 |

| | |
|---|---|
| tenormin 100mg tablets (astrazeneca uk ltd) | 2587 |
| tenormin 25mg tablets (astrazeneca uk ltd) | 2590 |
| viazem xl 120mg capsules (thornton & ross ltd) | 2592 |
| nifedipine 10mg modified-release capsules | 2605 |
| indapamide 2.5mg tablets | 2612 |
| carvedilol 12.5mg tablets | 2629 |
| dixarit 25microgram tablets (boehringer ingelheim ltd) | 2630 |
| diltiazem 240mg modified-release capsules | 2663 |
| apresoline 50mg tablet (sovereign medical ltd) | 2680 |
| dilzem xl mr 240mg modified-release capsule (elan pharma) | 2686 |
| coracten sr 10mg capsules (ucb pharma ltd) | 2746 |
| lasoride 5mg/40mg tablets (sanofi) | 2772 |
| labetalol 200mg tablets | 2775 |
| oxprenolol 80mg tablets | 2780 |
| burinex 1mg tablets (leo pharma) | 2788 |
| adizem sr 180mg modified-release capsule (napp pharmaceuticals ltd) | 2811 |
| indoramin 20mg tablets | 2816 |
| cyclopenthiazide -k tablets | 2833 |
| clonidine 100microgram tablets | 2878 |
| tildiem 60mg modified-release tablets (sanofi) | 2888 |
| nicardipine 20mg capsules | 2926 |
| frusene 50mg/40mg tablets (orion pharma (uk) ltd) | 2961 |
| minoxidil 5mg tablets | 2967 |
| minoxidil 10mg tablets | 2968 |
| minoxidil 2.5mg tablets | 2970 |
| irbesartan 300mg tablets | 2971 |
| centyl k tablet (edwin burgess ltd) | 2979 |
| zestoretic 20- 20mg+12.5mg tablet (astrazeneca uk ltd) | 2982 |
| inderal la 160mg capsules (astrazeneca uk ltd) | 3005 |
| methyldopa 125mg tablets | 3049 |
| furosemide with triamterene 40mgwith50mg tablet | 3050 |
| hygroton 100mg tablet (alliance pharmaceuticals ltd) | 3054 |
| natrilix sr 1.5mg tablets (servier laboratories ltd) | 3056 |
| securon 120mg tablets (abbott laboratories ltd) | 3057 |
| diltiazem 12hr 180mg modified-release capsules | 3061 |
| acepril 25mg tablets (bristol-myers squibb pharmaceuticals ltd) | 3069 |
| methyldopa 500mg tablets | 3070 |
| adizem sr 90mg modified-release capsule (napp pharmaceuticals ltd) | 3118 |
| propranolol 160mg tablets | 3167 |
| capozide ls tablet (e r squibb and sons ltd) | 3203 |
| lacidipine 4mg tablets | 3221 |
| valsartan 80mg capsules | 3222 |
| furosemide 500mg tablets | 3248 |
| cardene sr 30mg capsules (astellas pharma ltd) | 3302 |
| capoten 12.5mg tablets (bristol-myers squibb pharmaceuticals ltd) | 3310 |
| securon sr 240mg tablets (bgp products ltd) | 3342 |
| half securon sr 120mg tablets (bgp products ltd) | 3343 |
| betaloc 100mg tablets (astrazeneca uk ltd) | 3344 |
| dilzem xl mr 120mg modified-release capsule (elan pharma) | 3370 |
| terazosin 1mg tablets | 3470 |
| betaloc-sa 200mg tablets (astrazeneca uk ltd) | 3474 |
| oxprenolol 20mg tablets | 3516 |
| hydrochlorothiazide 50mg tablets | 3517 |
| amiloride with atenolol with hydrochlorothiazide capsules | 3526 |
| chlortalidone 100mg tablets | 3548 |
| monocor 5mg tablets (wyeth pharmaceuticals) | 3588 |
| dilzem xl mr 180mg modified-release capsule (elan pharma) | 3676 |
| sotalol 160mg with hydrochlorothiazide 25mg tablet | 3691 |

| | |
|---|---|
| amiloride 2.5mg / hydrochlorothiazide 25mg tablets | 3701 |
| adipine mr 20 tablets (chiesi ltd) | 3711 |
| coracten xl 30mg capsules (ucb pharma ltd) | 3712 |
| prazosin 5mg tablets | 3715 |
| zestril 2.5mg tablets (astrazeneca uk ltd) | 3720 |
| oxprenolol 160mg tablet | 3748 |
| co-amilofruse 10mg/80mg tablets | 3793 |
| propanix 40mg tablet (ashbourne pharmaceuticals ltd) | 3827 |
| capoten 50mg tablets (bristol-myers squibb pharmaceuticals ltd) | 3839 |
| istin 5mg tablets (pfizer ltd) | 3917 |
| terazosin 5mg tablets | 3924 |
| quinapril 10mg tablets | 3929 |
| nifedipine 60mg modified-release tablets | 3930 |
| posicor 100mg tablet (roche products ltd) | 3931 |
| verapamil 240mg modified-release capsules | 3943 |
| hygroton 50mg tablets (alliance pharmaceuticals ltd) | 3997 |
| sotacor 80mg tablets (bristol-myers squibb pharmaceuticals ltd) | 4004 |
| slow-trasicor 160mg tablets (amco) | 4025 |
| diurexan 20mg tablets (meda pharmaceuticals ltd) | 4044 |
| dytac 50mg capsules (amco) | 4068 |
| trandolapril 1mg capsules | 4103 |
| hypovase 500microgram tablets (pfizer ltd) | 4111 |
| amias 2mg tablets (takeda uk ltd) | 4155 |
| spiroctan 25mg tablet (roche products ltd) | 4161 |
| furosemide with amiloride 20mg+2.5mg tablet | 4211 |
| catapres 100microgram tablets (boehringer ingelheim ltd) | 4215 |
| cozaar 25mg tablets (merck sharp & dohme ltd) | 4226 |
| adalat la 60mg tablet (bayer plc) | 4227 |
| adipine mr 10 tablets (chiesi ltd) | 4239 |
| celectol 200mg tablet (pantheon healthcare ltd) | 4265 |
| dilzem sr 90mg capsule (elan pharma) | 4308 |
| metolazone 5mg tablets | 4332 |
| metolazone 500microgram low dose tablet | 4334 |
| debrisoquine 20mg tablets | 4374 |
| decaserpyl plus tablet (roussel laboratories ltd) | 4406 |
| slozem 240mg capsules (merck serono ltd) | 4408 |
| carvedilol 6.25mg tablets | 4410 |
| trasidrex modified-release tablets (mercury pharma group ltd) | 4429 |
| cardura 1mg tablets (pfizer ltd) | 4449 |
| cozaar-comp 50mg/12.5mg tablets (merck sharp & dohme ltd) | 4540 |
| atenolol 50mg / nifedipine 20mg modified-release capsules | 4542 |
| staril 10mg tablets (bristol-myers squibb pharmaceuticals ltd) | 4571 |
| visken 5mg tablet (sovereign medical ltd) | 4588 |
| moducren tablets (merck sharp & dohme ltd) | 4605 |
| diltiazem 200mg modified-release capsules | 4635 |
| terazosin 2mg tablets | 4637 |
| valsartan 160mg capsules | 4645 |
| spironolactone 50mg / furosemide 20mg capsules | 4661 |
| amias 4mg tablets (takeda uk ltd) | 4685 |
| labetalol 50mg tablets | 4725 |
| diltiazem 90mg modified-release tablets | 4732 |
| candesartan 16mg tablets | 4741 |
| emcor ls 5mg tablets (merck serono ltd) | 4771 |
| inderetic 80mg/2.5mg capsules (astrazeneca uk ltd) | 4796 |
| cardura 2mg tablets (pfizer ltd) | 4802 |
| diltiazem 240mg modified-release capsules | 4808 |
| candesartan 8mg tablets | 4818 |
| adizem sr 120mg modified-release tablet (napp pharmaceuticals ltd) | 4852 |

| | |
|---|---|
| coracten sr 20mg capsules (ucb pharma ltd) | 4856 |
| fru-co 5mg/40mg tablets (teva uk ltd) | 4873 |
| terazosin 10mg tablets | 4875 |
| diltiazem 24hr 180mg modified-release capsules | 4923 |
| coracten xl 60mg capsules (ucb pharma ltd) | 4939 |
| aldactone 50mg tablets (pfizer ltd) | 4960 |
| atenolol with amiloride and hydrochlorothiazide capsules | 4983 |
| moxonidine 200microgram tablets | 4993 |
| amias 8mg tablets (takeda uk ltd) | 5013 |
| trandolapril 2mg capsules | 5047 |
| angitil sr 180 capsules (chiesi ltd) | 5054 |
| indapamide 1.5mg modified-release tablets | 5112 |
| amias 16mg tablets (takeda uk ltd) | 5117 |
| lacidipine 2mg tablets | 5158 |
| quinapril 20mg tablets | 5159 |
| nifedipine 30mg modified-release capsules | 5162 |
| angiopine mr 20mg tablets (ashbourne pharmaceuticals ltd) | 5181 |
| hypovase 2mg tablets (pfizer ltd) | 5183 |
| enalapril 20mg / hydrochlorothiazide 12.5mg tablets | 5189 |
| dilzem sr 120mg capsule (elan pharma) | 5194 |
| furosemide with amiloride 80mg+10mg tablet | 5220 |
| slozem 180mg capsules (merck serono ltd) | 5234 |
| tritace 2.5mg capsules (sanofi) | 5275 |
| fortipine la 40 tablets (amco) | 5277 |
| pindolol 5mg tablets | 5284 |
| clonidine 250microgram modified-release capsules | 5289 |
| tildiem la 300mg modified-release capsule (sanofi) | 5296 |
| diltiazem 24hr 300mg modified-release capsules | 5326 |
| corgaretic 40mg tablets (sanofi-synthelabo ltd) | 5330 |
| hytrin bph 5mg tablet (amdipharm plc) | 5337 |
| diltiazem 300mg modified-release capsules | 5348 |
| co-triamterzide 50mg/25mg tablets | 5416 |
| nicardipine 30mg modified-release capsules | 5477 |
| doxazosin 8mg modified-release tablets | 5496 |
| dilzem sr 60mg capsule (elan pharma) | 5513 |
| zanidip 10mg tablets (recordati pharmaceuticals ltd) | 5570 |
| lercanidipine 10mg tablets | 5593 |
| coversyl 2mg tablets (servier laboratories ltd) | 5612 |
| cardura xl 8mg tablets (pfizer ltd) | 5618 |
| bisoprolol 7.5mg tablets | 5713 |
| co-tenidone 100mg/25mg tablets | 5721 |
| cozaar 50mg tablets (merck sharp & dohme ltd) | 5723 |
| amiloride 2.5mg / cyclopenthiazide 250microgram tablets | 5727 |
| tritace 5mg capsules (sanofi) | 5735 |
| coversyl 4mg tablets (servier laboratories ltd) | 5800 |
| tensipine mr 20 tablets (genus pharmaceuticals ltd) | 5806 |
| indoramin 25mg tablets | 5815 |
| beta-cardone 40mg tablets (focus pharmaceuticals ltd) | 5858 |
| fosinopril 20mg tablets | 5861 |
| istin 10mg tablets (pfizer ltd) | 5914 |
| monocor 10mg tablets (wyeth pharmaceuticals) | 5968 |
| telmisartan 40mg tablets | 5988 |
| perindopril erbumine 8mg tablets | 6078 |
| bumetanide 500microgram / potassium chloride 573mg (potassium 7.7mmol) modified-release tablets | 6160 |
| tadalafil 20mg tablets | 6207 |
| olmesartan medoxomil 10mg tablets | 6217 |
| telmisartan 20mg tablets | 6243 |

| | |
|---|---|
| tritace 1.25mg tablets (sanofi) | 6261 |
| olmesartan medoxomil 20mg tablets | 6285 |
| ramipril 5mg tablets | 6288 |
| adizem xl 300mg capsule (napp pharmaceuticals ltd) | 6309 |
| ramipril 2.5mg tablets | 6314 |
| olmesartan medoxomil 40mg tablets | 6351 |
| zestoretic 10- 10mg+12.5mg tablet (astrazeneca uk ltd) | 6359 |
| tritace 5mg tablets (sanofi) | 6362 |
| tritace 2.5mg tablets (sanofi) | 6364 |
| tanatril 5mg tablets (chiesi ltd) | 6408 |
| losartan 50mg / hydrochlorothiazide 12.5mg tablets | 6437 |
| lisinopril 20mg / hydrochlorothiazide 12.5mg tablets | 6468 |
| amlodipine maleate 10mg tablets | 6477 |
| univer 120mg modified-release capsules (teva uk ltd) | 6510 |
| diovan 160mg capsules (novartis pharmaceuticals uk ltd) | 6518 |
| clonidine 300microgram tablets | 6694 |
| beta-cardone 80mg tablets (focus pharmaceuticals ltd) | 6751 |
| quinapril 5mg tablets | 6765 |
| lisinopril 10mg / hydrochlorothiazide 12.5mg tablets | 6786 |
| perindopril erbumine 4mg / indapamide 1.25mg tablets | 6794 |
| zestril 10mg tablets (astrazeneca uk ltd) | 6806 |
| zestril 5mg tablets (astrazeneca uk ltd) | 6807 |
| amlodipine 10mg tablets | 6856 |
| co-diovan 160mg/12.5mg tablets (novartis pharmaceuticals uk ltd) | 6877 |
| eprosartan 300mg tablets | 6939 |
| candesartan 32mg tablets | 7043 |
| carvedilol 25mg tablets | 7049 |
| metoprolol 100mg / hydrochlorothiazide 12.5mg tablets | 7066 |
| bisoprolol 3.75mg tablets | 7091 |
| dytide capsules (mercury pharma group ltd) | 7136 |
| moxonidine 400microgram tablets | 7174 |
| plendil 10mg modified-release tablets (astrazeneca uk ltd) | 7280 |
| accupro 5mg tablets (pfizer ltd) | 7314 |
| aprovel 75mg tablets (sanofi) | 7338 |
| viazem xl 360mg capsules (thornton & ross ltd) | 7398 |
| trandolapril 500microgram capsules | 7419 |
| lasilactone 20mg/50mg capsules (sanofi) | 7441 |
| trasicor 20mg tablet (novartis pharmaceuticals uk ltd) | 7474 |
| nebilet 5mg tablets (a. menarini farmaceutica internazionale srl) | 7528 |
| nifopress retard 20mg tablets (amco) | 7541 |
| kalten capsules (m & a pharmachem ltd) | 7543 |
| doxadura 2mg tablets (discovery pharmaceuticals ltd) | 7547 |
| doxadura 1mg tablets (discovery pharmaceuticals ltd) | 7549 |
| cardene 30mg capsules (astellas pharma ltd) | 7562 |
| lasikal modified-release tablets (borg medicare) | 7582 |
| lasix 40mg tablets (sanofi) | 7606 |
| xipamide 20mg tablets | 7618 |
| acebutolol 400mg tablets | 7620 |
| aldomet 250mg tablet (merck sharp & dohme ltd) | 7626 |
| natrilix 2.5mg tablets (servier laboratories ltd) | 7641 |
| aldomet 500mg tablet (merck sharp & dohme ltd) | 7642 |
| synadrin 60mg tablet (hoechst uk ltd) | 7681 |
| aprinox 5mg tablets (amdipharm plc) | 7698 |
| arelix 6mg capsule (hoechst marion roussel) | 7709 |
| diumide-k continus tablets (teofarma) | 7734 |
| triamterene 50mg / benzthiazide 25mg capsules | 7740 |
| lasix 20mg tablets (borg medicare) | 7799 |
| bumetanide 5mg tablets | 7806 |

| | |
|---|---|
| blocadren 10mg tablet (merck sharp & dohme ltd) | 7852 |
| timolol 10mg tablets | 7853 |
| declinax 10mg tablet (roche products ltd) | 7911 |
| ismelin 10mg tablet (sovereign medical ltd) | 7922 |
| guanethidine 10mg tablet | 7923 |
| aldactone 100mg tablets (pfizer ltd) | 7952 |
| spironolactone 50mg with hydroflumethiazide 50mg tablet | 7961 |
| celiprolol 400mg tablets | 7974 |
| spiroctan 100mg capsule (roche products ltd) | 7991 |
| sectral 400mg tablets (sanofi) | 8023 |
| gopten 1mg capsules (abbott laboratories ltd) | 8025 |
| gopten 2mg capsules (abbott laboratories ltd) | 8026 |
| aldomet 125mg tablet (merck sharp & dohme ltd) | 8033 |
| torasemide 5mg tablets | 8052 |
| normetic tablet (abbott laboratories ltd) | 8058 |
| sotalol 80mg with hydrochlorothiazide 12.5mg tablet | 8061 |
| metoprolol 200mg modified-release tablets | 8068 |
| betaloc 50mg tablets (astrazeneca uk ltd) | 8071 |
| hytrin 2mg tablet (abbott laboratories ltd) | 8077 |
| cardura 4mg tablet (pfizer ltd) | 8086 |
| furosemide 40mg / potassium chloride 600mg (potassium 8mmol) modified-release tablets | 8102 |
| innovace 20mg tablets (merck sharp & dohme ltd) | 8105 |
| innovace 2.5mg tablets (merck sharp & dohme ltd) | 8106 |
| acebutolol 200mg capsules | 8113 |
| lopresoretic tablet (novartis pharmaceuticals uk ltd) | 8147 |
| acebutolol 100mg capsules | 8172 |
| secadrex 200mg/12.5mg tablets (sanofi) | 8189 |
| hypovase 5mg tablet (pfizer ltd) | 8198 |
| nicardipine 30mg capsules | 8201 |
| nifedipine 24 20mg modified-release tablet | 8213 |
| prescal 2.5mg tablets (novartis pharmaceuticals uk ltd) | 8257 |
| celiprolol 200mg tablets | 8262 |
| zestril 20mg tablets (astrazeneca uk ltd) | 8268 |
| trasicor 40mg tablet (novartis pharmaceuticals uk ltd) | 8290 |
| catapres pl perlongets 250microgram capsules (boehringer ingelheim ltd) | 8296 |
| isradipine 2.5mg tablets | 8310 |
| inderal 160mg tablet (astrazeneca uk ltd) | 8331 |
| declinax 20mg tablet (roche products ltd) | 8342 |
| inderex 160mg/5mg modified-release capsules (astrazeneca uk ltd) | 8369 |
| spironolactone 25mg with hydroflumethiazide 25mg tablet | 8521 |
| securon 40mg tablet (abbott laboratories ltd) | 8524 |
| aprinox 2.5mg tablets (amco) | 8526 |
| sectral 200mg capsules (sanofi) | 8555 |
| adizem xl 120mg capsule (napp pharmaceuticals ltd) | 8558 |
| metenix 5mg tablets (sanofi) | 8602 |
| prestim tablet (icn pharmaceuticals france s.a.) | 8623 |
| tenif 50mg/20mg modified-release capsules (astrazeneca uk ltd) | 8642 |
| oxprenolol with cyclopenthiazide 160mg+0.25mg modified-release tablet | 8673 |
| trandate 200mg tablets (focus pharmaceuticals ltd) | 8707 |
| verapamil hcl 120mg modified release tablets | 8759 |
| innovace 5mg tablets (merck sharp & dohme ltd) | 8800 |
| trandate 400mg tablets (focus pharmaceuticals ltd) | 8807 |
| innovace 10mg tablets (merck sharp & dohme ltd) | 8830 |
| chlorothiazide 500mg tablets | 8836 |
| hypovase benign prostatic hyperplasia 1mg tablet (pfizer ltd) | 8863 |
| cordilox 40mg tablets (ivax pharmaceuticals uk ltd) | 8884 |
| hygroton -k tablet (novartis pharmaceuticals uk ltd) | 8891 |
| triam-co 50mg/25mg tablets (ivax pharmaceuticals uk ltd) | 8897 |

| | |
|---|---|
| nadolol 40mg tablets | 8935 |
| univer 240mg modified-release capsules (teva uk ltd) | 8945 |
| verapamil 180mg modified-release capsules | 8975 |
| propanix 160mg modified-release capsule (ashbourne pharmaceuticals ltd) | 8978 |
| propranolol 160mg modified-release / bendroflumethiazide 5mg capsules | 8987 |
| trandate 100mg tablets (focus pharmaceuticals ltd) | 9016 |
| indoramin 50mg tablet | 9019 |
| viskaldix tablets (amco) | 9143 |
| atenolol 25mg / bendroflumethiazide 1.25mg capsules | 9178 |
| aprovel 150mg tablets (sanofi) | 9196 |
| triamterene with hydrochlorothiazide 50mg + 25mg tablet | 9223 |
| methyldopa 250mg capsule | 9225 |
| adizem xl 180mg capsule (napp pharmaceuticals ltd) | 9240 |
| nifedipine 40mg modified-release tablets | 9269 |
| trandate 50mg tablets (focus pharmaceuticals ltd) | 9273 |
| sotalol 160mg tablets | 9292 |
| plendil 2.5mg modified-release tablets (astrazeneca uk ltd) | 9334 |
| adizem 60mg modified-release tablet (napp pharmaceuticals ltd) | 9374 |
| nicardipine 45mg modified-release capsules | 9386 |
| angitil sr 120 capsules (chiesi ltd) | 9410 |
| frusemek 40mg+5mg tablet (approved prescription services ltd) | 9431 |
| plendil 5mg modified-release tablets (astrazeneca uk ltd) | 9437 |
| amiloride 5mg / furosemide 40mg tablets | 9456 |
| loniten 5mg tablets (pfizer ltd) | 9463 |
| hypolar retard 20 tablets (sandoz ltd) | 9485 |
| slofedipine xl 60 tablets (zentiva) | 9553 |
| verapamil 120mg modified-release tablets | 9569 |
| slofedipine xl 30mg tablets (zentiva) | 9573 |
| tritace 1.25mg capsules (aventis pharma) | 9646 |
| motens 4mg tablets (glaxosmithkline uk ltd) | 9670 |
| tritace 10mg capsules (sanofi) | 9693 |
| loniten 2.5mg tablets (pfizer ltd) | 9697 |
| diltiazem 24hr 120mg modified-release capsules | 9708 |
| calcicard cr 90mg tablets (teva uk ltd) | 9723 |
| quinapril 40mg tablets | 9731 |
| teveten 300mg tablets (bgp products ltd) | 9745 |
| physiotens 400microgram tablets (bgp products ltd) | 9749 |
| nifedipine 60mg modified-release capsules | 9750 |
| carace 20 tablet (bristol-myers squibb pharmaceuticals ltd) | 9764 |
| co-tenidone 50mg/12.5mg tablets | 9783 |
| physiotens 300microgram tablets (bgp products ltd) | 9876 |
| tritace 10mg tablets (sanofi) | 9915 |
| trandolapril 4mg capsules | 9948 |
| torem 5mg tablets (meda pharmaceuticals ltd) | 10066 |
| doxadura 4mg tablets (discovery pharmaceuticals ltd) | 10088 |
| nifedipress mr 10mg modified-release tablet (sandoz ltd) | 10135 |
| nifedipress mr 20 tablets (dexcel-pharma ltd) | 10136 |
| felendil xl 5mg modified-release tablet (ratiopharm uk ltd) | 10153 |
| atenix 50 tablets (ashbourne pharmaceuticals ltd) | 10191 |
| adipine xl 60mg tablets (chiesi ltd) | 10246 |
| eplerenone 25mg tablets | 10251 |
| moxonidine 300microgram tablets | 10253 |
| adizem-xl 200mg capsules (napp pharmaceuticals ltd) | 10267 |
| coaprovel 150mg/12.5mg tablets (sanofi) | 10316 |
| losartan 100mg / hydrochlorothiazide 25mg tablets | 10323 |
| lasix 500mg tablets (sanofi) | 10392 |
| lopresor 50mg tablet (novartis pharmaceuticals uk ltd) | 10429 |
| nimotop 30mg tablets (bayer plc) | 10595 |

| | |
|---|---|
| co-betaloc tablets (pfizer ltd) | 10627 |
| verapamil 160mg tablets | 10688 |
| decaserpyl 5mg tablet (roussel laboratories ltd) | 10713 |
| methoserpidine 5mg tablet | 10714 |
| corgard 80mg tablets (sanofi) | 10716 |
| trasicor 160mg tablet (novartis pharmaceuticals uk ltd) | 10777 |
| lasix with k tablet (hoechst marion roussel) | 10781 |
| securon 80mg tablet (abbott laboratories ltd) | 10832 |
| bethanidine sulphate 10mg tablets | 10879 |
| carace 2.5mg tablets (bristol-myers squibb pharmaceuticals ltd) | 10882 |
| emcor 10mg tablets (merck serono ltd) | 10892 |
| captopril 50mg with hydrochlorothiazide 25mg tablets | 10902 |
| hydrochlorothiazide with captopril 25mg with 50mg tablet | 11133 |
| spirolone 25mg tablet (berk pharmaceuticals ltd) | 11156 |
| physiotens 200microgram tablets (bgp products ltd) | 11177 |
| angitil sr 90 capsules (chiesi ltd) | 11223 |
| diovan 40mg capsules (novartis pharmaceuticals uk ltd) | 11251 |
| diovan 80mg capsules (novartis pharmaceuticals uk ltd) | 11252 |
| triamterene 50mg / furosemide 40mg tablets | 11265 |
| torem 2.5mg tablets (meda pharmaceuticals ltd) | 11268 |
| bendroflumethiazide 5mg with nadolol 40mg tablets | 11338 |
| aprovel 300mg tablets (sanofi) | 11348 |
| co-zidocapt 25mg/50mg tablets | 11351 |
| sotacor 160mg tablets (bristol-myers squibb pharmaceuticals ltd) | 11380 |
| co-flumactone 50mg/50mg tablets | 11384 |
| baratol 25mg tablet (shire pharmaceuticals ltd) | 11394 |
| irbesartan 150mg / hydrochlorothiazide 12.5mg tablets | 11448 |
| irbesartan 300mg / hydrochlorothiazide 12.5mg tablets | 11469 |
| torasemide 2.5mg tablets | 11487 |
| nifedipress mr 10 tablets (dexcel-pharma ltd) | 11512 |
| coaprovel 300mg/12.5mg tablets (sanofi) | 11526 |
| nimodipine 30mg tablets | 11547 |
| co-zidocapt 12.5mg/25mg tablets | 11561 |
| ramipril 5mg with felodipine 5mg modified-release tablet | 11567 |
| captopril 25mg with hydrochlorothiazide 12.5mg tablets | 11641 |
| calchan mr 20 tablets (ranbaxy (uk) ltd) | 11769 |
| dilzem sr 60 capsules (teva uk ltd) | 11770 |
| valsartan 160mg / hydrochlorothiazide 12.5mg tablets | 11864 |
| cardene 20mg capsules (astellas pharma ltd) | 11943 |
| ramipril 2.5mg with felodipine 2.5mg modified-release tablet | 11965 |
| motens 2mg tablets (glaxosmithkline uk ltd) | 11966 |
| vertab sr 240 tablets (chiesi ltd) | 11972 |
| calcicard cr 120mg tablets (teva uk ltd) | 11973 |
| betim 10mg tablet (icn pharmaceuticals france s.a.) | 12037 |
| propranolol 80mg / bendroflumethiazide 2.5mg capsules | 12054 |
| cordilox 160mg tablets (ivax pharmaceuticals uk ltd) | 12104 |
| hydroflumethiazide 50mg tablet | 12110 |
| burinex 5mg tablets (leo pharma) | 12294 |
| sectral 100mg capsules (sanofi) | 12296 |
| carace 20mg tablets (bristol-myers squibb pharmaceuticals ltd) | 12313 |
| etacrynic 50mg tablets | 12354 |
| nephril 1mg tablet (pfizer ltd) | 12360 |
| piretanide 6mg capsule | 12367 |
| univer 180mg modified-release capsules (teva uk ltd) | 12392 |
| cilazapril 500microgram tablets | 12411 |
| cilazapril 2.5mg tablets | 12412 |
| sotazide tablet (bristol-myers squibb pharmaceuticals ltd) | 12456 |
| berkolol 10mg tablet (berk pharmaceuticals ltd) | 12495 |

| | |
|---|---|
| timolol maleate with bendroflumethiazide 20mg + 5mg tablet | 12517 |
| kalspare tablet (dominion pharma) | 12546 |
| triamterene 50mg / chlortalidone 50mg tablets | 12547 |
| cilazapril 1mg tablets | 12574 |
| nifelease 20mg modified-release tablet (eastern pharmaceuticals ltd) | 12606 |
| unipine xl 30mg modified-release tablet (genus pharmaceuticals ltd) | 12613 |
| diltiazem hcl 90mg modified-release tablet (actavis uk ltd) | 12639 |
| timolol 10mg / bendroflumethiazide 2.5mg tablets | 12651 |
| angiozem cr 90mg tablets (ashbourne pharmaceuticals ltd) | 12705 |
| tanatril 10mg tablets (chiesi ltd) | 12815 |
| eprosartan 600mg tablets | 12836 |
| imidapril 10mg tablets | 12858 |
| telmisartan 80mg tablets | 12874 |
| cardene sr 45mg capsules (astellas pharma ltd) | 12875 |
| cilazapril 5mg tablets | 13026 |
| viazem xl 240mg capsules (thornton & ross ltd) | 13027 |
| angitil xl 240 capsules (chiesi ltd) | 13033 |
| sotalol 200mg tablets | 13051 |
| dilzem xl 180 capsules (teva uk ltd) | 13075 |
| eprosartan 400mg tablets | 13123 |
| dilzem xl 240 capsules (teva uk ltd) | 13127 |
| adipine xl 30mg tablets (chiesi ltd) | 13139 |
| dilzem xl 120 capsules (teva uk ltd) | 13240 |
| lercanidipine 20mg tablets | 13243 |
| vera-til sr 240mg tablets (tillomed laboratories ltd) | 13251 |
| dilzem sr 90 capsules (teva uk ltd) | 13302 |
| midamor 5mg tablet (msd thomas morson pharmaceuticals) | 13352 |
| esidrex 50mg tablet (novartis pharmaceuticals uk ltd) | 13363 |
| ismelin 25mg tablet (sovereign medical ltd) | 13379 |
| angiozem 60mg modified-release tablets (ashbourne pharmaceuticals ltd) | 13410 |
| corgard 40mg tablets (sanofi-synthelabo ltd) | 13415 |
| frumil forte 10mg/80mg tablets (sanofi) | 13435 |
| beta-cardone 200mg tablets (focus pharmaceuticals ltd) | 13487 |
| lopresor 100mg tablet (novartis pharmaceuticals uk ltd) | 13499 |
| hydrenox 50mg tablet (knoll ltd) | 13525 |
| atenix co 100 tablets (ashbourne pharmaceuticals ltd) | 13526 |
| staril 20mg tablets (bristol-myers squibb pharmaceuticals ltd) | 13589 |
| alphavase 2 tablets (ashbourne pharmaceuticals ltd) | 13610 |
| angiopine mr 10mg tablets (ashbourne pharmaceuticals ltd) | 13672 |
| angiopine la 40mg tablet (ashbourne pharmaceuticals ltd) | 13699 |
| micardis 40mg tablets (boehringer ingelheim ltd) | 13821 |
| verapress mr 240mg tablets (actavis uk ltd) | 13856 |
| co-prenozide 160mg/0.25mg modified-release tablets | 13871 |
| diltiazem 360mg modified-release capsules | 13926 |
| cordilox mr 240mg tablets (teva uk ltd) | 13965 |
| cardicor 2.5mg tablets (merck serono ltd) | 14030 |
| pindolol 10mg / clopamide 5mg tablets | 14057 |
| cardicor 1.25mg tablets (merck serono ltd) | 14058 |
| eucardic 3.125mg tablets (roche products ltd) | 14117 |
| acebutolol 200mg / hydrochlorothiazide 12.5mg tablets | 14126 |
| inspra 25mg tablets (pfizer ltd) | 14144 |
| eucardic 6.25mg tablets (roche products ltd) | 14146 |
| coversyl plus tablets (servier laboratories ltd) | 14228 |
| valsartan 160mg / hydrochlorothiazide 25mg tablets | 14283 |
| zanidip 20mg tablets (recordati pharmaceuticals ltd) | 14300 |
| vascalpha 10mg modified-release tablets (actavis uk ltd) | 14305 |
| carace 5mg tablets (bristol-myers squibb pharmaceuticals ltd) | 14387 |
| corgaretic 80mg tablets (sanofi-synthelabo ltd) | 14438 |

| | |
|---|---|
| esbatal 50mg tablet (wellcome medical division) | 14442 |
| accupro 10mg tablets (pfizer ltd) | 14477 |
| accupro 20mg tablets (pfizer ltd) | 14478 |
| loniten 10mg tablets (pfizer ltd) | 14495 |
| propanix 10mg tablet (ashbourne pharmaceuticals ltd) | 14552 |
| amiloride 5mg / bumetanide 1mg tablets | 14587 |
| pindolol 15mg tablets | 14673 |
| hydrochlorothiazide with losartan 12.5mg with 50mg tablet | 14738 |
| frusid 40mg tablets (dr reddy's laboratories (uk) ltd) | 14761 |
| bedranol sr 80mg capsules (sandoz ltd) | 14808 |
| calchan mr 10 tablets (ranbaxy (uk) ltd) | 14861 |
| telmisartan 40mg / hydrochlorothiazide 12.5mg tablets | 14870 |
| valsartan 40mg tablets | 14943 |
| coversyl 8mg tablets (servier laboratories ltd) | 14960 |
| cozaar 100mg tablets (merck sharp & dohme ltd) | 14965 |
| olmetec 10mg tablets (daiichi sankyo uk ltd) | 14983 |
| accuretic 12.5mg/10mg tablets (pfizer ltd) | 15031 |
| tolerzide tablet (bristol-myers squibb pharmaceuticals ltd) | 15042 |
| spiroctan 50mg tablet (roche products ltd) | 15052 |
| accupro 40mg tablets (pfizer ltd) | 15096 |
| quinapril 10mg / hydrochlorothiazide 12.5mg tablets | 15108 |
| nifedipine with atenolol 20mg + 50mg capsule | 15117 |
| moexipril 7.5mg tablets | 15121 |
| hydrochlorothiazide with triamterene 25mgwith50mg tablet | 15127 |
| hydrochlorothiazide with captopril 12.5mg with 25mg tablet | 15135 |
| totamol 50mg tablet (c p pharmaceuticals ltd) | 15176 |
| dilcardia xl 180mg modified-release capsule (generics (uk) ltd) | 15221 |
| angitil xl 300 capsules (chiesi ltd) | 15288 |
| baycaron 25mg tablet (bayer plc) | 15457 |
| metoprolol tartrate with chlortalidone tablet | 15488 |
| reserpine with hydrochlorothiazide tablet | 15493 |
| cilazapril 250micrograms tablets | 15605 |
| half-betadur cr 80mg capsule (monmouth pharmaceuticals ltd) | 15619 |
| mibefradil 50mg tablet | 15652 |
| genalat retard 20mg modified-release tablet (wyeth pharmaceuticals) | 15715 |
| totamol 100mg tablet (c p pharmaceuticals ltd) | 15730 |
| co-flumactone 25mg/25mg tablets | 15811 |
| amiloride 2.5mg / furosemide 20mg tablets | 15874 |
| captopril 2mg tablets | 15958 |
| dilzem sr 120 capsules (teva uk ltd) | 16038 |
| valsartan 80mg / hydrochlorothiazide 12.5mg tablets | 16060 |
| nifedipress mr 10 tablets (teva uk ltd) | 16073 |
| telmisartan 80mg / hydrochlorothiazide 12.5mg tablets | 16161 |
| vascace 5mg tablets (roche products ltd) | 16196 |
| vascace 2.5mg tablets (roche products ltd) | 16197 |
| baratol 50mg tablet (shire pharmaceuticals ltd) | 16198 |
| hytrin bph 2mg tablet (amdipharm plc) | 16201 |
| froop 40mg tablets (ashbourne pharmaceuticals ltd) | 16206 |
| vascace 1mg tablets (roche products ltd) | 16212 |
| teveten 400mg tablets (abbott healthcare products ltd) | 16285 |
| verapress mr 240mg tablets (dexcel-pharma ltd) | 16328 |
| teveten 600mg tablets (bgp products ltd) | 16371 |
| kalspare tablets (dhp healthcare ltd) | 16498 |
| eplerenone 50mg tablets | 16531 |
| labrocol 400mg tablet (lagap) | 16645 |
| cordilox 80mg tablets (ivax pharmaceuticals uk ltd) | 16677 |
| carace 10mg tablets (bristol-myers squibb pharmaceuticals ltd) | 16701 |
| gopten 500microgram capsules (abbott laboratories ltd) | 16710 |

| | |
|---|---|
| celectol 400mg tablet (pantheon healthcare ltd) | 16776 |
| chlortalidone 25mg with atenolol 100mg tablets | 16786 |
| angiozem cr 120mg tablets (ashbourne pharmaceuticals ltd) | 16850 |
| imidapril 5mg tablets | 16924 |
| triapin 5mg/5mg modified-release tablets (sanofi) | 17006 |
| moexipril 15mg tablets | 17120 |
| mefruside 25mg tablet | 17143 |
| monozide 10 tablets (wyeth pharmaceuticals) | 17149 |
| esidrex 25mg tablet (novartis pharmaceuticals uk ltd) | 17252 |
| guanethidine 25mg tablet | 17291 |
| atenix 25 tablets (ashbourne pharmaceuticals ltd) | 17322 |
| cardilate mr 10mg tablets (teva uk ltd) | 17325 |
| nifedotard 20 mr 20mg modified-release tablet (galen ltd) | 17338 |
| nivaten retard 10mg modified-release tablet (actavis uk ltd) | 17342 |
| zemtard 180 xl capsules (galen ltd) | 17406 |
| zemtard 120 xl capsules (galen ltd) | 17425 |
| nifedipress mr 10mg modified-release tablet (sterwin medicines) | 17448 |
| bisoprolol 10mg / hydrochlorothiazide 6.25mg tablets | 17462 |
| felodipine 5mg modified-release / ramipril 5mg tablets | 17474 |
| zemtard 300 xl capsules (galen ltd) | 17492 |
| micardis 80mg tablets (boehringer ingelheim ltd) | 17545 |
| felotens xl 5mg tablets (genus pharmaceuticals ltd) | 17557 |
| bendroflumethiazide 2.5mg / potassium chloride 573mg (potassium 7.7mmol) modified-release tablets | 17561 |
| felotens xl 10mg tablets (genus pharmaceuticals ltd) | 17566 |
| slozem 300mg capsules (merck serono ltd) | 17586 |
| verapress mr 240mg tablets (sandoz ltd) | 17599 |
| cardicor 5mg tablets (merck serono ltd) | 17615 |
| amlostin 5mg tablets (discovery pharmaceuticals ltd) | 17640 |
| carace 10 tablet (bristol-myers squibb pharmaceuticals ltd) | 17655 |
| viazem xl 180mg capsules (thornton & ross ltd) | 17666 |
| micardis 20mg tablets (boehringer ingelheim ltd) | 17686 |
| micardisplus 80mg/12.5mg tablets (boehringer ingelheim ltd) | 17689 |
| saluric 500mg tablet (merck sharp & dohme ltd) | 17720 |
| spiroprop tablet (pharmacia ltd) | 17783 |
| spirolone 100mg tablet (berk pharmaceuticals ltd) | 17902 |
| spirolone 50mg tablet (berk pharmaceuticals ltd) | 17950 |
| furosemide 20mg / potassium chloride 750mg (potassium 10mmol) modified-release tablets | 17960 |
| nisoldipine 20mg modified-release tablets | 18038 |
| torasemide 10mg tablets | 18096 |
| cardicor 7.5mg tablets (merck serono ltd) | 18185 |
| olmesartan medoxomil 20mg / hydrochlorothiazide 12.5mg tablets | 18200 |
| micardisplus 40mg/12.5mg tablets (boehringer ingelheim ltd) | 18202 |
| imidapril 20mg tablets | 18219 |
| trandolapril with verapamil 2mg + 180mg modified-release capsule | 18223 |
| esbatal 10mg tablet (wellcome medical division) | 18247 |
| metalpha 250mg tablet (ashbourne pharmaceuticals ltd) | 18252 |
| acezide 25mg/50mg tablets (bristol-myers squibb pharmaceuticals ltd) | 18263 |
| enduron 5mg tablet (abbott laboratories ltd) | 18267 |
| acepril 12.5mg tablets (bristol-myers squibb pharmaceuticals ltd) | 18269 |
| co-betaloc sa tablets (pfizer ltd) | 18287 |
| acepril 50mg tablets (bristol-myers squibb pharmaceuticals ltd) | 18325 |
| aridil 20mg+2.5mg tablet (c p pharmaceuticals ltd) | 18332 |
| amilmaxco 5mg/50mg tablets (ashbourne pharmaceuticals ltd) | 18361 |
| dilcardia sr 90mg capsules (generics (uk) ltd) | 18379 |
| diltiazem hcl 180mg modified-release capsule (hillcross pharmaceuticals ltd) | 18403 |
| diltiazem 60mg modified-release capsules (a a h pharmaceuticals ltd) | 18404 |
| eucardic 12.5mg tablets (roche products ltd) | 18414 |

| | |
|---|---|
| amiloride 10mg / furosemide 80mg tablets | 18497 |
| diltiazem and hydrochlorothiazide 150mg+12.5mg modified-release capsules | 18606 |
| edecrin 50mg tablet (merck sharp & dohme ltd) | 18650 |
| triamaxco 50mg/25mg tablets (ashbourne pharmaceuticals ltd) | 18726 |
| tenben 25mg/1.25mg capsules (galen ltd) | 18743 |
| disogram sr 90mg capsules (ranbaxy (uk) ltd) | 18830 |
| disogram sr 60mg capsules (ranbaxy (uk) ltd) | 18834 |
| disogram sr 120mg capsules (ranbaxy (uk) ltd) | 18852 |
| disogram sr 180mg capsules (ranbaxy (uk) ltd) | 18874 |
| olmesartan medoxomil 20mg / hydrochlorothiazide 25mg tablets | 18903 |
| olmetec 20mg tablets (daiichi sankyo uk ltd) | 18910 |
| totamol 25mg tablet (c p pharmaceuticals ltd) | 18950 |
| centyl 2.5mg tablet (edwin burgess ltd) | 18973 |
| calcicard 60mg tablet (3m health care ltd) | 18975 |
| clinium 120mg tablet (leo pharma) | 19013 |
| chlortalidone 12.5mg with atenolol 50mg tablets | 19055 |
| syscor mr 10 tablets (forest laboratories uk ltd) | 19129 |
| bendroflumethiazide 2.5mg with timolol maleate 10mg tablets | 19142 |
| tensipine mr 10 tablets (genus pharmaceuticals ltd) | 19170 |
| atenolol 25mg tablets (ivax pharmaceuticals uk ltd) | 19172 |
| verapamil 40mg tablets (ivax pharmaceuticals uk ltd) | 19175 |
| bisoprolol 10mg tablets (ranbaxy (uk) ltd) | 19178 |
| atenolol 50mg tablets (ivax pharmaceuticals uk ltd) | 19182 |
| atenolol 100mg tablets (teva uk ltd) | 19191 |
| furosemide 40mg tablet (m & a pharmachem ltd) | 19192 |
| doxazosin 2mg tablets (teva uk ltd) | 19193 |
| furosemide 20mg tablets (teva uk ltd) | 19194 |
| spironolactone 50mg tablet (wyeth pharmaceuticals) | 19195 |
| lisinopril 20mg tablets (teva uk ltd) | 19198 |
| bisoprolol 5mg tablets (ivax pharmaceuticals uk ltd) | 19200 |
| carvedilol 6.25mg tablets (teva uk ltd) | 19202 |
| lisinopril 5mg tablets (teva uk ltd) | 19204 |
| enalapril 10mg tablets (actavis uk ltd) | 19208 |
| doxazosin 4mg tablets (ivax pharmaceuticals uk ltd) | 19216 |
| lisinopril 10mg tablets (teva uk ltd) | 19223 |
| xuret 0.5mg tablet (galen ltd) | 19352 |
| disogram sr 240mg capsules (ranbaxy (uk) ltd) | 19426 |
| eucardic 25mg tablets (roche products ltd) | 19437 |
| disogram sr 300mg capsules (ranbaxy (uk) ltd) | 19440 |
| ranvera mr 240mg tablets (ranbaxy (uk) ltd) | 19457 |
| verapamil 240mg modified-release tablets (a a h pharmaceuticals ltd) | 19459 |
| verapamil 180mg modified-release / trandolapril 2mg capsules | 19690 |
| alphavase 5 tablets (ashbourne pharmaceuticals ltd) | 19823 |
| cardicor 3.75mg tablets (merck serono ltd) | 19853 |
| cardicor 10mg tablets (merck serono ltd) | 19858 |
| hydrochlorothiazide with amiloride 25mgwith2.5mg tablet | 19890 |
| serpasil -esidrex tablet (novartis pharmaceuticals uk ltd) | 19892 |
| visken 15mg tablet (sovereign medical ltd) | 20012 |
| methyclothiazide 5mg tablet | 20057 |
| amil-co 5mg/50mg tablets (ivax pharmaceuticals uk ltd) | 20066 |
| lopresor sr 200mg tablets (recordati pharmaceuticals ltd) | 20082 |
| metoprolol 200mg modified-release / hydrochlorothiazide 25mg tablets | 20093 |
| olmetec 40mg tablets (daiichi sankyo uk ltd) | 20117 |
| cardilate mr 20mg tablets (ivax pharmaceuticals uk ltd) | 20257 |
| nifedipress mr 20mg modified-release tablet (generics (uk) ltd) | 20311 |
| centyl k 2.5mg+7.7mmol tablet (edwin burgess ltd) | 20426 |
| centyl k modified-release tablets (leo pharma) | 20431 |
| felendil xl 10mg modified-release tablet (ratiopharm uk ltd) | 20459 |

| | |
|---|---|
| half beta-prograne 80mg modified-release capsules (tillomed laboratories ltd) | 20468 |
| atenix 100 tablets (ashbourne pharmaceuticals ltd) | 20502 |
| frumax 40mg tablet (ashbourne pharmaceuticals ltd) | 20538 |
| tarka modified-release capsules (abbott laboratories ltd) | 20579 |
| nifedipress mr 20 tablets (teva uk ltd) | 20591 |
| bi-carzem sr 60mg modified-release capsule (tillomed laboratories ltd) | 20642 |
| serpasil 250microgram tablet (novartis pharmaceuticals uk ltd) | 20656 |
| reserpine 250micrograms tablet | 20690 |
| atenamin 25mg tablet (opd pharm) | 20728 |
| tensopril 12.5mg tablets (teva uk ltd) | 20849 |
| angiopine 10 capsules (ashbourne pharmaceuticals ltd) | 20878 |
| zemtard 240 xl capsules (galen ltd) | 20890 |
| prestim forte tablet (leo pharma) | 21025 |
| vascace 500microgram tablets (roche products ltd) | 21053 |
| atenamin 50mg tablet (opd pharm) | 21133 |
| dilcardia sr 60mg capsules (generics (uk) ltd) | 21145 |
| felodipine 2.5mg modified-release / ramipril 2.5mg tablets | 21162 |
| hydrochlorothiazide with timolol and amiloride 25mg with 10mg with 2.5mg tablet | 21182 |
| hypolar retard 10mg tablets (sandoz ltd) | 21216 |
| caralpha 20mg/12.5mg tablets (actavis uk ltd) | 21231 |
| nifedipress mr 10mg modified-release tablet (actavis uk ltd) | 21245 |
| hydromet tablet (msd thomas morson pharmaceuticals) | 21346 |
| cozaar-comp 100mg/25mg tablets (merck sharp & dohme ltd) | 21423 |
| serpasil 100microgram tablet (novartis pharmaceuticals uk ltd) | 21502 |
| diltiazem 60mg modified-release tablets (a a h pharmaceuticals ltd) | 21763 |
| diltiazem hcl 60mg tablet (generics (uk) ltd) | 21773 |
| diltiazem 60mg modified-release tablets (teva uk ltd) | 21778 |
| retalzem 60 modified-release tablets (kent pharmaceuticals ltd) | 21795 |
| berkozide 2.5mg tablet (berk pharmaceuticals ltd) | 21803 |
| propanix 80mg tablet (ashbourne pharmaceuticals ltd) | 21838 |
| berkolol 80mg tablet (berk pharmaceuticals ltd) | 21839 |
| dryptal 40mg tablet (berk pharmaceuticals ltd) | 21849 |
| berkolol 40mg tablet (berk pharmaceuticals ltd) | 21866 |
| berkozide 5mg tablet (berk pharmaceuticals ltd) | 21867 |
| angiopine 5mg capsule (ashbourne pharmaceuticals ltd) | 21872 |
| atenix co 50 tablets (ashbourne pharmaceuticals ltd) | 21873 |
| oxyprenix sr 160mg tablets | 21885 |
| nifedipress mr 20 tablets (actavis uk ltd) | 21886 |
| bipranix 10mg tablets (ashbourne pharmaceuticals ltd) | 21905 |
| spirospare 25mg tablet (ashbourne pharmaceuticals ltd) | 21911 |
| optil 60mg modified-release tablets (opus pharmaceuticals ltd) | 21918 |
| froop co 5mg/40mg tablets (ashbourne pharmaceuticals ltd) | 21938 |
| kaplon 12.5mg tablets (teva uk ltd) | 21943 |
| bipranix 5mg tablets (ashbourne pharmaceuticals ltd) | 21966 |
| calanif 10mg capsule (berk pharmaceuticals ltd) | 22019 |
| calcilat 10mg capsule (eastern pharmaceuticals ltd) | 22142 |
| half propanix la 80mg modified-release capsule (ashbourne pharmaceuticals ltd) | 22208 |
| nimodrel 10mg modified-release tablet (opus pharmaceuticals ltd) | 22217 |
| mibefradil 100mg tablet | 22241 |
| ednyt 20mg tablet (dominion pharma) | 22439 |
| britiazim 60mg modified-release tablet (thames laboratories ltd) | 22619 |
| torem 10mg tablets (meda pharmaceuticals ltd) | 22658 |
| slofedipine 20mg tablets (sterwin medicines) | 22696 |
| labrocol 200mg tablet (lagap) | 22793 |
| securon 160mg tablet (abbott laboratories ltd) | 22826 |
| reserpine 100micrograms tablet | 22853 |
| bendroflumethiazide 2.5mg with propanolol 80mg capsules | 22912 |
| hydrochlorothiazide with amiloride 50mg with 5mg tablet | 22923 |

| | |
|---|---|
| spirospare 100 tablets (ashbourne pharmaceuticals ltd) | 23091 |
| bendroflumethiazide 5mg with propanolol 160mg modified-release capsules | 23131 |
| nadolol 40mg / bendroflumethiazide 5mg tablets | 23134 |
| bi-carzem sr 90mg modified-release capsule (tillomed laboratories ltd) | 23233 |
| pralenal 10 tablets (opus pharmaceuticals ltd) | 23252 |
| betadur cr 160mg modified-release capsule (monmouth pharmaceuticals ltd) | 23326 |
| hypercal 2mg tablet (carlton laboratories) | 23345 |
| catapres 300microgram tablets (boehringer ingelheim ltd) | 23380 |
| bendroflumethiazide 5mg tablets (a a h pharmaceuticals ltd) | 23427 |
| hydrochlorothiazide with valsartan 25mg with 160mg tablet | 23456 |
| hypovase benign prostatic hyperplasia 2mg tablet (pfizer ltd) | 23459 |
| tensopril 50mg tablets (teva uk ltd) | 23478 |
| adizem xl plus 150mg+12.5mg modified-release capsule (napp pharmaceuticals ltd) | 23505 |
| sloprolol 160mg capsule (c p pharmaceuticals ltd) | 23587 |
| vascace 0.25mg tablet (roche products ltd) | 23642 |
| optil sr 90mg modified-release capsule (opus pharmaceuticals ltd) | 23733 |
| hypolar xl 30 tablets (sandoz ltd) | 23736 |
| nisoldipine 10mg modified-release tablets | 23805 |
| nisoldipine 30mg modified-release tablets | 23823 |
| berkatens 40mg tablet (berk pharmaceuticals ltd) | 23872 |
| vasetic tablet (shire pharmaceuticals ltd) | 24008 |
| bisoprolol 5mg tablets (teva uk ltd) | 24083 |
| trasicor 40mg tablets (amdipharm plc) | 24094 |
| neo-bendromax 2.5mg tablet (ashbourne pharmaceuticals ltd) | 24189 |
| neo-bendromax 5mg tablet (ashbourne pharmaceuticals ltd) | 24190 |
| antipressan 50mg tablets (teva uk ltd) | 24191 |
| antipressan 100mg tablets (teva uk ltd) | 24195 |
| dopamet 250mg tablet (berk pharmaceuticals ltd) | 24196 |
| berkolol 160mg tablet (berk pharmaceuticals ltd) | 24218 |
| nimodrel 20mg modified-release tablet (opus pharmaceuticals ltd) | 24228 |
| hydrochlorothiazide with valsartan 12.5mg with 80mg tablet | 24268 |
| totaretic 100mg+25mg tablet (c p pharmaceuticals ltd) | 24280 |
| diovan 40mg tablets (novartis pharmaceuticals uk ltd) | 24359 |
| cardioplen xl 5mg tablets (chiesi ltd) | 24365 |
| cardioplen xl 10mg tablets (chiesi ltd) | 24366 |
| captomex 50mg tablets (actavis uk ltd) | 24482 |
| hydrochlorothiazide with valsartan 12.5mg with 160mg tablet | 24484 |
| hydrochlorothiazide with losartan 25mg with 100mg tablet | 24632 |
| lasipressin tablet (hoechst uk ltd) | 24832 |
| amilospare tablet (ashbourne pharmaceuticals ltd) | 24893 |
| hypovase benign prostatic hyperplasia 500microgram tablet (pfizer ltd) | 25047 |
| berkatens 80mg tablet (berk pharmaceuticals ltd) | 25059 |
| nifopress mr 20mg tablets (teva uk ltd) | 25132 |
| metalpha 500mg tablet (ashbourne pharmaceuticals ltd) | 25275 |
| dopamet 500mg tablet (berk pharmaceuticals ltd) | 25289 |
| furosemide 500mg tablets (a a h pharmaceuticals ltd) | 25334 |
| rapranol sr 160mg capsules (ranbaxy (uk) ltd) | 25359 |
| prestim tablets (meda pharmaceuticals ltd) | 25363 |
| rapranol sr 80mg capsules (ranbaxy (uk) ltd) | 25367 |
| co-diovan 160mg/25mg tablets (novartis pharmaceuticals uk ltd) | 25382 |
| decaserpyl 10mg tablet (roussel laboratories ltd) | 25393 |
| clopamide 5mg with pindolol 10mg tablets | 25462 |
| cascor 2mg tablets (ranbaxy (uk) ltd) | 25487 |
| diatensec 50mg tablet (pharmacia ltd) | 25494 |
| hypertane 50 tablet (schwarz pharma ltd) | 25500 |
| spiro-co 50mg+50mg tablet (ivax pharmaceuticals uk ltd) | 25505 |
| cascor 4mg tablets (ranbaxy (uk) ltd) | 25551 |
| felogen xl 5mg tablets (generics (uk) ltd) | 25572 |

| | |
|---|---|
| apsolox 80mg tablet (approved prescription services ltd) | 25644 |
| seominal tablet (sterling-winthrop) | 25645 |
| nivaten retard 20mg modified-release tablet (actavis uk ltd) | 25646 |
| furosemide 40mg tablets (generics (uk) ltd) | 25717 |
| timolol maleate with amiloride and hydrochlorothiazide tablet | 25730 |
| dilcardia sr 120mg capsules (generics (uk) ltd) | 25777 |
| nifedipine 20mg modified-release tablets (a a h pharmaceuticals ltd) | 25919 |
| co-amilofruse 2.5mg/20mg tablets (wockhardt uk ltd) | 25965 |
| captomex 12.5mg tablets (actavis uk ltd) | 25998 |
| antipressan 25mg tablets (teva uk ltd) | 26211 |
| berkamil 5mg tablet (berk pharmaceuticals ltd) | 26217 |
| zida-co 5mg+50mg tablet (opus pharmaceuticals ltd) | 26219 |
| delvas tablet (berk pharmaceuticals ltd) | 26220 |
| propanix la 160mg modified-release capsule (ashbourne pharmaceuticals ltd) | 26228 |
| beta-prograne 160mg modified-release capsules (tillomed laboratories ltd) | 26229 |
| alphavase 500microgram tablet (ashbourne pharmaceuticals ltd) | 26237 |
| alphavase 1 tablets (ashbourne pharmaceuticals ltd) | 26238 |
| tenchlor 100mg/25mg tablets (teva uk ltd) | 26248 |
| berkatens 160mg tablet (berk pharmaceuticals ltd) | 26252 |
| lopranol la 160mg capsule (opus pharmaceuticals ltd) | 26255 |
| opumide 2.5mg tablet (opus pharmaceuticals ltd) | 26256 |
| calanif 5mg capsule (berk pharmaceuticals ltd) | 26265 |
| optil sr 120mg modified-release capsule (opus pharmaceuticals ltd) | 26267 |
| optil sr 180mg modified-release capsule (opus pharmaceuticals ltd) | 26269 |
| optil xl 300mg modified-release capsule (opus pharmaceuticals ltd) | 26270 |
| nindaxa 2.5 tablets (ashbourne pharmaceuticals ltd) | 26275 |
| diuresal 40mg tablet (lagap) | 26292 |
| optil xl 240mg modified-release capsule (opus pharmaceuticals ltd) | 26309 |
| cabren 10mg modified-release tablets (teva uk ltd) | 26337 |
| dilcardia xl 240mg modified-release capsule (generics (uk) ltd) | 26460 |
| zemret xl 240mg capsule (neo laboratories ltd) | 26463 |
| furosemide with penbutolol tablet | 26529 |
| totaretic 50mg+12.5mg tablet (c p pharmaceuticals ltd) | 26741 |
| zildil sr 60mg capsules (chanelle medical uk ltd) | 26759 |
| kaplon 25mg tablets (teva uk ltd) | 26995 |
| diltiazem sr 90mg capsule (hillcross pharmaceuticals ltd) | 27135 |
| diltiazem 90mg modified-release tablets (a a h pharmaceuticals ltd) | 27136 |
| sildenafil 20mg tablets | 27137 |
| bendroflumethiazide 2.5mg tablets (wockhardt uk ltd) | 27256 |
| oxprenolol 40mg tablet (actavis uk ltd) | 27357 |
| kenzem sr 90mg capsules (kent pharmaceuticals ltd) | 27401 |
| furosemide 40mg tablets (wockhardt uk ltd) | 27447 |
| olmetec plus 20mg/25mg tablets (daiichi sankyo uk ltd) | 27520 |
| diltiazem hcl 300mg capsule (pliva pharma ltd) | 27685 |
| bendroflumethiazide 2.5mg tablets (ivax pharmaceuticals uk ltd) | 27689 |
| furosemide 40mg tablets (a a h pharmaceuticals ltd) | 27690 |
| furosemide 40mg tablets (kent pharmaceuticals ltd) | 27696 |
| propranolol 40mg tablets (actavis uk ltd) | 27700 |
| metoros ls 95mg tablet (geigy pharmaceuticals) | 27719 |
| furosemide 20mg tablets (generics (uk) ltd) | 27926 |
| nadolol 80mg / bendroflumethiazide 5mg tablets | 27946 |
| natramid 2.5mg tablet (trinity pharmaceuticals ltd) | 27957 |
| apsolol 40mg tablet (approved prescription services ltd) | 27964 |
| angilol 10mg tablet (ddsa pharmaceuticals ltd) | 28048 |
| enalapril 2.5mg tablets (teva uk ltd) | 28127 |
| propranolol 80mg modified-release capsule (actavis uk ltd) | 28128 |
| co-amilofruse 5mg/40mg tablets (teva uk ltd) | 28129 |
| kalspare ls tablet (dominion pharma) | 28157 |

| | |
|---|---|
| hydrochlorothiazide with atenolol and amiloride capsule | 28177 |
| triapin 2.5mg/2.5mg modified-release tablets (sanofi) | 28438 |
| lopace 5mg capsules (discovery pharmaceuticals ltd) | 28586 |
| bethanidine sulphate 50mg tablets | 28676 |
| nifedipine 10mg modified-release tablets (a a h pharmaceuticals ltd) | 28688 |
| cartrol 10mg tablet (novartis consumer health uk ltd) | 28700 |
| neofel xl 5mg tablets (kent pharmaceuticals ltd) | 28721 |
| perdix 7.5mg tablets (ucb pharma ltd) | 28724 |
| perdix 15mg tablets (ucb pharma ltd) | 28725 |
| methyldopa with hydrochlorothiazide tablet | 28738 |
| half propatard la 80mg modified-release capsule (galen ltd) | 28788 |
| captomex 25mg tablets (actavis uk ltd) | 28820 |
| verapamil hc 80mg tablet (celltech pharma europe ltd) | 28843 |
| berkatens 120mg tablet (berk pharmaceuticals ltd) | 28844 |
| odrik 2mg capsules (aventis pharma) | 28902 |
| bi-carzem sr 120mg modified-release capsule (tillomed laboratories ltd) | 28949 |
| bedranol sr 160mg capsules (sandoz ltd) | 28996 |
| neofel xl 10mg tablets (kent pharmaceuticals ltd) | 29044 |
| gopten 4mg capsules (abbott laboratories ltd) | 29130 |
| felendil xl 2.5mg modified-release tablet (ratiopharm uk ltd) | 29145 |
| trasicor 80mg tablets (amdipharm plc) | 29180 |
| methoserpidine 10mg tablet | 29187 |
| slow-pren 160mg tablet (ivax pharmaceuticals uk ltd) | 29230 |
| atenolol 25mg tablets (teva uk ltd) | 29368 |
| spiretic 100mg tablet (ddsa pharmaceuticals ltd) | 29397 |
| atenamin 100mg tablet (opd pharm) | 29398 |
| hydrochlorothiazide with metoprolol tartrate 12.5mg with 100mg tablet | 29427 |
| bendogen 10mg tablet (lagap) | 29443 |
| hydroflumethiazide with spironolactone 25mg+25mg tablet | 29529 |
| bosentan 62.5mg tablets | 29560 |
| bosentan 125mg tablets | 29561 |
| dopamet 125mg tablet (berk pharmaceuticals ltd) | 29570 |
| betim 10mg tablets (meda pharmaceuticals ltd) | 29610 |
| lopace 2.5mg capsules (discovery pharmaceuticals ltd) | 29627 |
| olmetec plus 20mg/12.5mg tablets (daiichi sankyo uk ltd) | 29634 |
| verapress mr 240mg tablets (teva uk ltd) | 29637 |
| calazem 60mg modified-release tablet (berk pharmaceuticals ltd) | 29676 |
| inspra 50mg tablets (pfizer ltd) | 29694 |
| methoserpidine with benzthiazide tablet | 29696 |
| mepranix 50mg tablet (ashbourne pharmaceuticals ltd) | 29762 |
| propanix 160mg tablet (ashbourne pharmaceuticals ltd) | 29763 |
| furosemide 20mg tablet (c p pharmaceuticals ltd) | 29780 |
| carteolol hcl 10mg tablets | 29827 |
| centyl 5mg tablet (edwin burgess ltd) | 29991 |
| metoros 190mg tablet (novartis pharmaceuticals uk ltd) | 29998 |
| tensopril 25mg tablets (teva uk ltd) | 30039 |
| abicol tablet (knoll ltd) | 30129 |
| diltiazem 120mg modified-release capsules | 30197 |
| nifedipine 30mg modified-release tablets | 30199 |
| diltiazem 180mg modified-release capsules | 30242 |
| benthiazide with triamterene capsules | 30272 |
| mepranix 100mg tablet (ashbourne pharmaceuticals ltd) | 30400 |
| ethimil mr 240mg tablets (genus pharmaceuticals ltd) | 30462 |
| coroday mr 20mg tablets (generics (uk) ltd) | 30473 |
| amiloride with timolol with hydrochlorothiazide tablets | 30519 |
| felogen xl 10mg tablets (generics (uk) ltd) | 30557 |
| furosemide 20mg tablets (a a h pharmaceuticals ltd) | 30625 |
| vasaten 50mg tablet (shire pharmaceuticals ltd) | 30636 |

| | |
|---|---|
| hypercal b tablet (carlton laboratories) | 30691 |
| lidoflazine 120mg tablet | 30758 |
| labetalol 200mg tablets (a a h pharmaceuticals ltd) | 30770 |
| co-amilofruse 5mg+40mg tablet (berk pharmaceuticals ltd) | 30773 |
| betinex 1mg tablet (berk pharmaceuticals ltd) | 30913 |
| cabren 2.5mg modified-release tablets (teva uk ltd) | 30915 |
| lisinopril 2.5mg tablets (teva uk ltd) | 30921 |
| revatio 20mg tablets (pfizer ltd) | 30967 |
| cabren 5mg modified-release tablets (teva uk ltd) | 30991 |
| amias 32mg tablets (takeda uk ltd) | 31072 |
| spiro-co 25mg+25mg tablet (ivax pharmaceuticals uk ltd) | 31131 |
| co-amilozide 5mg/50mg tablets (ivax pharmaceuticals uk ltd) | 31150 |
| propranolol 80mg tablets (generics (uk) ltd) | 31214 |
| spironolactone 100mg tablets (a a h pharmaceuticals ltd) | 31219 |
| hydralazine 25mg tablets (a a h pharmaceuticals ltd) | 31220 |
| odrik 500microgram capsules (aventis pharma) | 31307 |
| syscor mr 30 tablets (forest laboratories uk ltd) | 31336 |
| syscor mr 20 tablets (forest laboratories uk ltd) | 31337 |
| tenchlor 50mg/12.5mg tablets (teva uk ltd) | 31470 |
| bi-carzem xl 240mg capsule (tillomed laboratories ltd) | 31489 |
| spironolactone 25mg tablets (teva uk ltd) | 31529 |
| atenolol 25mg tablets (kent pharmaceuticals ltd) | 31536 |
| furosemide 20mg tablets (actavis uk ltd) | 31548 |
| bendroflumethiazide 2.5mg tablets (teva uk ltd) | 31670 |
| diltiazem hcl 120mg modified-release tablet (actavis uk ltd) | 31676 |
| co-tenidone 50mg/12.5mg tablets (actavis uk ltd) | 31708 |
| verapamil 80mg tablets (a a h pharmaceuticals ltd) | 31711 |
| enalapril 20mg tablets (actavis uk ltd) | 31716 |
| zildil sr 120mg capsules (chanelle medical uk ltd) | 31737 |
| amlostin 10mg tablets (discovery pharmaceuticals ltd) | 31761 |
| co-amilofruse 5mg/40mg tablets (wockhardt uk ltd) | 31773 |
| propranolol 40mg tablets (generics (uk) ltd) | 31776 |
| odrik 1mg capsules (aventis pharma) | 31810 |
| bendroflumethiazide 5mg tablets (wockhardt uk ltd) | 31820 |
| angilol 80mg tablet (ddsa pharmaceuticals ltd) | 31833 |
| bumetanide 1mg tablets (c p pharmaceuticals ltd) | 31932 |
| atenolol 100mg tablets (ivax pharmaceuticals uk ltd) | 31934 |
| kaplon 50mg tablets (teva uk ltd) | 32048 |
| diltiazem hcl 120mg modified-release capsule (hillcross pharmaceuticals ltd) | 32089 |
| bumetanide 1mg tablets (a a h pharmaceuticals ltd) | 32091 |
| co-tenidone 50mg/12.5mg tablets (a a h pharmaceuticals ltd) | 32094 |
| bisoprolol 5mg tablets (generics (uk) ltd) | 32114 |
| propranolol 80mg modified-release capsule (lagap) | 32162 |
| capto-co 25mg+50mg tablet (ivax pharmaceuticals uk ltd) | 32166 |
| enalapril 10mg tablets (a a h pharmaceuticals ltd) | 32241 |
| diltiazem hcl 60mg tablet (c p pharmaceuticals ltd) | 32262 |
| ecopace 25mg tablets (amco) | 32514 |
| congescor 2.5mg tablets (tillomed laboratories ltd) | 32552 |
| tanatril 20mg tablets (chiesi ltd) | 32560 |
| verapamil 40mg tablets (generics (uk) ltd) | 32590 |
| amlodipine 5mg tablets (a a h pharmaceuticals ltd) | 32595 |
| lisinopril 10mg tablets (sandoz ltd) | 32597 |
| vivacor 10mg tablets (lexon (uk) ltd) | 32630 |
| dilcardia xl 120mg modified-release capsule (generics (uk) ltd) | 32658 |
| visken 15mg tablets (amco) | 32787 |
| metoprolol 50mg tablets (generics (uk) ltd) | 32836 |
| spironolactone 50mg tablets (teva uk ltd) | 32837 |
| ramipril 1.25mg capsules (teva uk ltd) | 32857 |

| | |
|---|---|
| diltiazem 60mg modified-release tablets (sterwin medicines) | 32870 |
| furosemide 40mg tablets (ranbaxy (uk) ltd) | 32896 |
| methyldopa 250mg tablets (actavis uk ltd) | 32913 |
| amlodipine 5mg tablets (ivax pharmaceuticals uk ltd) | 32917 |
| furosemide 20mg tablets (sandoz ltd) | 32918 |
| felodipine 10mg modified-release tablet (sandoz ltd) | 32922 |
| lopace 10mg capsules (discovery pharmaceuticals ltd) | 32934 |
| nimodrel xl 30mg tablets (zurich pharmaceuticals) | 33025 |
| ednyt 5mg tablet (dominion pharma) | 33057 |
| enalapril 20mg tablets (a a h pharmaceuticals ltd) | 33078 |
| atenolol 100mg tablets (generics (uk) ltd) | 33079 |
| indapamide 2.5mg tablets (teva uk ltd) | 33083 |
| atenolol 100mg tablets (a a h pharmaceuticals ltd) | 33085 |
| felodipine 10mg modified-release tablets (a a h pharmaceuticals ltd) | 33091 |
| atenolol 50mg tablets (a a h pharmaceuticals ltd) | 33092 |
| clonidine 25microgram tablets (sandoz ltd) | 33093 |
| doxazosin 2mg tablets (generics (uk) ltd) | 33094 |
| perindopril erbumine 4mg tablets (a a h pharmaceuticals ltd) | 33095 |
| atenolol 100mg tablets (wockhardt uk ltd) | 33184 |
| moxonidine 200microgram tablets (sandoz ltd) | 33322 |
| lisinopril 20mg / hydrochlorothiazide 12.5mg tablets (teva uk ltd) | 33353 |
| carvedilol 12.5mg tablets (genus pharmaceuticals ltd) | 33374 |
| probeta la 160mg capsule (trinity pharmaceuticals ltd) | 33376 |
| bendroflumethiazide 2.5mg tablets (generics (uk) ltd) | 33415 |
| verapamil 40mg tablets (actavis uk ltd) | 33471 |
| co-amilofruse 5mg/40mg tablets (generics (uk) ltd) | 33527 |
| oxprenolol sr 160mg modified-release tablet (hillcross pharmaceuticals ltd) | 33569 |
| slo-pro 160mg capsules (generics (uk) ltd) | 33602 |
| propranolol 80mg tablets (a a h pharmaceuticals ltd) | 33644 |
| captopril 12.5mg tablet (generics (uk) ltd) | 33646 |
| atenolol 50mg tablets (generics (uk) ltd) | 33650 |
| bendroflumethiazide 2.5mg tablets (a a h pharmaceuticals ltd) | 33651 |
| atenolol 25mg tablets (a a h pharmaceuticals ltd) | 33657 |
| co-amilofruse 5mg/40mg tablets (a a h pharmaceuticals ltd) | 33658 |
| hydrochlorothiazide with metoprolol tartrate 25mg with 200mg modified-release tablet | 33659 |
| ramipril 2.5mg capsules (ranbaxy (uk) ltd) | 33811 |
| apsolol 160mg tablet (approved prescription services ltd) | 33836 |
| amiloride 5mg tablets (a a h pharmaceuticals ltd) | 33837 |
| bisoprolol 10mg tablets (actavis uk ltd) | 33839 |
| atenolol 50mg tablets (actavis uk ltd) | 33850 |
| ramipril 10mg capsules (teva uk ltd) | 33894 |
| congescor 1.25mg tablets (tillomed laboratories ltd) | 33909 |
| parmid xl 5mg tablets (sandoz ltd) | 33932 |
| lisinopril 10mg tablets (generics (uk) ltd) | 33977 |
| furosemide 40mg tablets (actavis uk ltd) | 34006 |
| co-tenidone 100mg/25mg tablets (ivax pharmaceuticals uk ltd) | 34012 |
| co-tenidone 50mg/12.5mg tablets (ivax pharmaceuticals uk ltd) | 34034 |
| bendroflumethiazide 2.5mg tablets (actavis uk ltd) | 34059 |
| metoprolol 100mg tablets (teva uk ltd) | 34092 |
| amlodipine 10mg tablets (a a h pharmaceuticals ltd) | 34093 |
| metoprolol 50mg tablets (a a h pharmaceuticals ltd) | 34094 |
| nifedipine mr 20mg modified-release tablet (ivax pharmaceuticals uk ltd) | 34101 |
| nifedipine 60mg modified-release tablet | 34115 |
| bendroflumethiazide 5mg tablets (actavis uk ltd) | 34124 |
| metoprolol 100mg tablets (a a h pharmaceuticals ltd) | 34125 |
| nifedipine mr 10mg modified-release tablet (ivax pharmaceuticals uk ltd) | 34146 |
| labetalol 100mg tablet (c p pharmaceuticals ltd) | 34171 |
| labetalol 100mg tablets (a a h pharmaceuticals ltd) | 34177 |

| | |
|---|---|
| propranolol la 80mg modified-release capsule (approved prescription services ltd) | 34185 |
| nifedipine 10mg modified-release tablet (generics (uk) ltd) | 34187 |
| labetalol 200mg tablet (celltech pharma europe ltd) | 34188 |
| propranolol sr 160mg modified-release capsule (c p pharmaceuticals ltd) | 34208 |
| propranolol 160mg tablets (actavis uk ltd) | 34214 |
| nifedipine 10mg capsule (berk pharmaceuticals ltd) | 34247 |
| atenolol 50mg tablets (sandoz ltd) | 34265 |
| co-amilofruse 2.5mg/20mg tablets (sandoz ltd) | 34280 |
| spironolactone 25mg tablets (a a h pharmaceuticals ltd) | 34296 |
| amiloride 5mg tablets (teva uk ltd) | 34324 |
| doxazosin 1mg tablets (teva uk ltd) | 34342 |
| spironolactone 25mg tablets (actavis uk ltd) | 34347 |
| ramipril 10mg capsules (genus pharmaceuticals ltd) | 34357 |
| atenolol 50mg tablets (teva uk ltd) | 34365 |
| co-amilozide 2.5mg/25mg tablets (wockhardt uk ltd) | 34367 |
| sotalol 40mg tablets (a a h pharmaceuticals ltd) | 34371 |
| furosemide 40mg tablets (teva uk ltd) | 34374 |
| diltiazem hcl 90mg modified-release capsule (hillcross pharmaceuticals ltd) | 34377 |
| propranolol 10mg tablets (a a h pharmaceuticals ltd) | 34378 |
| ramipril 5mg capsules (zentiva) | 34382 |
| ramipril 5mg capsules (genus pharmaceuticals ltd) | 34390 |
| enalapril 5mg tablet (dowelhurst ltd) | 34400 |
| metoprolol 50mg tablets (teva uk ltd) | 34407 |
| ramipril 5mg capsules (teva uk ltd) | 34412 |
| ramipril 5mg capsules (generics (uk) ltd) | 34429 |
| metoprolol 50mg tablets (actavis uk ltd) | 34430 |
| ramipril 2.5mg capsules (zentiva) | 34431 |
| ramipril 2.5mg capsules (genus pharmaceuticals ltd) | 34432 |
| atenolol 50mg tablets (wockhardt uk ltd) | 34443 |
| co-tenidone 50mg/12.5mg tablets (generics (uk) ltd) | 34449 |
| enalapril 20mg tablets (generics (uk) ltd) | 34453 |
| lisinopril 5mg tablets (generics (uk) ltd) | 34471 |
| diltiazem hcl 90mg modified-release tablet (ivax pharmaceuticals uk ltd) | 34475 |
| ramipril 2.5mg capsules (teva uk ltd) | 34490 |
| atenolol 25mg tablets (generics (uk) ltd) | 34492 |
| carvedilol 12.5mg tablets (actavis uk ltd) | 34501 |
| ramipril 2.5mg capsules (sandoz ltd) | 34505 |
| metoprolol 100mg tablets (generics (uk) ltd) | 34509 |
| sotalol 80mg tablets (generics (uk) ltd) | 34520 |
| nifedipine 5mg capsules (a a h pharmaceuticals ltd) | 34522 |
| ramipril 2.5mg capsules (a a h pharmaceuticals ltd) | 34528 |
| ramipril 5mg capsules (sandoz ltd) | 34539 |
| ramipril 5mg capsules (a a h pharmaceuticals ltd) | 34540 |
| captopril 12.5mg tablet (ivax pharmaceuticals uk ltd) | 34544 |
| indapamide 2.5mg tablets (generics (uk) ltd) | 34551 |
| doxazosin 4mg tablets (generics (uk) ltd) | 34553 |
| furosemide 40mg tablets (ivax pharmaceuticals uk ltd) | 34557 |
| captopril 25mg tablet (ivax pharmaceuticals uk ltd) | 34562 |
| ramipril 2.5mg capsules (generics (uk) ltd) | 34567 |
| atenolol 25mg tablets (wockhardt uk ltd) | 34575 |
| diltiazem hcl 60mg modified-release tablet (kent pharmaceuticals ltd) | 34581 |
| ramipril 10mg capsule (dexcel-pharma ltd) | 34583 |
| metoprolol 50mg tablets (ivax pharmaceuticals uk ltd) | 34584 |
| atenolol 25mg tablets (sandoz ltd) | 34585 |
| ramipril 5mg capsule (dexcel-pharma ltd) | 34589 |
| sotalol 40mg tablets (teva uk ltd) | 34600 |
| doxazosin 1mg tablets (generics (uk) ltd) | 34601 |
| bendroflumethiazide 2.5mg tablets (sovereign medical ltd) | 34602 |

| | |
|---|---|
| nifedipine 5mg capsules (ivax pharmaceuticals uk ltd) | 34607 |
| bumetanide 5mg tablets (teva uk ltd) | 34613 |
| co-amilofruse 10mg/80mg tablets (wockhardt uk ltd) | 34622 |
| doxazosin 2mg tablets (a a h pharmaceuticals ltd) | 34625 |
| sotalol 40mg tablet (tillomed laboratories ltd) | 34640 |
| ramipril 10mg capsules (generics (uk) ltd) | 34651 |
| ramipril 5mg capsule (sovereign medical ltd) | 34652 |
| ramipril 10mg capsules (zentiva) | 34657 |
| sotalol 80mg tablets (sandoz ltd) | 34690 |
| atenolol 50mg tablets (kent pharmaceuticals ltd) | 34695 |
| lisinopril 20mg tablets (generics (uk) ltd) | 34696 |
| ramipril 1.25mg capsules (zentiva) | 34698 |
| ramipril 10mg capsules (sandoz ltd) | 34710 |
| enalapril 20mg tablets (kent pharmaceuticals ltd) | 34712 |
| doxazosin 1mg tablets (a a h pharmaceuticals ltd) | 34715 |
| captopril 50mg tablet (generics (uk) ltd) | 34719 |
| ramipril 2.5mg capsule (dexcel-pharma ltd) | 34732 |
| carvedilol 6.25mg tablets (actavis uk ltd) | 34740 |
| carvedilol 3.125mg tablets (ivax pharmaceuticals uk ltd) | 34741 |
| amiloride 5mg tablets (actavis uk ltd) | 34750 |
| atenolol 100mg tablets (sandoz ltd) | 34754 |
| enalapril 20mg tablets (ivax pharmaceuticals uk ltd) | 34768 |
| propranolol 10mg tablets (actavis uk ltd) | 34783 |
| enalapril 20mg tablets (sandoz ltd) | 34798 |
| lisinopril 20mg tablets (zentiva) | 34799 |
| bendroflumethiazide 2.5mg tablet (regent laboratories ltd) | 34803 |
| propranolol 10mg tablets (teva uk ltd) | 34804 |
| bisoprolol 10mg tablets (generics (uk) ltd) | 34821 |
| diltiazem hcl 120mg modified-release tablet (ivax pharmaceuticals uk ltd) | 34824 |
| co-tenidone 50mg/12.5mg tablets (teva uk ltd) | 34825 |
| metoprolol 100mg tablets (actavis uk ltd) | 34854 |
| propranolol 80mg capsule (ivax pharmaceuticals uk ltd) | 34867 |
| propranolol 40mg tablets (teva uk ltd) | 34868 |
| ramipril 10mg capsule (sovereign medical ltd) | 34877 |
| atenolol 50mg tablet (berk pharmaceuticals ltd) | 34882 |
| propranolol 160mg modified-release capsule (sandoz ltd) | 34884 |
| metoprolol 50mg tablet (berk pharmaceuticals ltd) | 34890 |
| ramipril 10mg capsule (ivax pharmaceuticals uk ltd) | 34893 |
| co-tenidone 100mg/25mg tablets (a a h pharmaceuticals ltd) | 34899 |
| spironolactone 25mg tablets (ivax pharmaceuticals uk ltd) | 34908 |
| metoprolol 50mg tablets (sandoz ltd) | 34925 |
| bumetanide 1mg tablets (generics (uk) ltd) | 34934 |
| captopril 25mg tablet (lagap) | 34936 |
| captopril 50mg tablet (ivax pharmaceuticals uk ltd) | 34937 |
| ramipril 10mg capsules (a a h pharmaceuticals ltd) | 34943 |
| propranolol 160mg modified-release capsule (lagap) | 34945 |
| propranolol 160mg modified-release capsule (actavis uk ltd) | 34949 |
| enalapril 10mg tablets (generics (uk) ltd) | 34952 |
| enalapril 20mg tablets (zentiva) | 34953 |
| verapamil 40mg tablets (a a h pharmaceuticals ltd) | 34959 |
| bisoprolol 5mg tablets (actavis uk ltd) | 34963 |
| nifedipine 5mg capsules (teva uk ltd) | 34975 |
| atenolol 25mg tablets (tillomed laboratories ltd) | 34976 |
| celectol 200mg tablets (zentiva) | 35054 |
| trasicor 20mg tablets (amdipharm plc) | 35062 |
| vascalpha 5mg modified-release tablets (actavis uk ltd) | 35084 |
| exforge 10mg/160mg tablets (novartis pharmaceuticals uk ltd) | 35096 |
| valsartan 160mg with amlodipine 5mg tablets | 35173 |

| | |
|---|---|
| valsartan 80mg with amlodipine 5mg tablets | 35174 |
| amlodipine 10mg / valsartan 160mg tablets | 35189 |
| coaprovel 300mg/25mg tablets (sanofi) | 35196 |
| doxadura xl 4mg tablets (discovery pharmaceuticals ltd) | 35272 |
| valsartan 160mg with amlodipine 10mg tablets | 35304 |
| exforge 5mg/80mg tablets (novartis pharmaceuticals uk ltd) | 35317 |
| amlodipine 5mg / valsartan 80mg tablets | 35329 |
| amlodipine 5mg / valsartan 160mg tablets | 35343 |
| hydrochlorothiazide with olmesartan medoxomil 12.5mg with 20mg tablet | 35380 |
| irbesartan 300mg / hydrochlorothiazide 25mg tablets | 35481 |
| cardioplen xl 2.5mg tablets (chiesi ltd) | 35592 |
| neozipine xl 60mg tablets (kent pharmaceuticals ltd) | 35646 |
| visken 5mg tablets (amco) | 35695 |
| kenzem sr 120mg capsules (kent pharmaceuticals ltd) | 35696 |
| exforge 5mg/160mg tablets (novartis pharmaceuticals uk ltd) | 35697 |
| verapamil 80mg tablets (teva uk ltd) | 35729 |
| perindopril erbumine 8mg tablets (a a h pharmaceuticals ltd) | 35731 |
| labrocol 100mg tablet (lagap) | 35778 |
| spironolactone 25mg tablet (celltech pharma europe ltd) | 35789 |
| enalapril 5mg tablets (a a h pharmaceuticals ltd) | 35794 |
| propranolol 80mg modified-release capsules (a a h pharmaceuticals ltd) | 35938 |
| celectol 400mg tablets (zentiva) | 35940 |
| cardozin xl 4mg tablet (hillcross pharmaceuticals ltd) | 36023 |
| amlodipine 10mg tablets (actavis uk ltd) | 36202 |
| atenolol 50mg tablets (tillomed laboratories ltd) | 36261 |
| propranolol 10mg tablets (generics (uk) ltd) | 36576 |
| zemret xl 180mg capsule (neo laboratories ltd) | 36583 |
| propranolol sr 160mg modified-release capsule (hillcross pharmaceuticals ltd) | 36603 |
| parmid xl 10mg tablets (sandoz ltd) | 36620 |
| aliskiren 150mg tablets | 36629 |
| hytrin 2mg tablets (amco) | 36649 |
| zemret xl 300mg capsule (neo laboratories ltd) | 36664 |
| slocinx xl 4mg tablets (zentiva) | 36740 |
| ednyt 10mg tablet (dominion pharma) | 36753 |
| bumetanide 1mg tablets (ivax pharmaceuticals uk ltd) | 36767 |
| hytrin 5mg tablets (amco) | 36780 |
| rasilez 150mg tablets (novartis pharmaceuticals uk ltd) | 36878 |
| rasilez 300mg tablets (novartis pharmaceuticals uk ltd) | 36879 |
| aliskiren 300mg tablets | 36909 |
| nifedipine 20mg modified-release tablets | 37025 |
| sitaxentan 100mg tablets | 37085 |
| bisoprolol 2.5mg tablets (a a h pharmaceuticals ltd) | 37118 |
| valni xl 30mg tablets (zentiva) | 37184 |
| cardozin xl 4mg tablet (teva uk ltd) | 37243 |
| triamterene with chlortalidone 50mg + 25mg tablet | 37294 |
| hytrin 10mg tablets (amco) | 37428 |
| neozipine xl 30mg tablets (kent pharmaceuticals ltd) | 37530 |
| valsartan 320mg tablets | 37573 |
| losartan 100mg / hydrochlorothiazide 12.5mg tablets | 37650 |
| captopril 25mg tablets (teva uk ltd) | 37655 |
| lisinopril 10mg / hydrochlorothiazide 12.5mg tablets (teva uk ltd) | 37710 |
| co-tenidone 100mg/25mg tablets (generics (uk) ltd) | 37725 |
| cozaar-comp 100mg/12.5mg tablets (merck sharp & dohme ltd) | 37747 |
| kenzem sr 60mg capsules (kent pharmaceuticals ltd) | 37774 |
| bisoprolol 2.5mg tablet (teva uk ltd) | 37837 |
| felotens xl 2.5mg tablets (genus pharmaceuticals ltd) | 37897 |
| coversyl arginine plus 5mg/1.25mg tablets (servier laboratories ltd) | 37908 |
| perindopril arginine 5mg tablets | 37930 |

| | |
|---|---|
| perindopril arginine 2.5mg tablets | 37964 |
| coversyl arginine 5mg tablets (servier laboratories ltd) | 37965 |
| perindopril arginine 10mg tablets | 37971 |
| perindopril arginine 5mg / indapamide 1.25mg tablets | 37978 |
| coversyl arginine 10mg tablets (servier laboratories ltd) | 38026 |
| coversyl arginine 2.5mg tablets (servier laboratories ltd) | 38034 |
| diltiazem hcl 60mg modified-release tablet (lagap) | 38066 |
| nifedipine sr 30mg tablet (hillcross pharmaceuticals ltd) | 38107 |
| perindopril erbumine 4mg tablets (teva uk ltd) | 38285 |
| ramipril 2.5/5mg/10mg tablet | 38308 |
| hydrochlorothiazide with losartan 12.5mg with 100mg tablet | 38367 |
| valsartan 80mg tablets | 38395 |
| keloc sr 10mg tablets (teva uk ltd) | 38434 |
| telmisartan 80mg / hydrochlorothiazide 25mg tablets | 38459 |
| cardozin xl 4mg tablets (arrow generics ltd) | 38461 |
| perindopril erbumine 4mg tablets (apotex uk ltd) | 38510 |
| tildiem la 200 capsules (sanofi) | 38545 |
| adizem-sr 90mg capsules (napp pharmaceuticals ltd) | 38632 |
| adizem-xl 300mg capsules (napp pharmaceuticals ltd) | 38634 |
| adizem-sr 120mg capsules (napp pharmaceuticals ltd) | 38818 |
| adizem-sr 180mg capsules (napp pharmaceuticals ltd) | 38831 |
| adizem-xl 180mg capsules (napp pharmaceuticals ltd) | 38855 |
| adizem-xl 120mg capsules (napp pharmaceuticals ltd) | 38865 |
| tildiem la 300 capsules (sanofi) | 38876 |
| adizem-xl 240mg capsules (napp pharmaceuticals ltd) | 38882 |
| micardisplus 80mg/25mg tablets (boehringer ingelheim ltd) | 38889 |
| quinil 10mg tablets (tillomed laboratories ltd) | 38899 |
| frumil ls 20mg/2.5mg tablets (sanofi) | 38901 |
| adizem-sr 120mg tablets (napp pharmaceuticals ltd) | 38964 |
| bisoprolol 7.5mg tablets (a a h pharmaceuticals ltd) | 38991 |
| zestoretic 20 tablets (astrazeneca uk ltd) | 38995 |
| verapamil 40mg tablets (teva uk ltd) | 39009 |
| hydrochlorothiazide with olmesartan medoxomil 25mg with 20mg tablet | 39021 |
| zestoretic 10 tablets (astrazeneca uk ltd) | 39137 |
| carace 20 plus tablets (merck sharp & dohme ltd) | 39147 |
| bi-carzem sr 60mg capsules (tillomed laboratories ltd) | 39171 |
| diovan 320mg tablets (novartis pharmaceuticals uk ltd) | 39199 |
| capozide ls 12.5mg/25mg tablets (bristol-myers squibb pharmaceuticals ltd) | 39227 |
| propranolol 80mg modified-release capsules (teva uk ltd) | 39233 |
| carace 10 plus tablets (merck sharp & dohme ltd) | 39242 |
| bi-carzem sr 90mg capsules (tillomed laboratories ltd) | 39298 |
| tritace 10mg tablet (sterwin medicines) | 39355 |
| neofel xl 2.5mg tablets (kent pharmaceuticals ltd) | 39357 |
| sotalol 80mg tablets (a a h pharmaceuticals ltd) | 39423 |
| varbim xl 1.5mg tablets (teva uk ltd) | 39447 |
| bumetanide 1mg tablets (actavis uk ltd) | 39602 |
| valni xl 60mg tablets (zentiva) | 39800 |
| amlodipine 5mg tablets (dr reddy's laboratories (uk) ltd) | 39804 |
| frumil 40mg/5mg tablets (sanofi) | 39807 |
| vivacor 5mg tablets (lexon (uk) ltd) | 39846 |
| amlodipine 5mg tablets (teva uk ltd) | 39914 |
| losartan 12.5mg tablets | 39944 |
| sevikar 20mg/5mg tablets (daiichi sankyo uk ltd) | 39984 |
| nifedipine 20mg capsule | 40074 |
| bendroflumethiazide 5mg tablets (ivax pharmaceuticals uk ltd) | 40149 |
| metoprolol 100mg tablets (ivax pharmaceuticals uk ltd) | 40167 |
| labetalol 400mg tablets (a a h pharmaceuticals ltd) | 40240 |
| propranolol la 160mg capsule (approved prescription services ltd) | 40241 |

| | |
|---|---|
| baratol 25mg tablets (amdipharm plc) | 40256 |
| moxonidine 200microgram tablets (teva uk ltd) | 40310 |
| olmesartan medoxomil 20mg / amlodipine 5mg tablets | 40316 |
| quinil 5mg tablets (tillomed laboratories ltd) | 40355 |
| ramipril 10mg tablets (a a h pharmaceuticals ltd) | 40384 |
| verapamil 120mg tablets (teva uk ltd) | 40405 |
| bendogen 50mg tablet (lagap) | 40421 |
| ambrisentan 10mg tablets | 40527 |
| ambrisentan 5mg tablets | 40528 |
| cozaar 12.5mg tablets (merck sharp & dohme ltd) | 40571 |
| vascalpha 5mg modified-release tablets (almus pharmaceuticals ltd) | 40633 |
| olmesartan medoxomil 40mg / amlodipine 5mg tablets | 40639 |
| olmesartan medoxomil 40mg / amlodipine 10mg tablets | 40668 |
| doxazosin 4mg tablets (teva uk ltd) | 40678 |
| nebivolol 2.5mg tablets | 40761 |
| bendroflumethiazide 2.5mg tablets (almus pharmaceuticals ltd) | 40886 |
| doxazosin 2mg tablets (ivax pharmaceuticals uk ltd) | 40891 |
| torasemide 5mg tablets (a a h pharmaceuticals ltd) | 40898 |
| thelin 100mg tablets (pfizer ltd) | 40899 |
| indapamide 2.5mg tablets (genus pharmaceuticals ltd) | 40907 |
| spironolactone 25mg tablets (almus pharmaceuticals ltd) | 41074 |
| sevikar 40mg/10mg tablets (daiichi sankyo uk ltd) | 41203 |
| sevikar 40mg/5mg tablets (daiichi sankyo uk ltd) | 41205 |
| furosemide 20mg tablets (wockhardt uk ltd) | 41292 |
| furosemide 500mg tablets (teva uk ltd) | 41405 |
| enalapril 2.5mg tablets (a a h pharmaceuticals ltd) | 41417 |
| bi-carzem sr 120mg capsules (tillomed laboratories ltd) | 41489 |
| bendroflumethiazide 5mg tablets (teva uk ltd) | 41517 |
| lisopress 20mg tablets (teva uk ltd) | 41522 |
| lisopress 5mg tablets (teva uk ltd) | 41532 |
| co-amilofruse 2.5mg/20mg tablets (teva uk ltd) | 41533 |
| lisopress 2.5mg tablets (teva uk ltd) | 41538 |
| doxazosin 1mg tablets (ivax pharmaceuticals uk ltd) | 41543 |
| propranolol 40mg tablets (a a h pharmaceuticals ltd) | 41555 |
| co-amilozide 5mg/50mg tablets (teva uk ltd) | 41556 |
| co-tenidone 100mg/25mg tablets (teva uk ltd) | 41572 |
| lisopress 10mg tablets (teva uk ltd) | 41573 |
| verapamil 80mg tablets (actavis uk ltd) | 41586 |
| bisoprolol 10mg tablets (teva uk ltd) | 41591 |
| spironolactone 100mg tablets (actavis uk ltd) | 41592 |
| captopril 25mg tablets (actavis uk ltd) | 41617 |
| amiloride 5mg tablet (ivax pharmaceuticals uk ltd) | 41630 |
| captopril 12.5mg tablets (actavis uk ltd) | 41633 |
| diltiazem 60mg modified-release tablets (ivax pharmaceuticals uk ltd) | 41635 |
| hydralazine 50mg tablets (actavis uk ltd) | 41639 |
| prazosin 500microgram tablet (approved prescription services ltd) | 41651 |
| prazosin 500microgram tablets (a a h pharmaceuticals ltd) | 41652 |
| spironolactone 100mg tablets (teva uk ltd) | 41660 |
| methyldopa 250mg tablet (c p pharmaceuticals ltd) | 41661 |
| verapamil 80mg tablets (ivax pharmaceuticals uk ltd) | 41679 |
| verapamil 120mg tablets (generics (uk) ltd) | 41693 |
| enalapril 2.5mg tablets (ivax pharmaceuticals uk ltd) | 41694 |
| spironolactone 50mg tablets (ivax pharmaceuticals uk ltd) | 41706 |
| co-amilofruse 5mg/40mg tablets (actavis uk ltd) | 41719 |
| prazosin 1mg tablets (a a h pharmaceuticals ltd) | 41721 |
| celiprolol 200mg tablets (teva uk ltd) | 41740 |
| captopril 50mg tablets (teva uk ltd) | 41743 |
| enalapril 10mg tablets (sandoz ltd) | 41746 |

| | |
|---|---|
| labetalol 100mg tablets (generics (uk) ltd) | 41827 |
| furosemide 500mg tablets (actavis uk ltd) | 41828 |
| tensaid xl 1.5mg tablets (generics (uk) ltd) | 41861 |
| ethibide xl 1.5mg tablets (genus pharmaceuticals ltd) | 41885 |
| adipine la 30mg modified-release tablet (chiesi ltd) | 41979 |
| tritace 1.25mg tablet (sterwin medicines) | 42081 |
| moduretic 5mg/50mg tablets (merck sharp & dohme ltd) | 42142 |
| amlodipine 10mg tablets (zentiva) | 42210 |
| quinil 40mg tablets (tillomed laboratories ltd) | 42285 |
| vera-til sr 120mg tablets (actavis uk ltd) | 42625 |
| pralenal 5 tablets (opus pharmaceuticals ltd) | 42723 |
| diltiazem sr 120mg capsule (hillcross pharmaceuticals ltd) | 42731 |
| celiprolol 200mg tablets (generics (uk) ltd) | 42795 |
| diltiazem hcl 180mg capsule (pliva pharma ltd) | 42804 |
| diltiazem xl 240mg capsule (hillcross pharmaceuticals ltd) | 42819 |
| enalapril 10mg tablets (teva uk ltd) | 42894 |
| enalapril 5mg tablets (teva uk ltd) | 42901 |
| enalapril 20mg tablets (teva uk ltd) | 42902 |
| indapamide 2.5mg tablets (niche generics ltd) | 42906 |
| enalapril 5mg tablets (ivax pharmaceuticals uk ltd) | 42908 |
| nifedipine 10mg capsules (teva uk ltd) | 42912 |
| mapemid xl 1.5mg tablets (teva uk ltd) | 43184 |
| valni 20 retard tablets (tillomed laboratories ltd) | 43222 |
| bisoprolol 1.25mg tablets (generics (uk) ltd) | 43251 |
| olmesartan medoxomil 40mg / hydrochlorothiazide 12.5mg tablets | 43322 |
| pinefeld xl 10mg tablets (tillomed laboratories ltd) | 43394 |
| nifedipine extra 60mg modified-release tablet | 43410 |
| enalapril 5mg tablets (sandoz ltd) | 43411 |
| lisinopril 2.5mg tablets (a a h pharmaceuticals ltd) | 43412 |
| lisinopril 20mg tablets (a a h pharmaceuticals ltd) | 43413 |
| lisinopril 10mg tablets (a a h pharmaceuticals ltd) | 43416 |
| lisinopril 5mg tablets (a a h pharmaceuticals ltd) | 43418 |
| diltiazem 120mg modified-release tablets (a a h pharmaceuticals ltd) | 43430 |
| captopril 6.25mg tablets | 43432 |
| amlodipine 5mg tablets (wockhardt uk ltd) | 43470 |
| hydralazine 25mg tablets (actavis uk ltd) | 43500 |
| captopril 25mg tablet (generics (uk) ltd) | 43507 |
| co-amilofruse 5mg/40mg tablets (sandoz ltd) | 43508 |
| nifedipine 10mg capsules (a a h pharmaceuticals ltd) | 43511 |
| felodipine 5mg modified-release tablets (a a h pharmaceuticals ltd) | 43512 |
| spironolactone 50mg tablets (a a h pharmaceuticals ltd) | 43514 |
| nifedipine 10mg capsules (actavis uk ltd) | 43515 |
| indapamide 2.5mg tablets (actavis uk ltd) | 43516 |
| amiloride 5mg tablets (generics (uk) ltd) | 43523 |
| propranolol 10mg tablets (ivax pharmaceuticals uk ltd) | 43525 |
| moxonidine 400microgram tablets (sandoz ltd) | 43531 |
| prazosin 500microgram tablets (ivax pharmaceuticals uk ltd) | 43547 |
| sotalol 40mg tablets (ivax pharmaceuticals uk ltd) | 43549 |
| enalapril 2.5mg tablets (zentiva) | 43563 |
| bisoprolol 5mg tablet (pliva pharma ltd) | 43564 |
| lisinopril 2.5mg tablets (sandoz ltd) | 43566 |
| captopril 25mg tablets (a a h pharmaceuticals ltd) | 43649 |
| colixil xl 4mg tablets (sandoz ltd) | 43695 |
| adalat la 30 tablets (bayer plc) | 43753 |
| vascalpha 10mg modified-release tablets (almus pharmaceuticals ltd) | 43790 |
| perindopril erbumine 2mg tablets (actavis uk ltd) | 43813 |
| adalat la 60 tablets (bayer plc) | 43818 |
| vera-til sr 240mg tablets (actavis uk ltd) | 43879 |

| | |
|---|---|
| amlodipine 5mg tablets (almus pharmaceuticals ltd) | 43880 |
| olmetec plus 40mg/12.5mg tablets (daiichi sankyo uk ltd) | 43915 |
| aldomet 250mg tablets (aspen pharma trading ltd) | 43988 |
| aldomet 500mg tablets (aspen pharma trading ltd) | 43989 |
| labetalol 200mg tablets (actavis uk ltd) | 44083 |
| indipam xl 1.5mg tablets (actavis uk ltd) | 44168 |
| zemret 240 xl capsules (tillomed laboratories ltd) | 44192 |
| amiloride 5.67mg tablets | 44254 |
| ednyt 2.5mg tablet (dominion pharma) | 44657 |
| valsartan 160mg tablets | 44778 |
| nebivolol 2.5mg tablets (a a h pharmaceuticals ltd) | 44808 |
| atenolol 25mg tablets (actavis uk ltd) | 44858 |
| felodipine sr 5mg tablet (approved prescription services ltd) | 44859 |
| bi-carzem xl 300mg capsule (tillomed laboratories ltd) | 44887 |
| larbex xl 4mg tablets (teva uk ltd) | 45040 |
| verapamil hc 240mg modified-release tablet (actavis uk ltd) | 45051 |
| enalapril 5mg tablets (kent pharmaceuticals ltd) | 45217 |
| captopril capsules | 45228 |
| labetalol 400mg tablets (sandoz ltd) | 45250 |
| ramipril 1.25mg capsules (actavis uk ltd) | 45264 |
| doxazosin sr 4mg tablet (generics (uk) ltd) | 45265 |
| amlodipine 5mg tablets (sandoz ltd) | 45279 |
| nicardipine 30mg capsules (a a h pharmaceuticals ltd) | 45292 |
| propranolol 40mg tablets (ivax pharmaceuticals uk ltd) | 45297 |
| lisinopril 10mg tablets (actavis uk ltd) | 45300 |
| bumetanide 1mg tablets (teva uk ltd) | 45305 |
| verapamil 240mg modified-release tablets (generics (uk) ltd) | 45308 |
| acebutolol 400mg tablets (a a h pharmaceuticals ltd) | 45309 |
| perindopril erbumine 2mg tablets (a a h pharmaceuticals ltd) | 45319 |
| lisinopril 20mg tablets (actavis uk ltd) | 45324 |
| doxazosin 1mg tablets (sandoz ltd) | 45328 |
| lisinopril 5mg tablets (actavis uk ltd) | 45337 |
| ramipril 10mg capsule (actavis uk ltd) | 45340 |
| doxazosin 4mg tablets (sandoz ltd) | 45342 |
| propranolol sr 80mg modified-release capsule (c p pharmaceuticals ltd) | 45343 |
| propranolol 10mg tablets (almus pharmaceuticals ltd) | 45494 |
| clonidine 25microgram tablets (a a h pharmaceuticals ltd) | 45578 |
| doxazosin 2mg tablets (dexcel-pharma ltd) | 45583 |
| diovan 160mg tablet (novartis pharmaceuticals uk ltd) | 45600 |
| adanif xl 30mg tablets (focus pharmaceuticals ltd) | 45685 |
| diltiazem hcl 240mg capsule (pliva pharma ltd) | 45759 |
| lisinopril 5mg tablets (almus pharmaceuticals ltd) | 45816 |
| beta-prograne 160mg modified-release capsules (teva uk ltd) | 45877 |
| hydroflumethiazide with spironolactone 50mg+50mg tablet | 45916 |
| perindopril erbumine 8mg tablets (teva uk ltd) | 45938 |
| verapamil 120mg tablets (kent pharmaceuticals ltd) | 46009 |
| cardozin xl 4mg tablets (almus pharmaceuticals ltd) | 46066 |
| neo-naclex 2.5mg tablets (amco) | 46302 |
| sevikar hct 20mg/5mg/12.5mg tablets (daiichi sankyo uk ltd) | 46355 |
| half beta-prograne 80mg modified-release capsules (teva uk ltd) | 46363 |
| quinil 20mg tablets (tillomed laboratories ltd) | 46365 |
| nifedipine 10mg capsules (ivax pharmaceuticals uk ltd) | 46445 |
| torasemide 5mg tablets (teva uk ltd) | 46525 |
| raporsin xl 4mg tablets (actavis uk ltd) | 46526 |
| lopresor 50mg tablets (recordati pharmaceuticals ltd) | 46614 |
| indapamide 1.5mg modified-release tablets (a a h pharmaceuticals ltd) | 46675 |
| olmesartan medoxomil with amlodipine and hydrochlorothiazide 20mg + 5mg + 12.5mg tablet | 46687 |
| furosemide 40mg tablets (almus pharmaceuticals ltd) | 46699 |

| | |
|---|---|
| olmesartan medoxomil with amlodipine and hydrochlorothiazide 40mg + 10mg + 12.5mg tablet | 46715 |
| lopresor 100mg tablets (recordati pharmaceuticals ltd) | 46740 |
| olmesartan medoxomil with amlodipine and hydrochlorothiazide 40mg + 5mg + 12.5mg tablet | 46792 |
| verapamil hc 240mg modified-release tablet (sandoz ltd) | 46884 |
| adanif xl 60mg tablets (focus pharmaceuticals ltd) | 46887 |
| atenolol 100mg tablets (kent pharmaceuticals ltd) | 46908 |
| co-amilozide 5mg/50mg tablets (a a h pharmaceuticals ltd) | 46916 |
| prazosin 1mg tablets (ivax pharmaceuticals uk ltd) | 46922 |
| amiloride 5mg tablets (wockhardt uk ltd) | 46930 |
| atenolol 100mg tablets (actavis uk ltd) | 46931 |
| carvedilol 3.125mg tablets (actavis uk ltd) | 46935 |
| carvedilol 3.125mg tablets (a a h pharmaceuticals ltd) | 46936 |
| diltiazem 60mg modified-release tablets (actavis uk ltd) | 46937 |
| furosemide 40mg tablets (arrow generics ltd) | 46948 |
| captopril 12.5mg tablets (a a h pharmaceuticals ltd) | 46951 |
| co-tenidone 100mg/25mg tablets (actavis uk ltd) | 46952 |
| verapamil 80mg tablets (generics (uk) ltd) | 46955 |
| captopril 12.5mg tablets (tillomed laboratories ltd) | 46957 |
| enalapril 5mg tablets (generics (uk) ltd) | 46974 |
| lisinopril 5mg tablets (sandoz ltd) | 46975 |
| lisinopril 20mg tablets (sandoz ltd) | 46979 |
| losartan 100mg tablets (teva uk ltd) | 47006 |
| nifedipine 10mg modified-release tablet (kent pharmaceuticals ltd) | 47027 |
| bisoprolol 2.5mg tablets (generics (uk) ltd) | 47041 |
| lisinopril 10mg tablets (almus pharmaceuticals ltd) | 47159 |
| adipine la 60mg modified-release tablet (chiesi ltd) | 47217 |
| verapamil 120mg modified-release tablets (a a h pharmaceuticals ltd) | 47222 |
| verapamil 240mg modified-release tablets (teva uk ltd) | 47230 |
| adcirca 20mg tablets (eli lilly and company ltd) | 47264 |
| nifedipine xl 60mg tablet (hillcross pharmaceuticals ltd) | 47285 |
| nebivolol 2.5mg tablets (glenmark generics (europe) ltd) | 47300 |
| lercanidipine 10mg tablets (generics (uk) ltd) | 47331 |
| diltiazem sr 60mg capsule (hillcross pharmaceuticals ltd) | 47415 |
| olmesartan medoxomil with amlodipine and hydrochlorothiazide 40mg + 5mg + 25mg tablet | 47467 |
| horizem sr 60mg capsules (horizon lifecare) | 47530 |
| half beta-prograne 80mg modified-release capsules (actavis uk ltd) | 47543 |
| sevikar hct 40mg/5mg/12.5mg tablets (daiichi sankyo uk ltd) | 47573 |
| zemret 300 xl capsules (tillomed laboratories ltd) | 47608 |
| nifedipine 30mg modified-release tablets (a a h pharmaceuticals ltd) | 47614 |
| sevikar hct 40mg/10mg/12.5mg tablets (daiichi sankyo uk ltd) | 47616 |
| tracleer 62.5mg tablets (actelion pharmaceuticals uk ltd) | 47654 |
| labetalol 400mg tablet (approved prescription services ltd) | 47673 |
| labetalol 200mg tablet (c p pharmaceuticals ltd) | 47674 |
| spiretic 25mg tablet (ddsa pharmaceuticals ltd) | 47687 |
| bi-carzem xl 240mg capsules (tillomed laboratories ltd) | 47724 |
| sevikar hct 40mg/5mg/25mg tablets (daiichi sankyo uk ltd) | 47727 |
| zemret 180 xl capsules (tillomed laboratories ltd) | 47732 |
| co-triamterzide 50mg/25mg tablets (a a h pharmaceuticals ltd) | 47804 |
| doxazosin xl 4mg tablet (hillcross pharmaceuticals ltd) | 47807 |
| furosemide 20mg tablet (celltech pharma europe ltd) | 47815 |
| bedranol sr 80mg capsules (almus pharmaceuticals ltd) | 47833 |
| bendroflumethiazide 2.5mg tablets (kent pharmaceuticals ltd) | 47844 |
| atenolol 25mg tablets (almus pharmaceuticals ltd) | 47870 |
| nimodrel xl 60mg tablets (zurich pharmaceuticals) | 47887 |
| bedranol sr 160mg capsules (almus pharmaceuticals ltd) | 47907 |
| ramipril 2.5mg capsules (actavis uk ltd) | 47998 |
| ramipril 5mg capsules (actavis uk ltd) | 48008 |

| | |
|---|---|
| felodipine 5mg modified-release tablet (sandoz ltd) | 48009 |
| losartan 100mg / hydrochlorothiazide 12.5mg tablets (teva uk ltd) | 48039 |
| perindopril erbumine 2mg tablets (generics (uk) ltd) | 48049 |
| ramipril 2.5mg capsules (almus pharmaceuticals ltd) | 48053 |
| indapamide 2.5mg tablets (zentiva) | 48079 |
| perindopril arginine 4mg with indapamide 1.25mg tablet | 48098 |
| indapamide 2.5mg tablets (a a h pharmaceuticals ltd) | 48099 |
| hydrochlorothiazide capsule | 48132 |
| doxazosin 1mg tablets (actavis uk ltd) | 48150 |
| perindopril erbumine 4mg tablets (sandoz ltd) | 48180 |
| perindopril erbumine 4mg tablets (actavis uk ltd) | 48214 |
| diltiazem 60mg modified-release capsules (alliance healthcare (distribution) ltd) | 48272 |
| diltiazem 90mg modified-release capsules (a a h pharmaceuticals ltd) | 48282 |
| diltiazem 120mg modified-release capsules (a a h pharmaceuticals ltd) | 48288 |
| losartan 25mg tablets (dexcel-pharma ltd) | 48398 |
| diltiazem 90mg modified-release capsules (alliance healthcare (distribution) ltd) | 48457 |
| adizem-sr 90mg capsules (de pharmaceuticals) | 48870 |
| diltiazem 120mg modified-release tablets (alliance healthcare (distribution) ltd) | 49001 |
| ramipril 10mg capsules (actavis uk ltd) | 49164 |
| diltiazem 120mg modified-release capsules (alliance healthcare (distribution) ltd) | 49289 |
| nifedipine 20mg modified-release tablets (alliance healthcare (distribution) ltd) | 49338 |
| diltiazem 90mg modified-release tablets (alliance healthcare (distribution) ltd) | 49390 |
| perindopril erbumine 2mg tablets (consilient health ltd) | 49491 |
| losartan 25mg tablets (generics (uk) ltd) | 49492 |
| indapamide 2.5mg tablets (phoenix healthcare distribution ltd) | 49529 |
| losartan 100mg tablets (a a h pharmaceuticals ltd) | 49588 |
| amlodipine 10mg tablets (de pharmaceuticals) | 49636 |
| nifedipine 10mg modified-release tablets (alliance healthcare (distribution) ltd) | 49762 |
| atenolol 25mg tablets (bristol laboratories ltd) | 49953 |
| candesartan 8mg tablets (teva uk ltd) | 50185 |
| congescor 2.5mg tablets (teva uk ltd) | 50224 |
| congescor 1.25mg tablets (teva uk ltd) | 50300 |
| coversyl arginine 5mg tablets (waymade healthcare plc) | 50347 |
| perindopril 2mg tablet (servier laboratories ltd) | 50402 |
| bisoprolol 1.25mg tablet (teva uk ltd) | 50403 |
| doxazosin 2mg tablets (alliance healthcare (distribution) ltd) | 50467 |
| bisoprolol 2.5mg tablets (chanelle medical uk ltd) | 50514 |
| atenolol 25mg tablets (alliance healthcare (distribution) ltd) | 50702 |
| losartan 25mg tablets (a a h pharmaceuticals ltd) | 50971 |
| candesartan 8mg tablets (de pharmaceuticals) | 51117 |
| losartan 25mg tablets (arrow generics ltd) | 51186 |
| coversyl arginine plus 5mg/1.25mg tablets (de pharmaceuticals) | 51258 |
| tildiem retard 120mg tablets (mawdsley-brooks & company ltd) | 51261 |
| azilsartan medoxomil 80mg tablets | 51368 |
| lisinopril 20mg tablets (tillomed laboratories ltd) | 51433 |
| securon sr 240mg tablets (waymade healthcare plc) | 51461 |
| candesartan 8mg tablets (a a h pharmaceuticals ltd) | 51519 |
| bisoprolol 1.25mg tablets (actavis uk ltd) | 51528 |
| losartan 50mg tablets (actavis uk ltd) | 51601 |
| candesartan 4mg tablets (mawdsley-brooks & company ltd) | 51647 |
| spironolactone 25mg tablets (de pharmaceuticals) | 51652 |
| doxazosin 4mg tablets (actavis uk ltd) | 51685 |
| ramipril 5mg capsules (bristol laboratories ltd) | 51701 |
| ramipril 2.5mg capsules (alliance healthcare (distribution) ltd) | 51714 |
| coversyl arginine 5mg tablets (de pharmaceuticals) | 51807 |
| edarbi 20mg tablets (takeda uk ltd) | 51897 |
| adalat la 60 tablets (sigma pharmaceuticals plc) | 51917 |
| atenolol 25mg tablets (co-pharma ltd) | 51998 |

| | |
|---|---|
| enalapril 10mg tablets (alliance healthcare (distribution) ltd) | 52010 |
| adalat la 30 tablets (mawdsley-brooks & company ltd) | 52017 |
| lisinopril 5mg tablets (phoenix healthcare distribution ltd) | 52088 |
| bedranol sr 160mg capsule (lagap) | 52136 |
| losartan 100mg / hydrochlorothiazide 25mg tablets (a a h pharmaceuticals ltd) | 52189 |
| ramipril 5mg capsules (sigma pharmaceuticals plc) | 52197 |
| candesartan 16mg tablets (a a h pharmaceuticals ltd) | 52208 |
| adizem-xl 180mg capsules (de pharmaceuticals) | 52276 |
| captopril 2mg capsules | 52293 |
| atenolol 25mg tablets (crescent pharma ltd) | 52310 |
| ramipril 1.25mg capsules (kent pharmaceuticals ltd) | 52399 |
| ramipril 10mg capsules (kent pharmaceuticals ltd) | 52407 |
| cozaar 100mg tablets (necessity supplies ltd) | 52427 |
| atenolol 50mg tablets (almus pharmaceuticals ltd) | 52500 |
| bisoprolol 1.25mg tablets (almus pharmaceuticals ltd) | 52548 |
| candesartan 8mg tablets (zentiva) | 52559 |
| inderal la 160mg capsules (sigma pharmaceuticals plc) | 52609 |
| bisoprolol 5mg tablets (alliance healthcare (distribution) ltd) | 52635 |
| tildiem la 200 capsules (mawdsley-brooks & company ltd) | 52701 |
| beta-adalat modified-release capsules (lexon (uk) ltd) | 52728 |
| propranolol 40mg tablets (kent pharmaceuticals ltd) | 52777 |
| co-diovan 80mg/12.5mg tablets (sigma pharmaceuticals plc) | 52858 |
| losartan 12.5mg tablets (a a h pharmaceuticals ltd) | 52886 |
| irbesartan 300mg tablets (sigma pharmaceuticals plc) | 52972 |
| doxzogen xl 4mg tablets (generics (uk) ltd) | 53033 |
| perindopril erbumine 8mg tablets (sandoz ltd) | 53058 |
| atenolol 50mg tablets (alliance healthcare (distribution) ltd) | 53204 |
| atenolol 50mg tablets (bristol laboratories ltd) | 53215 |
| sevikar hct 40mg/10mg/25mg tablets (daiichi sankyo uk ltd) | 53220 |
| lisinopril 10mg tablets (alliance healthcare (distribution) ltd) | 53271 |
| adalat la 30 tablets (necessity supplies ltd) | 53278 |
| doxazosin 4mg tablets (bristol laboratories ltd) | 53322 |
| bisoprolol 10mg tablets (a a h pharmaceuticals ltd) | 53334 |
| atenolol 50mg tablets (accord healthcare ltd) | 53414 |
| adalat la 30 tablets (de pharmaceuticals) | 53500 |
| lisinopril 20mg tablets (phoenix healthcare distribution ltd) | 53551 |
| ramipril 10mg tablets (alliance healthcare (distribution) ltd) | 53612 |
| ramipril 2.5mg capsules (bristol laboratories ltd) | 53621 |
| adalat retard 20mg tablets (lexon (uk) ltd) | 53629 |
| bisoprolol 2.5mg tablets (sandoz ltd) | 53664 |
| metolazone 2.5mg tablets | 53674 |
| candesartan 16mg tablets (teva uk ltd) | 53680 |
| enalapril 20mg tablets (alliance healthcare (distribution) ltd) | 53719 |
| candesartan 4mg tablets (teva uk ltd) | 53755 |
| atenolol 25mg tablets (sigma pharmaceuticals plc) | 53802 |
| lisinopril 5mg tablets (arrow generics ltd) | 53820 |
| atenolol 25mg tablets (boston healthcare ltd) | 53826 |
| valsartan 160mg capsules (generics (uk) ltd) | 53833 |
| amlodipine 5mg tablets (actavis uk ltd) | 53868 |
| bisoprolol 1.25mg tablets (a a h pharmaceuticals ltd) | 53885 |
| enalapril 5mg tablets (dexcel-pharma ltd) | 53915 |
| bisoprolol 2.5mg tablets (almus pharmaceuticals ltd) | 53916 |
| furosemide 20mg tablets (bristol laboratories ltd) | 53967 |
| lisinopril 10mg tablets (relonchem ltd) | 54037 |
| losartan 50mg tablets (accord healthcare ltd) | 54049 |
| losartan 50mg tablets (teva uk ltd) | 54057 |
| lisinopril 20mg / hydrochlorothiazide 12.5mg tablets (almus pharmaceuticals ltd) | 54201 |
| lisinopril 10mg tablets (arrow generics ltd) | 54288 |

| | |
|---|---|
| ramipril 2.5mg capsules (arrow generics ltd) | 54298 |
| indapamide 2.5mg tablets (alliance healthcare (distribution) ltd) | 54316 |
| candesartan 32mg tablets (teva uk ltd) | 54326 |
| trandolapril 4mg capsules (arrow generics ltd) | 54345 |
| losartan 100mg tablets (actavis uk ltd) | 54404 |
| candesartan 16mg tablets (consilient health ltd) | 54414 |
| bisoprolol 1.25mg tablets (alliance healthcare (distribution) ltd) | 54479 |
| nebivolol 2.5mg tablets (sigma pharmaceuticals plc) | 54487 |
| amlodipine 10mg tablets (alliance healthcare (distribution) ltd) | 54515 |
| atenolol 25mg tablets (zanza laboratories ltd) | 54542 |
| ramipril 2.5mg capsules (sigma pharmaceuticals plc) | 54620 |
| beta-prograne 160mg modified-release capsules (actavis uk ltd) | 54623 |
| amlodipine 5mg tablets (bristol laboratories ltd) | 54633 |
| amlodipine 10mg tablets (almus pharmaceuticals ltd) | 54654 |
| chlorothiazide 250mg tablets | 54679 |
| amlodipine 10mg tablets (sandoz ltd) | 54696 |
| valsartan 40mg capsules (teva uk ltd) | 54726 |
| perindopril erbumine 8mg tablets (consilient health ltd) | 54733 |
| losartan 50mg tablets (alliance healthcare (distribution) ltd) | 54735 |
| losartan 25mg tablets (actavis uk ltd) | 54740 |
| atenolol 50mg tablets (co-pharma ltd) | 54752 |
| doxazosin 4mg tablets (medreich plc) | 54785 |
| tildiem la 300 capsules (mawdsley-brooks & company ltd) | 54799 |
| furosemide 20mg tablets (sigma pharmaceuticals plc) | 54825 |
| losartan 50mg tablets (dexcel-pharma ltd) | 54843 |
| perindopril erbumine 2mg tablets (teva uk ltd) | 54899 |
| lisinopril 10mg tablets (bristol laboratories ltd) | 54928 |
| ramipril 5mg capsules (alliance healthcare (distribution) ltd) | 54941 |
| perindopril erbumine 8mg tablets (generics (uk) ltd) | 54942 |
| lisinopril 20mg tablets (accord healthcare ltd) | 55002 |
| irbesartan 300mg tablets (accord healthcare ltd) | 55017 |
| cozaar-comp 50mg/12.5mg tablets (sigma pharmaceuticals plc) | 55160 |
| valsartan 160mg capsules (arrow generics ltd) | 55187 |
| propranolol 40mg tablets (boston healthcare ltd) | 55228 |
| indapamide 2.5mg tablets (kent pharmaceuticals ltd) | 55259 |
| losartan 50mg tablets (generics (uk) ltd) | 55296 |
| bisoprolol 10mg tablets (sigma pharmaceuticals plc) | 55298 |
| ramipril 1.25mg capsules (a a h pharmaceuticals ltd) | 55299 |
| folpik xl 5mg tablets (teva uk ltd) | 55306 |
| lisinopril 20mg / hydrochlorothiazide 12.5mg tablets (a a h pharmaceuticals ltd) | 55399 |
| propranolol 40mg tablets (almus pharmaceuticals ltd) | 55416 |
| losartan 100mg tablets (bristol laboratories ltd) | 55446 |
| nifedipine 10mg capsules (co-pharma ltd) | 55455 |
| lisinopril 5mg tablets (alliance healthcare (distribution) ltd) | 55456 |
| bumetanide 1mg tablets (alliance healthcare (distribution) ltd) | 55548 |
| lisinopril 20mg tablets (sigma pharmaceuticals plc) | 55588 |
| lisinopril 10mg tablets (accord healthcare ltd) | 55639 |
| losartan 25mg tablets (phoenix healthcare distribution ltd) | 55718 |
| neofel xl 2.5mg tablets (actavis uk ltd) | 55740 |
| atenolol 50mg tablets (phoenix healthcare distribution ltd) | 55778 |
| bisoprolol 3.75mg tablets (actavis uk ltd) | 55791 |
| ramipril 5mg capsules (waymade healthcare plc) | 55798 |
| valsartan 160mg capsules (teva uk ltd) | 55821 |
| prazosin 5mg tablets (a a h pharmaceuticals ltd) | 55826 |
| propranolol 160mg tablets (generics (uk) ltd) | 55849 |
| pindolol 15mg tablet (hillcross pharmaceuticals ltd) | 55853 |
| lisinopril 2.5mg tablets (actavis uk ltd) | 55896 |
| enalapril 10mg tablets (dexcel-pharma ltd) | 55903 |

| | |
|---|---|
| doxazosin 1mg tablets (dexcel-pharma ltd) | 55906 |
| doxazosin 1mg tablets (alliance healthcare (distribution) ltd) | 55916 |
| bisoprolol 5mg tablets (accord healthcare ltd) | 55929 |
| ramipril 2.5mg capsules (waymade healthcare plc) | 56013 |
| ramipril 10mg tablets (pfizer ltd) | 56038 |
| furosemide 20mg tablets (kent pharmaceuticals ltd) | 56051 |
| losartan 50mg tablets (a a h pharmaceuticals ltd) | 56104 |
| ramipril 5mg capsules (kent pharmaceuticals ltd) | 56129 |
| doxazosin 2mg tablets (actavis uk ltd) | 56145 |
| amlodipine 10mg tablets (accord healthcare ltd) | 56147 |
| ramipril 1.25mg tablets (kent pharmaceuticals ltd) | 56148 |
| perindopril erbumine 4mg tablets (consilient health ltd) | 56162 |
| ramipril 10mg capsules (arrow generics ltd) | 56169 |
| half beta-prograne 80mg modified-release capsules (actavis uk ltd) | 56173 |
| losartan 50mg / hydrochlorothiazide 12.5mg tablets (actavis uk ltd) | 56204 |
| bisoprolol 3.75mg tablets (sandoz ltd) | 56240 |
| lisinopril 20mg / hydrochlorothiazide 12.5mg tablets (tillomed laboratories ltd) | 56244 |
| indapamide 2.5mg tablets (boston healthcare ltd) | 56296 |
| amlodipine 10mg tablets (bristol laboratories ltd) | 56334 |
| ramipril 10mg capsules (alliance healthcare (distribution) ltd) | 56356 |
| furosemide 40mg tablets (accord healthcare ltd) | 56375 |
| bisoprolol 2.5mg tablets (accord healthcare ltd) | 56459 |
| tildiem 60mg modified-release tablets (de pharmaceuticals) | 56467 |
| adalat la 60 tablets (necessity supplies ltd) | 56469 |
| perindopril erbumine 4mg tablets (kent pharmaceuticals ltd) | 56472 |
| perindopril erbumine 2mg tablets (sigma pharmaceuticals plc) | 56473 |
| celectol 200mg tablets (waymade healthcare plc) | 56485 |
| monocor 10mg tablets (dowelhurst ltd) | 56486 |
| zestril 5mg tablets (lexon (uk) ltd) | 56505 |
| coversyl 2mg tablets (dowelhurst ltd) | 56506 |
| coversyl 4mg tablets (dowelhurst ltd) | 56508 |
| capoten 12.5mg tablets (dowelhurst ltd) | 56509 |
| zestril 20mg tablets (sigma pharmaceuticals plc) | 56510 |
| perindopril erbumine 2mg tablets (sandoz ltd) | 56516 |
| azilsartan medoxomil 40mg tablets | 56606 |
| ramipril 1.25mg capsules (alliance healthcare (distribution) ltd) | 56704 |
| diltiazem 90mg modified-release capsules (cubic pharmaceuticals ltd) | 56758 |
| indapamide 2.5mg tablets (co-pharma ltd) | 56760 |
| ramipril 10mg capsules (phoenix healthcare distribution ltd) | 56763 |
| propranolol 40mg tablets (waymade healthcare plc) | 56764 |
| lercanidipine 20mg tablets (generics (uk) ltd) | 56767 |
| bisoprolol 2.5mg tablets (niche generics ltd) | 56768 |
| ecopace 12.5mg tablets (amco) | 56850 |
| ramipril 10mg capsules (sigma pharmaceuticals plc) | 56855 |
| losartan 100mg tablets (pfizer ltd) | 56970 |
| losartan 50mg / hydrochlorothiazide 12.5mg tablets (a a h pharmaceuticals ltd) | 56975 |
| lacidipine 4mg tablets (teva uk ltd) | 56994 |
| bisoprolol 2.5mg tablets (almus pharmaceuticals ltd) | 57023 |
| candesartan 8mg tablets (waymade healthcare plc) | 57026 |
| losartan 100mg tablets (generics (uk) ltd) | 57028 |
| lisinopril 10mg tablets (zentiva) | 57048 |
| bedranol sr 80mg capsules (almus pharmaceuticals ltd) | 57063 |
| ramipril 1.25mg capsules (waymade healthcare plc) | 57073 |
| doxazosin 2mg tablets (sigma pharmaceuticals plc) | 57074 |
| terazosin 2mg tablets (a a h pharmaceuticals ltd) | 57145 |
| bisoprolol 10mg tablets (accord healthcare ltd) | 57176 |
| diltiazem 120mg modified-release capsules (cubic pharmaceuticals ltd) | 57208 |
| ramipril 1.25mg tablets (sandoz ltd) | 57235 |

| | |
|---|---|
| candesartan 2mg tablets (actavis uk ltd) | 57266 |
| candesartan 8mg tablets (actavis uk ltd) | 57273 |
| perindopril tosilate 5mg tablets | 57333 |
| propranolol 40mg tablets (phoenix healthcare distribution ltd) | 57342 |
| ramipril 10mg capsules (waymade healthcare plc) | 57346 |
| lercanidipine 10mg tablets (aptil pharma ltd) | 57444 |
| doxazosin 4mg tablets (a a h pharmaceuticals ltd) | 57448 |
| adalat la 60 tablets (waymade healthcare plc) | 57531 |
| zestoretic 10 tablets (sigma pharmaceuticals plc) | 57539 |
| celectol 200mg tablets (dowelhurst ltd) | 57573 |
| cardicor 2.5mg tablets (necessity supplies ltd) | 57578 |
| zestril 2.5mg tablets (mawdsley-brooks & company ltd) | 57588 |
| tildiem 60mg modified-release tablets (waymade healthcare plc) | 57594 |
| adalat la 20 tablets (sigma pharmaceuticals plc) | 57653 |
| ramipril 1.25mg tablets (a a h pharmaceuticals ltd) | 57658 |
| lacidipine 4mg tablets (a a h pharmaceuticals ltd) | 57680 |
| perindopril erbumine 8mg tablets (actavis uk ltd) | 57701 |
| cozaar-comp 50mg/12.5mg tablets (de pharmaceuticals) | 57796 |
| perindopril erbumine 4mg tablets (glenmark generics (europe) ltd) | 57801 |
| atenolol 50mg tablets (zentiva) | 57817 |
| diltiazem 90mg modified-release tablets (cubic pharmaceuticals ltd) | 57859 |
| ramipril 5mg tablets (sigma pharmaceuticals plc) | 57864 |
| co-amilofruse 5mg/40mg tablets (kent pharmaceuticals ltd) | 57908 |
| bisoprolol 5mg tablets (sandoz ltd) | 57934 |
| candesartan 16mg tablets (alliance healthcare (distribution) ltd) | 57977 |
| irbesartan 150mg tablets (a a h pharmaceuticals ltd) | 58108 |
| irbesartan 150mg tablets (actavis uk ltd) | 58201 |
| losartan 25mg tablets (accord healthcare ltd) | 58274 |
| doxazosin 2mg tablets (medreich plc) | 58276 |
| lisinopril 5mg tablets (accord healthcare ltd) | 58294 |
| propranolol 10mg tablets (kent pharmaceuticals ltd) | 58297 |
| doxazosin 4mg tablets (phoenix healthcare distribution ltd) | 58325 |
| neofel xl 2.5mg tablets (almus pharmaceuticals ltd) | 58339 |
| propranolol 80mg tablets (teva uk ltd) | 58407 |
| lisinopril 2.5mg tablets (almus pharmaceuticals ltd) | 58451 |
| bisoprolol 7.5mg tablets (sandoz ltd) | 58455 |
| lisinopril 2.5mg tablets (kent pharmaceuticals ltd) | 58461 |
| propranolol 40mg tablets (alliance healthcare (distribution) ltd) | 58491 |
| bisoprolol 2.5mg tablets (medreich plc) | 58498 |
| bisoprolol 1.25mg tablets (sandoz ltd) | 58511 |
| adalat la 20 tablets (necessity supplies ltd) | 58557 |
| amlodipine 10mg tablets (apc pharmaceuticals & chemicals (europe) ltd) | 58580 |
| tracleer 125mg tablets (actelion pharmaceuticals uk ltd) | 58632 |
| candesartan 4mg tablets (actavis uk ltd) | 58646 |
| losartan 25mg tablets (bristol laboratories ltd) | 58649 |
| valsartan 40mg capsules (teva uk ltd) | 58669 |
| lisinopril 2.5mg tablets (generics (uk) ltd) | 58682 |
| bisoprolol 2.5mg tablets (waymade healthcare plc) | 58763 |
| perindopril erbumine 2mg tablets (kent pharmaceuticals ltd) | 58843 |
| lisinopril 10mg tablets (phoenix healthcare distribution ltd) | 58863 |
| lisinopril 10mg tablets (waymade healthcare plc) | 58871 |
| perindopril erbumine 2mg tablets (somex pharma) | 58874 |
| valsartan 80mg capsules (sigma pharmaceuticals plc) | 58910 |
| losartan 12.5mg tablets (alliance healthcare (distribution) ltd) | 58967 |
| bisoprolol 10mg tablets (niche generics ltd) | 58973 |
| bisoprolol 2.5mg tablets (alliance healthcare (distribution) ltd) | 58974 |
| bisoprolol 10mg tablets (medreich plc) | 58982 |
| nifedipine 10mg modified-release tablets (cubic pharmaceuticals ltd) | 58990 |

| | |
|---|---|
| amlodipine 10mg tablets (generics (uk) ltd) | 59001 |
| bisoprolol 5mg tablets (a a h pharmaceuticals ltd) | 59037 |
| losartan 25mg tablets (wockhardt uk ltd) | 59086 |
| dilzem xl 180 capsules (lexon (uk) ltd) | 59098 |
| lisinopril 5mg tablets (tillomed laboratories ltd) | 59109 |
| lisinopril 20mg tablets (alliance healthcare (distribution) ltd) | 59111 |
| bisoprolol 2.5mg tablets (zentiva) | 59148 |
| nifedipine 20mg modified-release tablets (cubic pharmaceuticals ltd) | 59163 |
| doxazosin 1mg tablets (kent pharmaceuticals ltd) | 59209 |
| lercanidipine 20mg tablets (actavis uk ltd) | 59233 |
| securon sr 240mg tablets (de pharmaceuticals) | 59264 |
| losartan 25mg tablets (sandoz ltd) | 59271 |
| furosemide 20mg tablets (alliance healthcare (distribution) ltd) | 59290 |
| losartan 12.5mg tablets (dexcel-pharma ltd) | 59340 |
| losartan 50mg tablets (pfizer ltd) | 59351 |
| irbesartan 300mg tablets (sandoz ltd) | 59393 |
| co-amilofruse 5mg/40mg tablets (waymade healthcare plc) | 59412 |
| propranolol 40mg tablets (accord healthcare ltd) | 59415 |
| valsartan 80mg capsules (a a h pharmaceuticals ltd) | 59448 |
| bisoprolol 1.25mg tablets (teva uk ltd) | 59495 |
| ramipril 2.5mg capsules (kent pharmaceuticals ltd) | 59557 |
| uard 120xl capsules (ennogen healthcare ltd) | 59585 |
| propranolol 160mg modified-release capsules (a a h pharmaceuticals ltd) | 59597 |
| ramipril 2.5mg capsules (phoenix healthcare distribution ltd) | 59603 |
| rawel xl 1.5mg tablets (consilient health ltd) | 59616 |
| candesartan 8mg tablets (consilient health ltd) | 59690 |
| atenolol 50mg tablets (boston healthcare ltd) | 59695 |
| losartan 50mg tablets (aptil pharma ltd) | 59750 |
| amlodipine 10mg tablets (teva uk ltd) | 59762 |
| perindopril erbumine 4mg tablets (aurobindo pharma ltd) | 59770 |
| ramipril 10mg capsules (bristol laboratories ltd) | 59788 |
| perindopril erbumine 8mg tablets (accord healthcare ltd) | 59790 |
| candesartan 2mg tablets (teva uk ltd) | 59802 |
| doxazosin 4mg tablets (dexcel-pharma ltd) | 59862 |
| dilzem xl 240 capsules (lexon (uk) ltd) | 59863 |
| furosemide 20mg tablets (phoenix healthcare distribution ltd) | 59884 |
| furosemide 40mg tablets (alliance healthcare (distribution) ltd) | 59911 |
| nebivolol 10mg tablets | 59961 |
| bisoprolol 5mg tablets (almus pharmaceuticals ltd) | 59969 |
| perindopril erbumine 2mg tablets (alliance healthcare (distribution) ltd) | 59972 |
| atenolol 25mg tablets (accord healthcare ltd) | 59982 |
| enalapril 20mg tablets (milpharm ltd) | 59996 |
| lisinopril 10mg tablets (kent pharmaceuticals ltd) | 60010 |
| indapamide 1.5mg modified-release tablets (waymade healthcare plc) | 60020 |
| perindopril erbumine 4mg tablets (sigma pharmaceuticals plc) | 60065 |
| perindopril erbumine 4mg / amlodipine 5mg tablets | 60067 |
| valsartan 160mg capsules (waymade healthcare plc) | 60076 |
| clonidine 25microgram tablets (waymade healthcare plc) | 60089 |
| lisinopril 2.5mg tablets (zentiva) | 60097 |
| enalapril 5mg tablets (medreich plc) | 60143 |
| doxazosin 4mg tablets (de pharmaceuticals) | 60200 |
| lisinopril 5mg tablets (zentiva) | 60232 |
| amlodipine 10mg tablets (phoenix healthcare distribution ltd) | 60244 |
| co-amilofruse 2.5mg/20mg tablets (aurobindo pharma ltd) | 60258 |
| furosemide 40mg tablets (amco) | 60291 |
| lisinopril 5mg tablets (relonchem ltd) | 60309 |
| prazosin 1mg tablet (approved prescription services ltd) | 60316 |
| doxazosin 1mg tablets (bristol laboratories ltd) | 60319 |

| | |
|---|---|
| spironolactone 25mg tablets (kent pharmaceuticals ltd) | 60343 |
| co-amilozide 2.5mg/25mg tablets (kent pharmaceuticals ltd) | 60354 |
| dilzem xl 180 capsules (sigma pharmaceuticals plc) | 60415 |
| bisoprolol 3.75mg tablets (de pharmaceuticals) | 60502 |
| losartan 100mg tablets (dexcel-pharma ltd) | 60506 |
| propranolol 40mg tablets (ranbaxy (uk) ltd) | 60565 |
| felodipine 2.5mg modified-release tablets (waymade healthcare plc) | 60569 |
| irbesartan 150mg tablets (teva uk ltd) | 60597 |
| adizem-xl 240mg capsules (waymade healthcare plc) | 60620 |
| parmid xl 2.5mg tablets (sandoz ltd) | 60652 |
| perindopril erbumine 4mg / amlodipine 10mg tablets | 60684 |
| lacidipine 2mg tablets (sigma pharmaceuticals plc) | 60699 |
| ramipril 5mg capsules (phoenix healthcare distribution ltd) | 60730 |
| trandolapril 500microgram capsules (teva uk ltd) | 60757 |
| bisoprolol 1.25mg tablets (medreich plc) | 60761 |
| nifedipine 10mg modified-release tablets (sigma pharmaceuticals plc) | 60856 |
| felodipine 2.5mg modified-release tablets (phoenix healthcare distribution ltd) | 60884 |
| bisoprolol 5mg tablets (medreich plc) | 60896 |
| moxonidine 200microgram tablets (generics (uk) ltd) | 60898 |
| propranolol 80mg modified-release capsules (kent pharmaceuticals ltd) | 60934 |
| diltiazem 120mg modified-release tablets (cubic pharmaceuticals ltd) | 61010 |
| physiotens 300microgram tablets (actavis uk ltd) | 61036 |
| losartan 100mg tablets (alliance healthcare (distribution) ltd) | 61053 |
| doxazosin 2mg tablets (bristol laboratories ltd) | 61066 |
| ramipril 5mg capsules (almus pharmaceuticals ltd) | 61067 |
| enalapril 10mg tablets (phoenix healthcare distribution ltd) | 61133 |
| telmisartan 20mg tablets (sigma pharmaceuticals plc) | 61177 |
| diltiazem 60mg modified-release capsules (sigma pharmaceuticals plc) | 61245 |
| lisinopril 20mg tablets (bristol laboratories ltd) | 61262 |
| perindopril erbumine 4mg tablets (accord healthcare ltd) | 61270 |
| doxazosin 4mg tablets (alliance healthcare (distribution) ltd) | 61283 |
| losartan 100mg tablets (accord healthcare ltd) | 61288 |
| quinapril 40mg tablets (generics (uk) ltd) | 61292 |
| ramipril 10mg capsules (almus pharmaceuticals ltd) | 61339 |
| bisoprolol 5mg tablets (de pharmaceuticals) | 61340 |
| amlodipine 5mg tablets (accord healthcare ltd) | 61422 |
| valsartan 160mg capsules (teva uk ltd) | 61442 |
| furosemide 20mg tablets (de pharmaceuticals) | 61475 |
| losartan 25mg tablets (aptil pharma ltd) | 61495 |
| ramipril 2.5mg tablets (actavis uk ltd) | 61499 |
| diltiazem 120mg modified-release capsules (sigma pharmaceuticals plc) | 61532 |
| bisoprolol 3.75mg tablets (waymade healthcare plc) | 61564 |
| lercanidipine 10mg tablets (de pharmaceuticals) | 61611 |
| bisoprolol 7.5mg tablets (almus pharmaceuticals ltd) | 61651 |
| carvedilol 3.125mg tablets (teva uk ltd) | 61663 |
| perindopril erbumine 8mg tablets (aurobindo pharma ltd) | 61693 |
| ramipril 5mg tablets (zentiva) | 61694 |
| clonidine 25microgram tablets (teva uk ltd) | 61710 |
| beta-adalat modified-release capsules (waymade healthcare plc) | 61719 |
| propranolol 10mg tablets (accord healthcare ltd) | 61727 |
| irbesartan 300mg tablets (teva uk ltd) | 61781 |
| zaroxolyn 2.5mg tablets (idis) | 61846 |
| ramipril 1.25mg tablets (teva uk ltd) | 61985 |
| doxazosin 1mg tablets (almus pharmaceuticals ltd) | 62019 |
| bumetanide 5mg tablets (a a h pharmaceuticals ltd) | 62024 |
| candesartan 16mg tablets (waymade healthcare plc) | 62035 |
| ramipril 5mg tablets (waymade healthcare plc) | 62036 |
| ramipril 1.25mg tablets (zentiva) | 62039 |

| | |
|---|---|
| diltiazem 120mg modified-release tablets (mawdsley-brooks & company ltd) | 62064 |
| diltiazem 90mg modified-release tablets (colorama pharmaceuticals ltd) | 62065 |
| cardide sr 1.5mg tablets (teva uk ltd) | 62066 |
| candesartan 4mg tablets (sandoz ltd) | 62140 |
| doxazosin 4mg tablets (almus pharmaceuticals ltd) | 62158 |
| adizem-sr 120mg capsules (waymade healthcare plc) | 62207 |
| co-amilozide 5mg/50mg tablets (alliance healthcare (distribution) ltd) | 62249 |
| atenolol 25mg tablets (waymade healthcare plc) | 62325 |
| irbesartan 300mg / hydrochlorothiazide 12.5mg tablets (actavis uk ltd) | 62337 |
| doxazosin 2mg tablets (phoenix healthcare distribution ltd) | 62351 |
| bisoprolol 1.25mg tablets (chanelle medical uk ltd) | 62361 |
| actelsar hct 80mg/12.5mg tablets (actavis uk ltd) | 62376 |
| losartan 12.5mg tablets (de pharmaceuticals) | 62388 |
| irbesartan 300mg tablets (a a h pharmaceuticals ltd) | 62415 |
| methyldopa 250mg tablets (sovereign medical ltd) | 62513 |
| hydrochlorothiazide 12.5mg tablets | 62516 |
| co-tenidone 100mg/25mg tablets (de pharmaceuticals) | 62537 |
| verapamil 80mg tablets (alliance healthcare (distribution) ltd) | 62552 |
| labetalol 100mg tablets (actavis uk ltd) | 62638 |
| co-amilozide 5mg/50mg tablets (phoenix healthcare distribution ltd) | 62700 |
| propranolol 80mg modified-release capsules (waymade healthcare plc) | 62711 |
| indapamide 1.5mg modified-release tablets (de pharmaceuticals) | 62771 |
| moxonidine 200microgram tablets (a a h pharmaceuticals ltd) | 62853 |
| enalapril 5mg tablets (de pharmaceuticals) | 62860 |
| losartan 50mg / hydrochlorothiazide 12.5mg tablets (teva uk ltd) | 62911 |
| diltiazem 120mg modified-release capsules (am distributions (yorkshire) ltd) | 62912 |
| ramipril 5mg tablets (teva uk ltd) | 62958 |
| ramipril 10mg tablets (phoenix healthcare distribution ltd) | 63010 |
| lisinopril 10mg tablets (de pharmaceuticals) | 63030 |
| nifedipine 10mg capsules (generics (uk) ltd) | 63041 |
| doxazosin 2mg tablets (almus pharmaceuticals ltd) | 63158 |
| losartan 25mg tablets (pfizer ltd) | 63222 |
| furosemide 20mg tablets (boston healthcare ltd) | 63237 |
| nifedipine 10mg modified-release tablets (am distributions (yorkshire) ltd) | 63246 |
| doxazosin 1mg tablets (sovereign medical ltd) | 63314 |
| enalapril 10mg tablets (almus pharmaceuticals ltd) | 63322 |
| folpik xl 2.5mg tablets (teva uk ltd) | 63331 |
| eprosartan 600mg tablets (a a h pharmaceuticals ltd) | 63337 |
| sabervel 75mg tablets (aspire pharma ltd) | 63385 |
| irbesartan 300mg tablets (alliance healthcare (distribution) ltd) | 63411 |
| carvedilol 12.5mg tablets (waymade healthcare plc) | 63422 |
| ramipril 2.5mg tablets (teva uk ltd) | 63442 |
| bisoprolol 2.5mg tablets (actavis uk ltd) | 63493 |
| amlodipine 10mg tablets (dr reddy's laboratories (uk) ltd) | 63515 |
| bisoprolol 5mg tablets (relonchem ltd) | 63535 |
| bumetanide 1mg tablets (phoenix healthcare distribution ltd) | 63555 |
| lisinopril 20mg tablets (kent pharmaceuticals ltd) | 63559 |
| irbesartan 300mg tablets (de pharmaceuticals) | 63717 |
| metoprolol 100mg tablets (waymade healthcare plc) | 63724 |
| labetalol 100mg tablets (waymade healthcare plc) | 63736 |
| macitentan 10mg tablets | 63780 |
| bisoprolol 2.5mg tablets (teva uk ltd) | 63850 |
| micardisplus 80mg/12.5mg tablets (waymade healthcare plc) | 63890 |
| lercanidipine 20mg tablets (a a h pharmaceuticals ltd) | 63917 |
| prazosin 1mg tablets and prazosin 500microgram tablets | 445 |
| amiloride 10 mg tab | 2681 |
| perindopril/tert-butylamine 2 mg tab | 2927 |
| enalapril maleate 40 mg tab | 3509 |

| | |
|---|---|
| frusemide 20mg/spironolactone 50mg mg cap | 3902 |
| triamterene 50mg hydrochlorothiazide25mg tab | 3962 |
| terazosin 2mg tablets and terazosin 1mg tablets | 4694 |
| labetalol tab | 7491 |
| diltiazem hcl xl 300 mg cap | 8024 |
| prenylamine lactate 60 mg tab | 8442 |
| atenolol/chlorthalidone 50 mg tab | 8765 |
| timolol 10mg/bendrofluazide 2.5mg tab | 8788 |
| captopril 100 mg tab | 8923 |
| diltiazem hcl sr 300 mg cap | 9094 |
| sotalol hcl s/r 80 mg tab | 12119 |
| oxprenolol 10 mg tab | 12497 |
| hydrochlorothiazide 12.5mg/k 8.1mmol s/r 12.5 mg tab | 15577 |
| diltiazem hcl s/r 180 cap | 15659 |
| metoprolol fumarate 190 mg tab | 17876 |
| verapamil hcl mr | 18631 |
| carvedilol | 18722 |
| spironolactone/propranolol 50 mg tab | 19003 |
| amiloride 5mg/hydrochlorthiazide 50mg | 19611 |
| amiloride hydrochloride | 19695 |
| amiloride 5mg/hydrochlorthiazide 50mg | 19721 |
| metoprolol 100mg/chlorthalidone 12.5mg | 22151 |
| carvedilol 3.125 mg | 22796 |
| ramipril | 22882 |
| verapamil sr | 23458 |
| hydrochlorothiazide 12.5mg/k 8.1mmol s/r | 23483 |
| sotalol hydrochloride s/r | 23598 |
| verapamil 100 mg tab | 23730 |
| hydrochlorothiazide /metoprolol tartrate 25 mg tab | 24520 |
| atenolol | 24677 |
| nifedipine retard | 25026 |
| atenolol | 25037 |
| nifedipine retard | 25044 |
| nifedipine | 25055 |
| frusemide 40mg/amiloride hyd 5mg | 25075 |
| spironolactone | 25086 |
| pindolol 10mg/clopamide 5mg | 25764 |
| xipamide | 26675 |
| nadolol 80mg/bendrofluazide 5mg mg tab | 27086 |
| enalapril maleate | 27890 |
| metoprolol fumarate 95 mg tab | 28493 |
| diltiazem hydrochloride | 30491 |
| irbesartan | 31160 |
| triamterene 50mg hydrochlorothiazide25mg | 41889 |
| metoprolol 100mg/hydrochlorothiaz.12.5mg | 41892 |
| reserpine/bendrofluazide 150 mcg tab | 41894 |
| timolol 10mg / amiloride 2.5mg / hydrochlorothiazide 25mg tablets | 48745 |
| perindopril tosilate 10mg tablets | 56079 |
| perindopril tosilate 5mg / indapamide 1.25mg tablets | 56157 |
| perindopril tosilate 2.5mg tablets | 57944 |
| perindopril erbumine 8mg / amlodipine 5mg tablets | 60744 |
| perindopril erbumine 8mg / amlodipine 10mg tablets | 63149 |
| bisoprolol 5mg / aspirin 100mg capsules | 65027 |

# Supplementary Material 2

*Description*: Read codes for hypertension

| readterm | readcode |
|---|---|
| Hypertensive disease | G2...00 |
| Essential hypertension | G20..00 |
| Benign essential hypertension | G201.00 |
| H/O: hypertension | 14A2.00 |
| On treatment for hypertension | 662O.00 |
| Hypertension NOS | G20z.11 |
| Hypertensive encephalopathy | G672.00 |
| Seen in hypertension clinic | 9N03.00 |
| Systolic hypertension | G202.00 |
| Hypertension monitoring | 662..12 |
| Hypertensive renal disease | G22..00 |
| Hypertension monitoring admin. | 9OI..00 |
| Hypertensive retinopathy | F421300 |
| Hypertensive disease NOS | G2z..00 |
| Secondary hypertension | G24..00 |
| Borderline hyperten:yearly obs | 6624 |
| BP - hypertensive disease | G2...11 |
| Cardiomegaly - hypertensive | G21z011 |
| Essential hypertension NOS | G20z.00 |
| Excepted from hypertension qual indicators: Patient unsuit | 9h31.00 |
| Excepted from hypertension qual indicators: Informed dissent | 9h32.00 |
| Patient on maximal tolerated antihypertensive therapy | 8BL0.00 |
| Hypertension clinical management plan | 8CR4.00 |
| Hypertension treatm.stopped | 662H.00 |
| Hypertension monitoring | 662P.00 |
| Hypertensive treatm.changed | 662G.00 |
| Hypertensive renal disease NOS | G22z.00 |
| Malignant essential hypertension | G200.00 |
| Secondary hypertension NOS | G24z.00 |
| Hypertensive heart disease NOS | G21zz00 |
| Hypertensive heart disease | G21..00 |
| Good hypertension control | 6627 |
| Antihypertensive therapy | 8B26.00 |
| Hypertension six month review | 662c.00 |
| Moderate hypertension control | 662b.00 |
| Other specified hypertensive disease | G2y..00 |
| Hypertension annual review | 662d.00 |
| Adverse reaction to antihypertensives NOS | TJC7z00 |
| Adverse reaction to other antihypertensives | TJC7.00 |
| Hypertension treatm. started | 662F.00 |

| | |
|---|---|
| Hypertensive heart&renal dis wth (congestive) heart failure | G232.00 |
| Hypertension treatment refused | 8I3N.00 |
| Hypertension monitored | 9OIA.11 |
| Secondary benign renovascular hypertension | G241000 |
| Poor hypertension control | 6628 |
| Hypertension clinic admin. | 9OI..11 |
| Seen in hypertension clinic | 9N1y200 |
| Hypertensive heart and renal disease with renal failure | G233.00 |
| Hyperten.monitor offer default | 9OI3.00 |
| Hypertens.monitor phone invite | 9OI8.00 |
| Renal hypertension | G22z.11 |
| [X] Adverse reaction to other antihypertensives | U60C511 |
| Hypertension:follow-up default | 6629 |
| Hypertens.monitor.1st letter | 9OI4.00 |
| Hypertens.monitor 2nd letter | 9OI5.00 |
| Hypertens.monitor 3rd letter | 9OI6.00 |
| Hypertension secondary to drug | G24z100 |
| Secondary renovascular hypertension NOS | G24z000 |
| Hypertensive heart disease NOS | G21z.00 |
| Secondary malignant hypertension | G240.00 |
| Hypertensive crisis | G672.11 |
| Hypertensive renal disease with renal failure | G222.00 |
| Hypertension induced by oral contraceptive pill | 6146200 |
| Exception reporting: hypertension quality indicators | 9h3..00 |
| Hypertens.monitoring admin.NOS | 9OIZ.00 |
| DNA - Did not attend hypertension clinic | 9N4L.00 |
| Hypertension secondary to endocrine disorders | G244.00 |
| Hypertension monitor.chck done | 9OIA.00 |
| Blind hypertensive eye | F404200 |
| Malignant hypertensive renal disease | G220.00 |
| Hypertens.monitor verbal inv. | 9OI7.00 |
| Secondary hypertension NOS | G24zz00 |
| Refuses hypertension monitor. | 9OI2.00 |
| Pre-eclampsia or eclampsia with pre-existing hypertension | L127.00 |
| Benign hypertensive renal disease | G221.00 |
| [X] Adverse reaction to antihypertensives NOS | U60C51A |
| Pre-exist hypertension compl preg childbirth and puerperium | L128.00 |
| Attends hypertension monitor. | 9OI1.00 |
| Malignant hypertensive heart disease | G210.00 |
| Secondary benign hypertension NOS | G241z00 |
| Benign hypertensive heart disease with CCF | G211100 |
| Benign hypertensive heart disease | G211.00 |
| Pre-exist 2ndry hypertens comp preg childbth and puerperium | L128200 |
| Secondary benign hypertension | G241.00 |
| Hyperten heart&renal dis+both(congestv)heart and renal fail | G234.00 |
| Secondary malignant renovascular hypertension | G240000 |

| | |
|---|---|
| Pre-exist hyperten heart dis compl preg childbth+puerperium | L128000 |
| Hypertensive heart disease NOS without CCF | G21z000 |
| Benign hypertensive heart disease without CCF | G211000 |
| Other pre-existing hypertension in preg/childb/puerp NOS | L122z00 |
| Hypertensive heart disease NOS with CCF | G21z100 |
| Benign hypertensive heart and renal disease | G231.00 |
| [X]Oth antihyperten drug caus advers eff in therap use, NEC | U60C500 |
| Hypertensive heart and renal disease | G23..00 |
| Other pre-existing hypertension in preg/childbirth/puerp | L122.00 |
| Malignant hypertensive heart and renal disease | G230.00 |
| Hypertensive heart and renal disease NOS | G23z.00 |
| [X]Hypertensive diseases | Gyu2.00 |
| Other pre-existing hypertension in preg/childb/puerp - deliv | L122100 |
| Malignant hypertensive heart disease with CCF | G210100 |
| Secondary malignant hypertension NOS | G240z00 |
| Other pre-existing hypertension in preg/childb/puerp unspec | L122000 |
| Diastolic hypertension | G203.00 |
| High cost hypertension drugs | 7Q01.00 |
| Pre-eclampsia or eclampsia + pre-existing hypertension NOS | L127z00 |
| Malignant hypertensive heart disease without CCF | G210000 |
| Trial withdrawal of antihypertensive therapy | 662r.00 |
| Other pre-exist hypertension in preg/childb/puerp-not deliv | L122300 |
| [X]Hypertension secondary to other renal disorders | Gyu2100 |
