# Supplementary material for: Trends for prevalence and incidence of resistant hypertension: population based cohort study in the UK 1995-2015
Source: BMJ. 2017 Sep 22;358:j3984. doi: 10.1136/bmj.j3984 (PMC5609092; doi:10.1136/bmj.j3984)
Supplement: Supplementary file 2 — Supplementary material: supplementary material 3-9 [file sins038885.ww2.pdf]

Supplementary Material 3

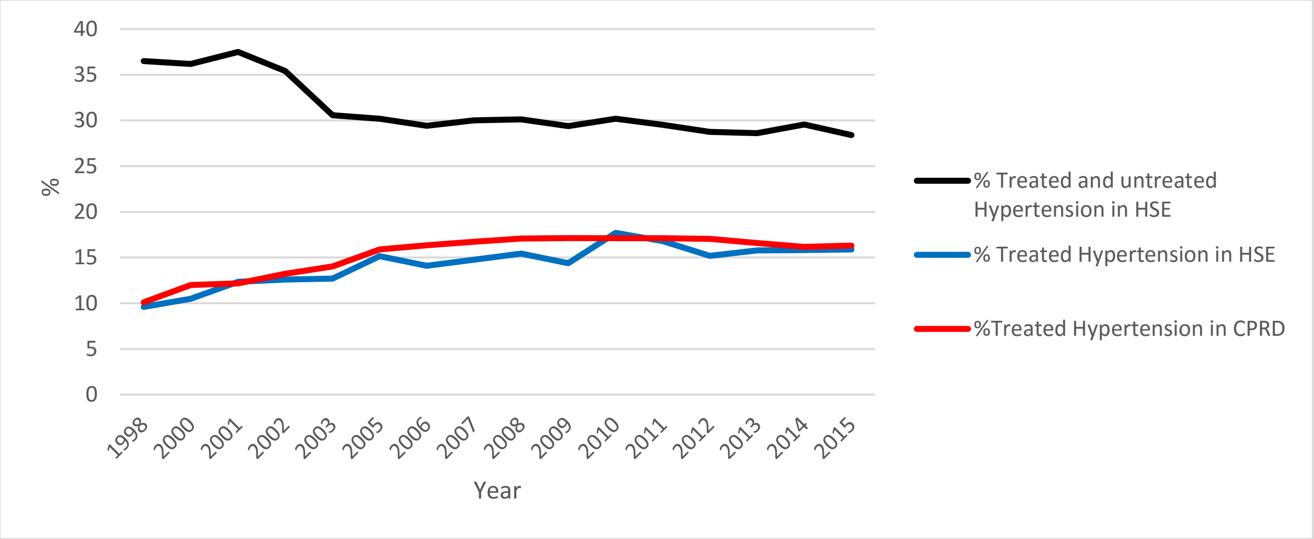

Figure S3.1: Prevalence rates of treated hypertension in CPRD *vs* national prevalence rates of treated hypertension and treated and untreated hypertension from Health Survey for England.

HSE: Health Survey for England
CPRD: Clinical Practice Research Datalink

Supplementary Material 4

Figure S4.1: Joinpoint model for incidence of resistant hypertension 1996-2015

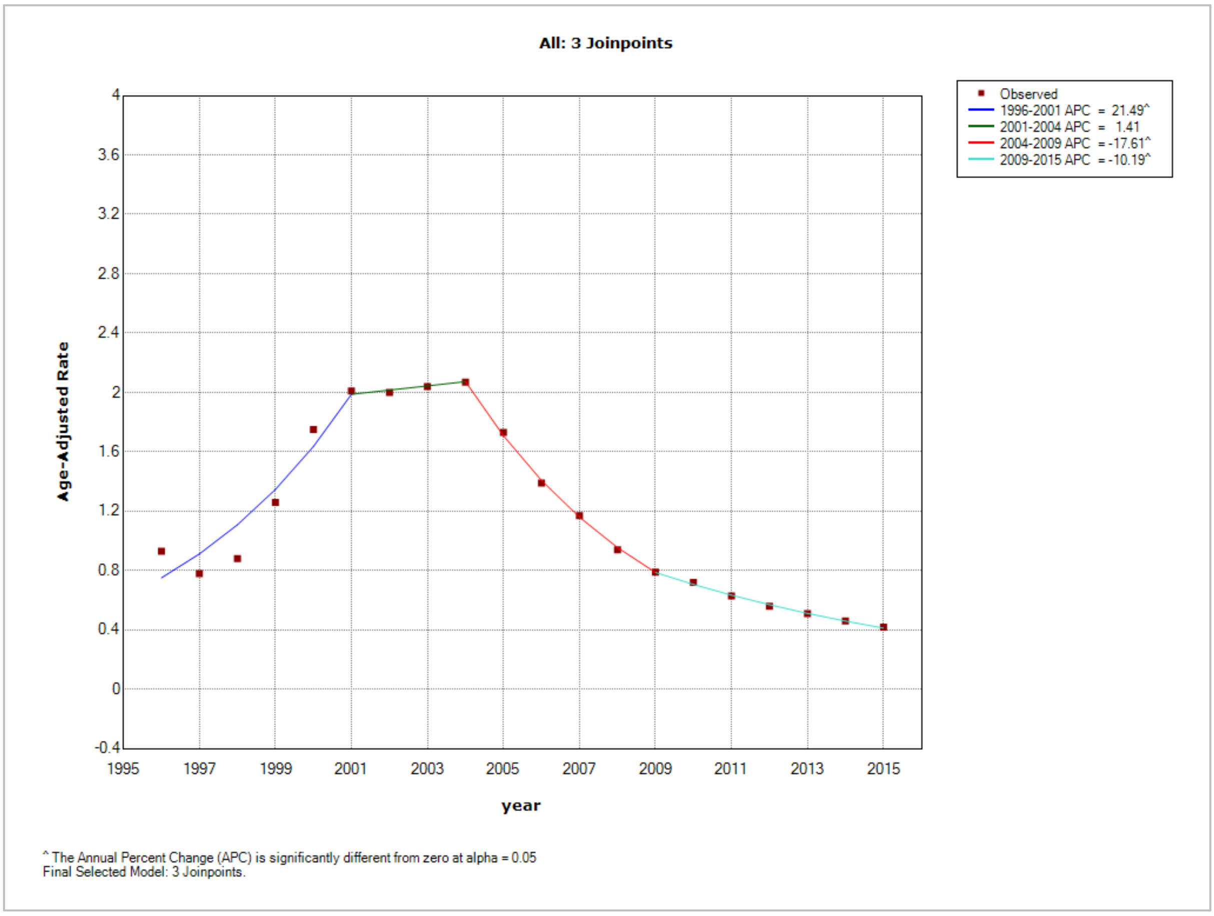

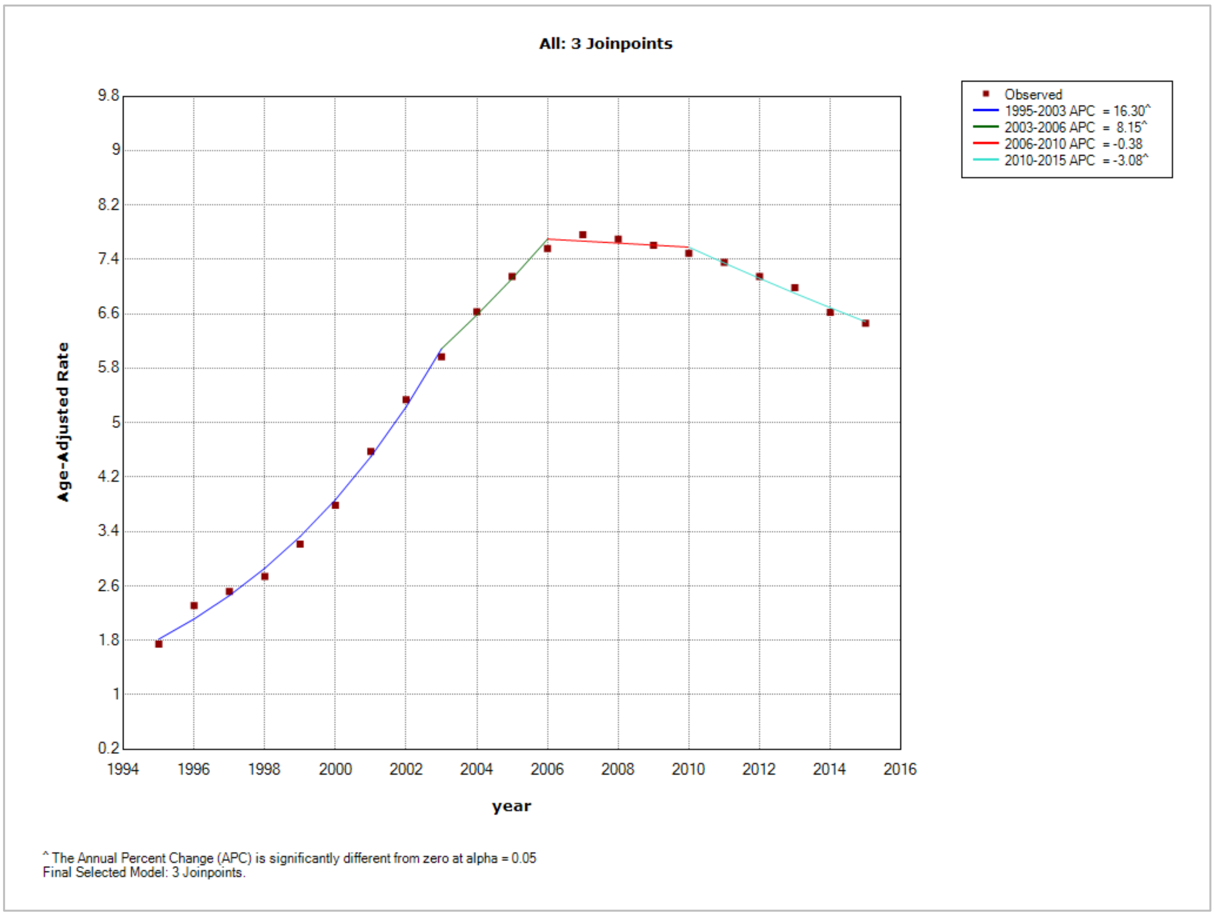

Figure S4.2 Joinpoint model for prevalence of resistant hypertension 1995-2015

Supplementary Material 5

Table S5.1: Age and sex adjusted prevalence and incidence estimates for resistant hypertension

	Incidence Rate Resistant Hypertension per 100 person years (95% CI)	Prevalence Resistant Hypertension per 100 people (95% CI)
**Gender (ref==male)**		
Female	0.98 (0.96 – 0.99)	1.01 (1.00 – 1.03)
**Age category (years)**		
< 50	0.69 (0.66 – 0.71)	0.44 (0.43 – 0.46)
50- 59	0.81 (0.79 – 0.84)	0.68 (0.66 – 0.69)
60-64	0.93 (0.91 – 0.96)	0.85 (0.84 – 0.87)
65-69	*ref*	*ref*
70-74	1.12 (1.09 – 1.14)	1.16 (1.15 – 1.18)
75-79	1.19 (1.16 – 1.23)	1.33 (1.31 – 1.35)
80+	1.07 (1.04 – 1.10)	1.43 (1.39 – 1.46)

All analyses adjusted for year.
Denominator = hypertensive population

Supplementary Material 6

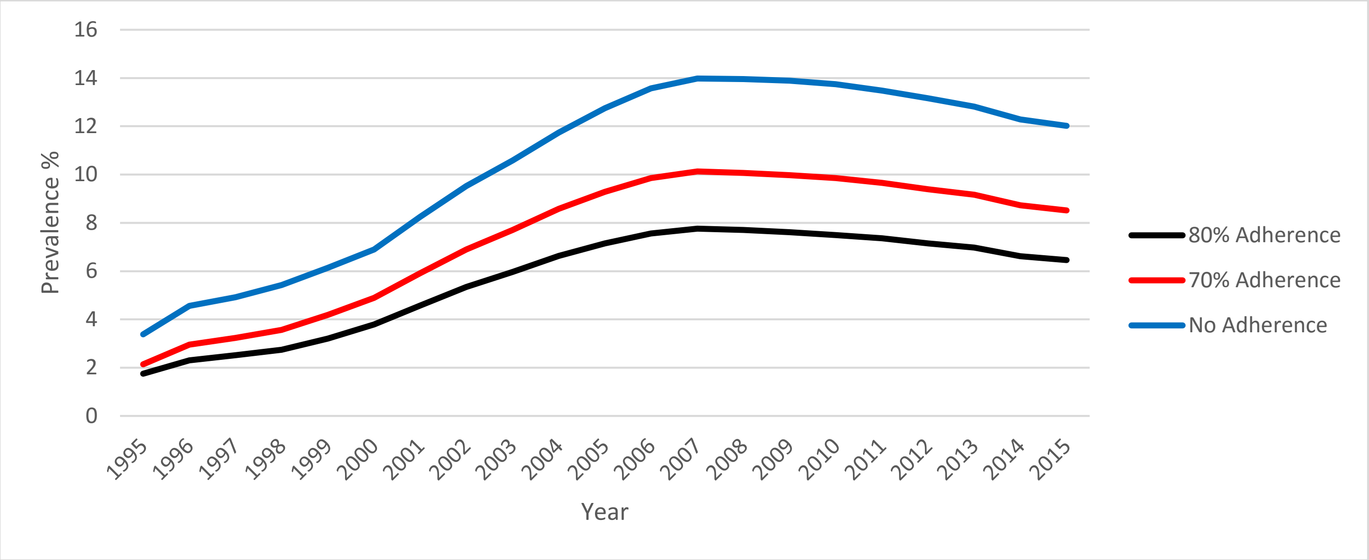

Figure S6.1: Prevalence of resistant hypertension when adherence defined at 80%, 70% and not accounted for.

Trends mirror those when 80% threshold is used, with slightly higher peak prevalence in 2007 for 70% threshold (10.13, 95% CI 10.60 - 10.21 *vs* 7.76, 95% CI 7.70 – 7.83) and higher peak prevalence in 2007 when adherence not accounted for (13.98, 95% CI 13.90 – 14.07 *vs* 7.76, 95% CI 7.70 – 7.83).

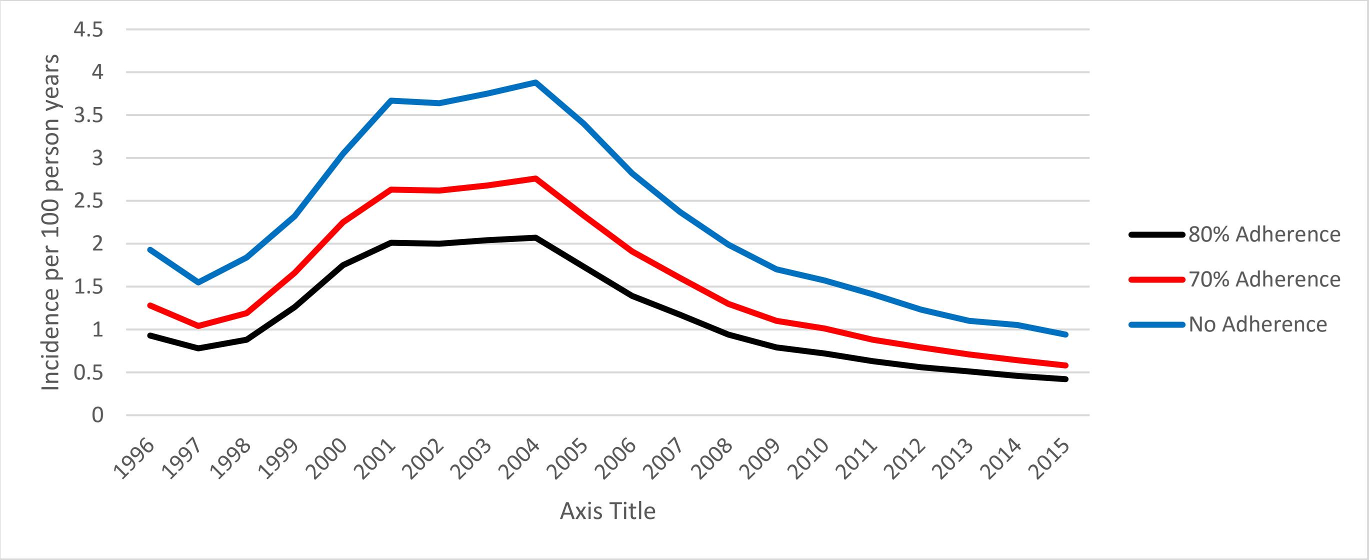

Figure S6.2: Incidence of resistant hypertension when adherence defined at 80%, 70% and not accounted for.

Trends mirror those when 80% threshold is used, with slightly higher peak incidence in 2007 for 70% threshold (2.91, 95% CI 2.87 - 2.96 *vs* 2.07, 95% CI 2.03 – 2.12) and higher peak incidence in 2004 when adherence not accounted for (3.88, 95% CI 3.82 – 3.94 *vs* 2.07, 95% CI 2.03 – 2.12).

Supplementary Material 7

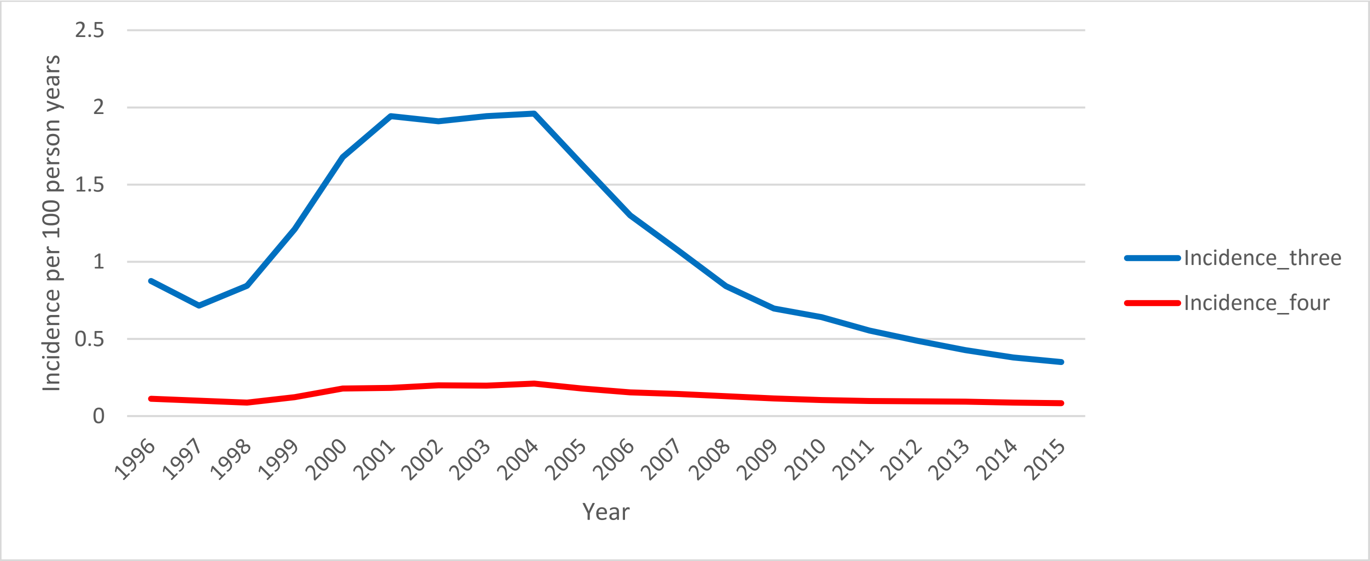

Figure S7.1: Stratification of incidence rates by three and four drug regimens.

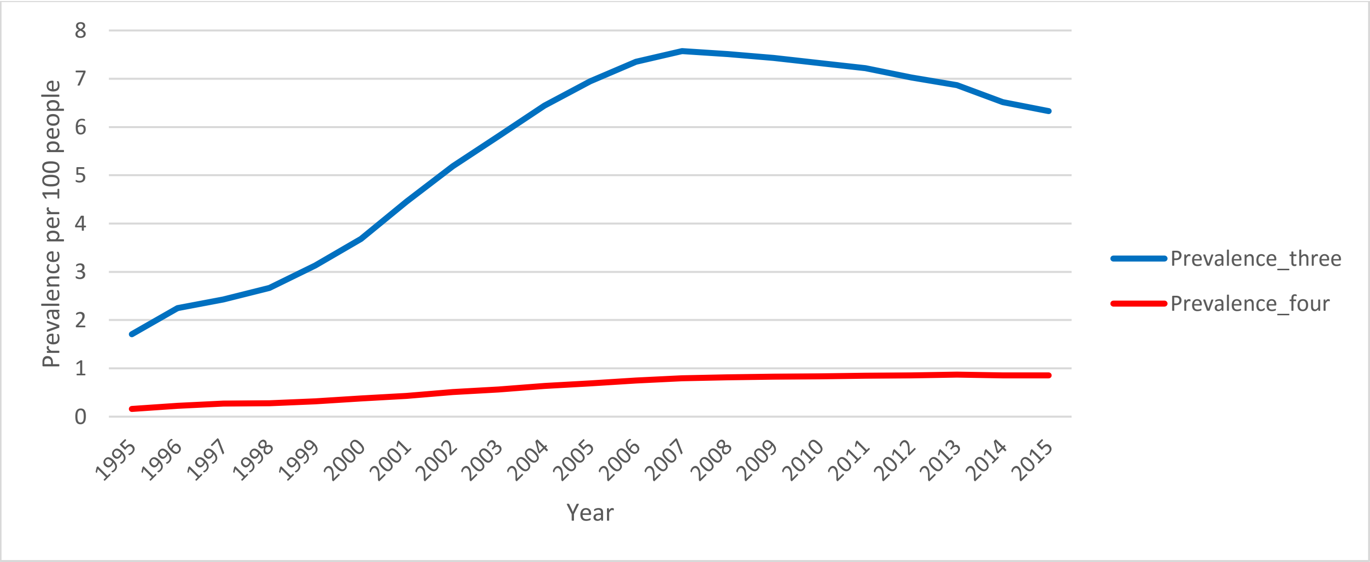

Figure S7.2: Stratification of prevalence by three and four drug regimens.

Supplementary Material 8

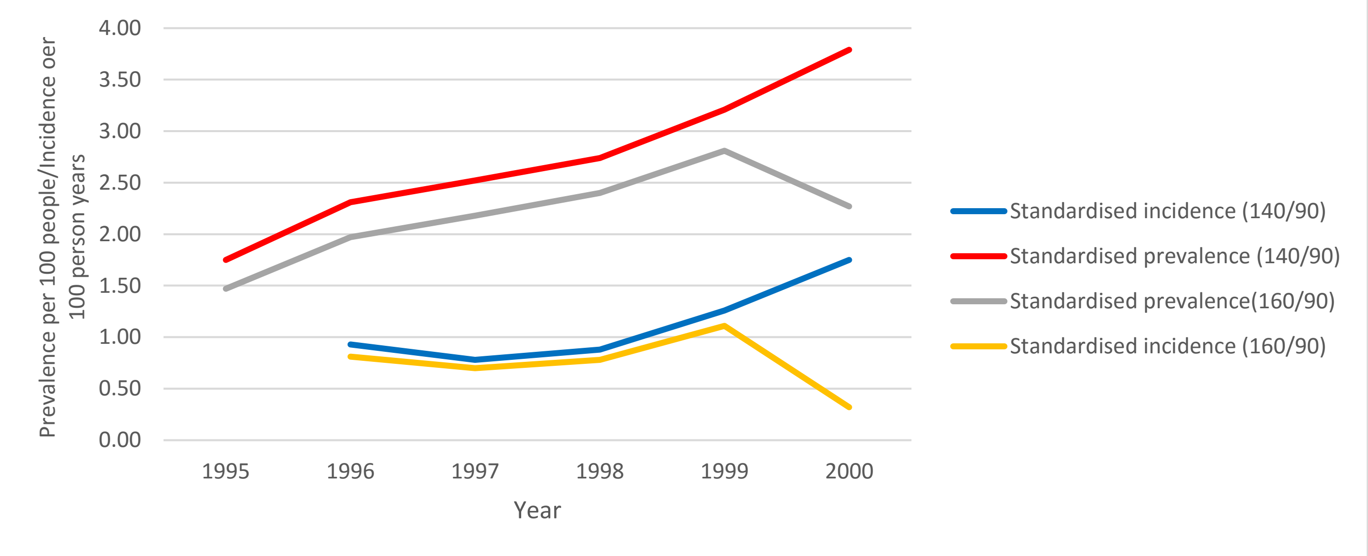

Figure S8.1: Trends for prevalence and incidence of resistant hypertension using two thresholds for definition of hypertension: ≥160/90mmHg (1993 guidelines) and ≥140/90mmHg (threshold for entire study).

Explanation: Trends for each threshold closely follow one another, although the higher threshold (≥160/90mmHg) has a lower prevalence and incidence rate, as expected. The higher threshold also sees a dip after 1999; this is because new guidelines from the British Hypertension Society (Ramsay *et al.*) were published in 1999 which recommended optimal targets of <140/85mmHg.

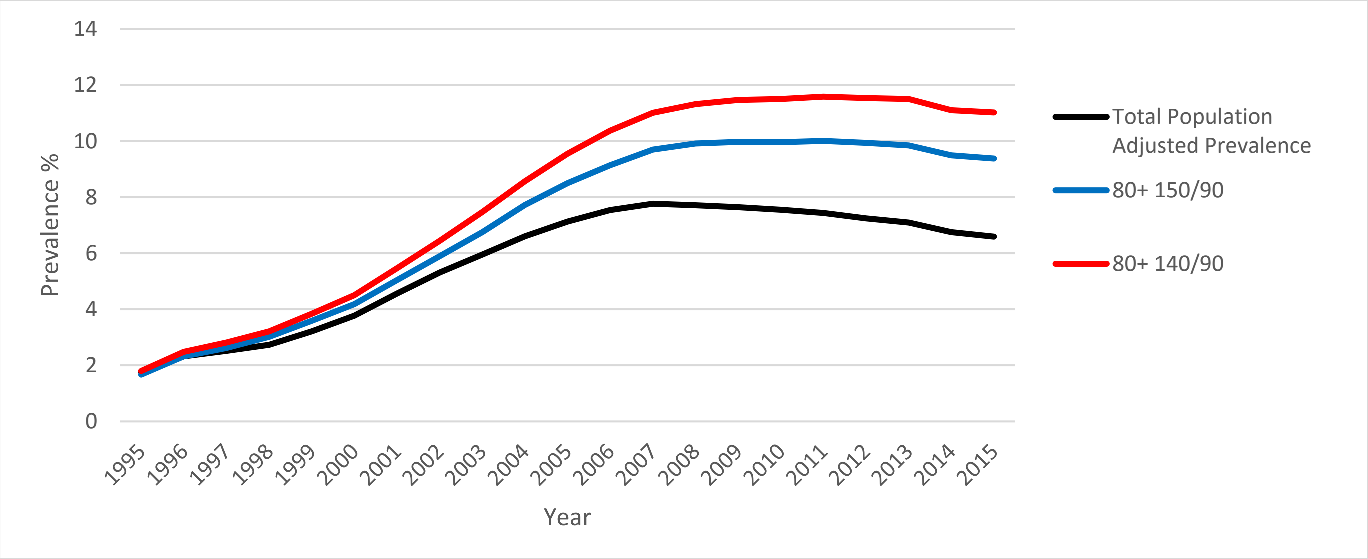

Figure S8.2: Crude trends for prevalence and incidence of resistant hypertension using two thresholds for definition of hypertension for those aged ≥80 years: ≥150/90mmHg (2011 guidelines) and ≥140/90mmHg (threshold for entire study). The "Total Population Adjusted Prevalence" is based on a 140/90mmHg threshold.

Explanation: In the main analysis, there was evidence of interaction between age and time for the prevalence of resistant hypertension. In other words, those aged ≥80 years appeared to have increasing prevalence over time in contrast to the reference age group. This increased prevalence may have occurred because the threshold used in the analysis was ≥140/90mmHg, whereas clinical guidelines recommend higher thresholds for those in older age groups (≥150/90mmHg). When we redefined resistant hypertension according to a ≥150/90mmHg threshold in the ≥80 years age group, the increase in prevalence over time persists, but is not as marked as the trend presented in the main analysis.

Supplementary Material 9

Table S9.1: Distribution of secondary causes of resistant hypertension and hypertension amongst those with resistant hypertension in CPRD 1995-2015

	**Resistant Hypertension n= 92,811**
**eGFR category (ml/min/1.73m^2)***	
≥60	44190 (47.6)
45-59	19298 (20.8)
30-44	9375 (10.1)
15-29	2606 (2.8)
<15	467 (0.5)
Missing	16875 (18.2)
**Glomerulonephritis**	279 (0.3)
**Nephropathy**	470 (0.5)
**Polycystic Kidney Disease**	108 (0.1)
**Pyelonephritis**	658 (0.7)
**Renal artery stenosis**	221 (0.2)
**Coarctation of aorta**	35 (0.04)
**Sleep apnoea**	850 (0.9)
**Phaeochromocytoma**	17 (0.02)
**Conn's syndrome**	67 (0.1)
**Cushing’s syndrome**	65 (0.1)

*Calculated from most recent creatinine measurement within 12 months before the index date.
